# Relevant Dermatoses Among U.S. Military Service Members: An Operational Review of Management Strategies and Telemedicine Utilization

**DOI:** 10.7759/cureus.33274

**Published:** 2023-01-02

**Authors:** Gehan A Pendlebury, Peter Oro, Kerstyn Ludlow, Drew Merideth, William Haynes, Vikas Shrivastava

**Affiliations:** 1 Dermatology, Nova Southeastern University Dr. Kiran C. Patel College of Osteopathic Medicine, Davie, USA; 2 Internal Medicine, School of Osteopathic Medicine in Arizona, A.T. Still University, Mesa, USA; 3 Medical Student, Arizona State University, Tempe, USA; 4 Emergency Medicine, School of Osteopathic Medicine in Arizona, A.T. Still University, Mesa, USA; 5 Radiology, School of Osteopathic Medicine in Arizona, A.T. Still University, Mesa, USA; 6 Dermatology, Navy Medicine Readiness Training Command, Naval Medical Center San Diego, San Diego, USA

**Keywords:** military readiness, military medicine, teledermoscopy, teledermatology, inflammatory skin disease, skin cancer, dermatological conditions, cutaneous infections, occupational skin disease, operational dermatology

## Abstract

Despite skin being the largest and most exposed organ of the human body, skin issues can be challenging to diagnose in deployed military service members. Common reasons deployed soldiers seek dermatological evaluation include infections, inflammatory skin conditions, and skin growth. Due to limited access to specialized care in deployed settings, dermatological conditions are undertreated and underdiagnosed. As a result, dermatological conditions are a leading contributor to decreased combat effectiveness among deployed medical forces. To lessen the burden of dermatological diseases, military providers should promptly identify operational skin diseases and alleviate modifiable barriers faced by service members. In a post-pandemic era with novel Severe Acute Respiratory Syndrome Coronavirus 2 (SARS-CoV-2) and monkeypox infections, the duty to effectively treat operational skin lesions is ever important. The need for military dermatologists continues to rise as the global landscape continues to evolve with unprecedented infections and increased bioterrorism threats. Teledermatology offers many solutions to mitigate the high demand for dermatologists during pandemics. Dermatological consultations account for the highest number of telemedicine visits in the US Military Health System (MHS). As such, increased utilization of teledermatology will reduce infection-related dermatological sequelae and prevent the medical evacuation of service members from military operations. This review collates and categorizes relevant dermatological conditions encountered among deployed personnel. This report outlines the standard of care and modified treatments recommended according to potential barriers faced in operational settings.

## Introduction and background

Background

Despite skin being the most exposed organ of the human body, skin issues are difficult to identify in military service members in operational settings. The most widespread reasons soldiers seek dermatological consultation and treatment during deployment include warts, infections (fungal and bacterial), acne, eczematous conditions, urticaria, and psoriasis. Such cutaneous conditions are often underdiagnosed, undertreated due to limited access to specialists during military operations, or dismissed due to the subacute nature . Due to restrictions on medication use and eligibility requirements for specific conditions, military personnel with dermatological conditions encounter significant obstacles in the treatment [1]. Therefore, it is prudent that military dermatologists are accessible for a consultation to assist in accurate diagnosis. Timely diagnosis improves treatment outcomes, particularly in an operational setting [1-3].

Operational settings often consist of exposure to harsh environmental conditions, insects, and contaminated surroundings. These adverse factors lead to an increased susceptivity to dermatological disorders. Furthermore, specific geographical locations can increase the risk of various occupational skin diseases. For example, US military personnel stationed in Southeast Asia during World War II experienced an increase in melanoma and nonmelanoma skin cancer [4]. In hot and damp locations such as Vietnam, increased incidence of warts, acne, and fungal infections were also observed. In dryer locations, such as Bosnia or Iraq, military personnel experienced increased rates of eczematous conditions [2].

A multitude of factors increase the limitations of proper dermatological care in deployed settings. On a macroscopic level, the availability of military dermatologists has decreased since the National Defense Authorization Act of 2017 [3]. Furthermore, restrictions on medication use and eligibility requirements prevent military personnel with specific, treatment-resistant dermatological diseases from maintaining mission readiness [3,5]. Such critical considerations make it increasingly prudent to accurately identify and promptly treat occupational skin disease to prevent mission evacuation.

Largely due to occupational skin disease, mission evacuation of service members for healthcare-related reasons has contributed to dwindling numbers of personnel in the past [6]. To maintain medical readiness and combat effectiveness, military personnel must be able to perform their duties without hindrance by medical conditions. Due to limited availability of military dermatologists, service members commonly see primary care providers, which can lead to misdiagnosis and ineffective treatment [1].

Introduction

Dermatological conditions are common among military service members. In comparison to civilians, active-duty personnel are at an increased risk of infections, wounds, burns, and acne [7]. Military service members also have increased exposure to heat, wool, and tropical climates, all of which contribute to dermatological issues [7,8]. Between 9-40% of calls for a physician during recent wars have been for dermatological reasons [2]. However, access to dermatologists during deployment remains limited, preventing military personnel from receiving the same level of care they would receive on US soil [1].

Dermatological conditions decrease deployment eligibility, military readiness, and combat effectiveness. Prompt and appropriate medical care can prevent military personnel from evacuation or from being deemed ineligible to deploy [3]. The military has implemented teledermatology in its training facilities to provide accessible and cost-effective dermatological consultations in remote settings. Since the inception of military telemedicine, teledermatology has accounted for the most consultations annually, the highest of any specialty. The advancement of teledermatology practice in the military continues to improve combat effectiveness of military warfighters [9].

We herein present a comprehensive review that examines frequent skin disorders faced by military personnel. Furthermore, our analysis underscores the utility of military teledermatology and its increasingly necessary role in mobilizing dermatological care in a pandemic era [1,2].

Methods

Research databases were searched for articles that discussed dermatological manifestations encountered among active-duty service members in various world regions. Selection criteria for dermatological conditions prioritized relevant and prevalent manifestations encountered in a deployed setting. Three investigators (GP, KL, and PO) reviewed the included studies independently and selected the articles suitable for the review. 

The literature review was conducted by searching PubMed and MEDLINE databases. The following key terms were utilized to locate publications on the topic: “skin” OR “eczema” OR “psoriasis” OR “melanoma” OR “urticaria” OR “fungal infection” OR “nevus” OR “dermatitis” OR “alopecia” OR “actinic keratosis” OR “bacterial infection” OR “warts” AND “military” OR “active duty” OR “deployment” AND “dermatology”. Search terms were selected according to appropriateness and relevance in consideration of the purpose of this literature review. Meta-analyses, systematic reviews, prospective studies, retrospective studies, literature reviews, case series, and case reports were included in the search. Literature published between the years 2000 and 2022 were included. Articles written in a language other than English were excluded. The search excluded military service members outside of the US military.

In addition to searching databases, several articles were located using the Snowball Method. Articles were initially screened according to the title and abstract relevance to the topic. All authors individually conducted a thorough analysis of the included literature. The literature search retrieved 3,994 articles that were initially screened by three reviewers (GP, KL, and PO). Articles were screened according to the title and abstract. Articles were excluded if they did not contain any of the specified key terms in their title or abstract. Following the initial screening, meta-analyses, systematic reviews, prospective studies, retrospective studies, books, topic reviews, case series, and case reports were included. 

Results

**Figure 1:**
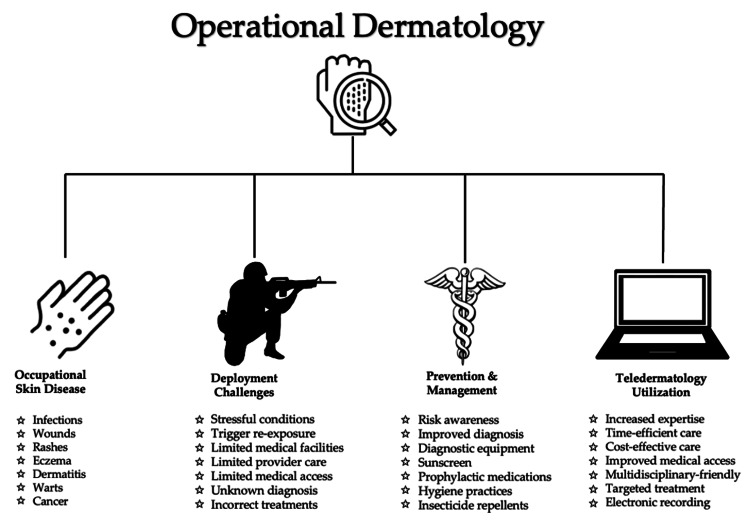
Overview of operational dermatology

Dermatological conditions encountered in deployed settings are challenging to treat. Factors that limit treatment include scarce resources, lack of access to specialist care, prolonged training hours, and austere environmental conditions. Compliance with treatment can be an additional obstacle due to active-duty responsibilities. Furthermore, strict military standards regarding clothing attire and hairstyle restrictions impede treatment compliance. The utilization of teledermatology and teledermoscopy has been shown to decrease morbidity and mortality related to dermatological conditions and improve combat readiness. Access to specialized dermatological care empowers deployed primary care providers to improve lesion identification, accurate diagnosis, and provide timely treatments. Teleconsultations with military dermatologists facilitate successful clinical management and support operational effectiveness. To maintain mission preparedness, military personnel must be able to perform their duties without the hindrance of occupational skin disease. Figure 1 depicts key factors in operational dermatology: occupational skin disease, deployment challenges, prevention/management, and teldermatology utilization.

## Review

Bacterial infections

Human skin serves as a barrier to prevent the entry of bacteria and debris. When this barrier is disrupted, it can lead to the development of skin infections [10]. Common bacterial infections include impetigo, erysipelas, cellulitis, abscesses, and bacterial folliculitis. In the military, skin and soft tissue infections (SSTIs) are of increased concern due to communal living and close contact with others. Additionally, military training predisposes service members to harsh physical environments. These austere physical conditions can induce skin abrasions and traumatic wounds leading to various bacterial skin infections [11].

Between January 2016 and September 2020, there were 210,914 diagnosed cases of SSTIs in the US military [12]. Of these diagnosed cases, cellulitis and abscesses accounted for 64.5%, impetigo and folliculitis compromised 30% collectively, carbuncles and furuncles comprised 5.3%, and erysipelas comprised 0.2% of total SSTIs [12]. The prevalence of SSTIs varies between military branches due the differing types of missions, training drills, and deployment locations [12].

Beyond other barriers to prevention, SSTIs are more common in military trainees due to the nature of communal living in close quarters [13]. Despite the implementation of proper hygiene practices, SSTIs remain challenging to treat due the high-risk nature associated with community settings. For example, military trainees experience environmental conditions that predispose them to methicillin-resistant *Staphylococcus aureus* (MRSA) and other treatment resistant SSTIs [13].

A prospective, two-year cluster-randomized controlled trial (RCT) evaluated 30,209 US Army Infantry trainees to analyze the effects of personal hygiene-based interventions on the rates of overall SSTIs and MRSA SSTIs [12]. Army recruits were divided into three study groups, which were divided by increasing levels of education and hygiene-based treatment interventions. The study groups were designated as standard, enhanced standard, and chlorhexidine [12]. This RCT concluded that despite personal hygiene and educational measures (including weekly use of chlorhexidine body wash), overall SSTIs and MRSA SSTIs were not prevented in the high-risk population of military trainees. This Army trial also revealed that during the first two years of military service, SSTIs are the leading infectious etiology of hospital admission, surpassing hospitalization rates for influenza and pneumonia [13].

Erysipelas

**Figure 2:**
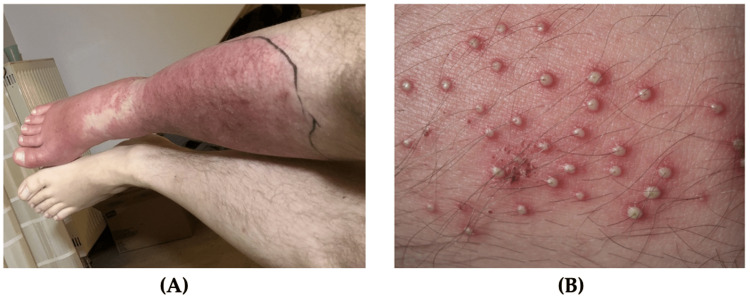
Common bacterial soft tissue infections (A) Erysipelas, a sharply demarcated erythematous rash with discrete narrowing extension, is visible on the lower leg; (B) Folliculitis, multiple pustules with surrounding erythema can be seen emerging from the hair follicles Source: Figure 2A: Wundrose am Unterschenkel by Failing79, Wikimedia Commons, licensed under CC BY-SA 4.0; Figure 2B: Folliculitis by Da pacem Domine, Wikimedia Commons, licensed under CC0 1.0

Erysipelas is an infection that affects the upper dermis with extension to the lymphatics. Erysipelas diagnosis is based on clinical presentation and medical history [14]. It is commonly caused by beta-hemolyticstreptococcus[10,11]. Erysipelas presents as an acute-onset rash that is raised, tender and hot to touch with circumscribed erythema and surrounding edema (Figure 2A) [15]. Erysipelas affects all ages and most commonly occur on the face, ears, and legs [10]. Patients with erysipelas are more likely to experience pain, nausea, and fever [14]. With regards to military personnel, it is most common to develop erysipelas of the legs [11]. If treated early, this condition does not have a significant impact on military readiness. However, if left untreated erysipelas may cause significant discomfort and pain with the debilitating progression of the disease [9-13]. Severe cases of erysipelas can warrant time off from operational missions. Risk factors include an immunocompromised state and impaired venous or lymphatic drainage. Erysipelas is sometimes difficult to distinguish from cellulitis, but erysipelas develops more rapidly with better-defined margins [15].

Bacterial Folliculitis

Folliculitis is defined as inflammation of hair follicles or sebaceous glands. Folliculitis is diagnosed based on physical exam findings of tender papules or pustules at the site of the hair follicle [16,17]. The incidence of folliculitis is unknown because most patients do not see a physician for diagnosis and treatment [16,17]. The cause of bacterial folliculitis is most often *Staphylococcus aureus *(*S. aureus*), which infiltrates hair follicles of the face and scalp after follicular trauma or occlusion [18]. Bacterial folliculitis appears as clusters of raised, pruritic, erythematous papules and pustules typically < 5 mm in diameter and surround a central hair (Figure 2B). These lesions are typically not painful. Predisposing factors for this condition include shaving, applying topical oils, and occupational exposure to solvents or tars [18]. The grooming standards for short hair and clean-shaven faces in the military increase prevalence of bacterial folliculitis [19]. Soldiers can apply for a medical waiver to keep their hair or beards longer to avoid this condition. Therefore, educating patients on available options is essential. Additionally, obtaining these waivers can be burdensome as they must be renewed annually. Moreover, unshaven facial hair can be negatively perceived by peers and leadership [19]. 

Impetigo

Impetigo is a contagious skin infection of the epidermis and is diagnosed clinically [20]. Prevalence for impetigo in the general population is 11-15% [21]. This condition is typically caused by *S. aureus* [22]. The bacteria infect intact and compromised skin. Causes of compromised skin include trauma, insect bites, eczema, or herpetic lesions [23]. The two subtypes of impetigo are bullous and non-bullous. The bullous subtype most commonly affects children and adults to a lesser extent. It is most often found in the axillae, neck, and diaper. Bullous impetigo typically first presents as small vesicles that become flaccid bullae [10,24]. Patients experience generalized malaise, diarrhea, and a low-grade fever. Non-bullous impetigo makes up 70% of cases of impetigo and affects patients of any age [22]. These lesions most often affect the limbs and face and present with vesicles that rupture to form a painful, itchy, honey-yellow crust over the base [22]. Systemic symptoms are usually absent in this subtype. It is also important to note that regional lymphadenopathy is usually present in the non-bullous variant but absent in the bullous variant [10,24]. Impetigo is more common in warm climates but also presents in the summer months of colder climates. This infection frequently develops in traumatic skin lesions from training. Impetigo is a contagious condition and can be passed between soldiers most commonly during hand-to-hand combat training. Impetigo can cause discomfort and may cause military personnel to take extended breaks from training to avoid spread [11]. Risk factors for this type of infection include lack of hygiene, poverty, and cramped areas [10].

Cellulitis

Cellulitis manifests as a skin infection affecting deep layers of the dermis and underlying fat. It is often described as a more severe form of erysipelas, and therefore, their prevalence is combined. The prevalence of erysipelas and cellulitis in emergency rooms in the US from 2006 to 2016 was 2.42 to 3.55 per million [25]. Due to the ability to effectively manage such conditions at home, the overall prevalence is difficult to determine. Likewise, it is challenging to determine the prevalence in various outpatient and inpatient settings. Diagnosis of cellulitis is based on clinical presentation and physical examination [26]. An annual estimate of 14.5 million cases of cellulitis has been reported in the US [27].

Cellulitis typically presents as a unilateral, poorly demarcated erythema and edema with warmth and tenderness [10,26]. Severe cases have been shown to present with fever, rapid extension of erythema, and progression of systemic symptoms despite ongoing oral antibiotics. Regional lymphadenopathy and lymphangitis are usually present in the condition [27]. If patients present with rapid progression of the infection and pain out of proportion to the apparent clinical signs, necrotizing fasciitis must be ruled out [26]. Approximately 29% of the non-battle injuries reported in the US Army medical facility result from cellulitis. Also, cellulitis is more common during military training as high stress on the mind and body leads to compromised immune function [10]. Cellulitis due to friction blisters is common in the military population due to frequent exercise and physical activity [11]. Risk factors include broken skin, obesity, immunocompromised patients, edema from venous insufficiency or lymphatic obstruction, recent surgery, IV drug use, and previous episodes of cellulitis [9,26].

Abscesses

Abscesses are a common complication of cellulitis. An abscess is described as a purulent sore in the dermis and deep skin tissues. Diagnosis is made based on physical examination and medical history [28]. The incidence of ambulatory visits for purulent SSTIs was 12.8 per 1,000 in 2015 [29]. Abscesses are commonly caused by *S. aureus* and other bacterial infections. Abscesses are a collection of leukocytes, bacteria, and cellular debris. Neutrophils surround the infection and release cytokines which induce the destruction of bacterial cell walls and the release of toxins. Cytokines and bacterial toxins are involved in the inflammatory process and the formation of pus [10]. Abscesses often appear below the epidermis or in the lumen of a hair follicle and are frequently found in the axillae, buttocks, perineum, groin, or breasts [30]. These lesions appear as painful, fluctuant, erythematous nodules usually surrounded by erythematous swelling. Risk factors for the development of abscesses include contact with others with active abscesses, recent pedicures, traumatic wounds, recent surgery, and cellulitis [27-29].

Necrotizing Fasciitis (NF)

NF is a rapidly progressing skin infection that leads to necrosis of the skin, subcutaneous tissue, and fascia [31]. Like other bacterial infections, NF is diagnosed clinically. If NF is suspected, the ‘finger test’ is performed, which involves using a localized anesthetic and making a 2 cm incision down to the deep fascia [32]. If the index finger is easily able to dissect the subcutaneous tissue from the deep fascia, the test is positive. Patient history is also important in this condition due to exposure to water, recent traumas, or underlying conditions. The estimated rate of NF in the US is 0.40 cases per 100,000 people [33]. NF is more common in men than women and more likely with advanced age. If NF is suspected, cultures and sensitivities are performed such that proper treatment can be started as quickly as possible. If severe sepsis develops, NF has a 20-50% mortality rate [11,31]. Common causes of NF include *S. pyogenes*, *S. aureus*, Vibrio species, or polymicrobial combinations [33]. NF initially presents with extreme tenderness, erythema, swelling, and pain. If left untreated, bullae, crepitus, and necrosis of the skin may develop. Additionally, patients present with fever, hypotension, and tachypnea. NF can manifest at any location on the body but is more common in the extremities, trunk, and perineum [33]. NF has been present in the US military since at least the civil war due to traumatic injuries, but the bacteria responsible have likely changed. This condition has a massive impact on combat readiness as it is a medical emergency. Complications of this condition may occur which require immediate evacuation from the operational mission. Multidrug-resistant organisms are now common causes of NF, so it is necessary to keep this in mind during treatment. Predisposing conditions include diabetes, malignancy, alcohol abuse, and chronic liver or kidney disease [32,33]. Environmental risk factors for NF include recent surgery or travel, trauma, bacterial infection (impetigo), or seawater exposure [32,33].

Standard of Treatment

Most SSTIs can be treated with antibiotics and antiseptic products. However, antibiotic resistance is a significant concern and should be considered when treatment fails to clear the infection [10]. Treatment for bacterial skin infection depends on the severity, type, location, and size of the infection [10,16]. 

Folliculitis: Staphylococcal folliculitis usually resolves spontaneously without treatment. However, it is recommended to wash the affected area with antimicrobial cleansers such as benzoyl peroxide [18]. Lesions persistent for more than several weeks require antibiotics such as topical mupirocin (three times daily for five to seven days) or topical clindamycin gel, lotion, or solution (twice daily for seven to 10 days). Severe cases refractory to topical antibiotics require culture for antibiotic sensitivity and specificity [16-19]. If MRSA is suspected, the suggested treatments include doxycycline (100 mg twice daily), trimethoprim-sulfamethoxazole (TMP-SMX) (one-to-two double-strength tablets twice daily), and clindamycin (450 mg three times per day). Additional remedies include warm salt compress, bacitracin, or erythromycin ointment with gauze dressings [18]. Furthermore, scalp folliculitis can be treated with antibacterial shampoo [19]. Patients with severe or refractory bacterial folliculitis can be treated with a seven-day course of cephalosporins, dicloxacillin, or flucloxacillin [16]. 

Impetigo: Impetigo is a common soft tissue infection that often heals spontaneously within two weeks [20]. It is managed primarily with improved hygiene practices such as regular cleaning of lesions with soap and warm water [20]. Topical antibiotics, such as mupirocin, three times daily for three-to-five days is recommended for mild to moderate cases of impetigo [23,34]. Contrastingly, oral antibiotics, such as dicloxacillin or cephalexin, 250-500 mg four times daily should be used in severe cases of impetigo [23,34]. If MRSA is suspected, use clindamycin 300-450 mg daily or TMP-SMX one-to-two double-strength tablets two times per day [10,34].

Erysipelas:Conservative treatment of erysipelas includes hydration, elevation, diuretics, and compression stockings. For mild to moderate cases, oral penicillin 500 mg every six hours or amoxicillin 500 mg every eight hours should be used [10]. In severe cases (fever and chills are present), intravenous ceftriaxone 1g/24 hours is recommended for five-14 days or until resolution [10]. Treatment is recommended for five-14 days, though most infections show improvement in 24-72 hours [10].

Cellulitis: Cellulitis treatment is multifactorial and requires treating underlying conditions and supportive medical care. Elevation of the affected area is indicated to allow for lymphatic drainage [10]. Mild cellulitis can be treated with oral antibiotics such as dicloxacillin or cephalexin 500 mg every six hours or clindamycin 300-450 mg every six-to-eight hours [10]. Severe cellulitis should be treated with parenteral antibiotics such as cefazolin 1-2 g every eight hours, oxacillin or nafcillin 2 g every four hours, or clindamycin 600-900 mg every eight hours [10]. Treatment for uncomplicated cellulitis typically lasts five-to-six days though improvement should be seen in 24-72 hours [10]. Antibiotic therapy can be extended up to 14 days if the infection is severe or shows a slow response to therapy [10]. Recurrent infections are common and should be treated the same as the original infection. Prevention should involve treating underlying conditions [10].

Abscesses: Abscesses are treated with incision and drainage. The wound can be packed or left exposed to air, as both approaches yielded similar outcomes in wound healing [30]. Antibiotics are also indicated if the disease is severe and widespread with signs of surrounding cellulitis or systemic illness [10]. In this case, clindamycin 450 mg three times daily, TMP-SMX one-to-two double-strength oral tablets two times daily, and tetracyclines (doxycycline 100 mg two times daily or minocycline 200 mg orally once, then 100 mg, two times daily) are recommended [10]. Patients are treated for five-14 days, depending on severity and response to therapy [10].

NF: NF is a medical emergency. Early identification is crucial to reducing amputation and mortality rates [32]. Treatment should involve consultation with surgeons, internists, and infectious disease specialists [32]. Patients are often isolated to limit the spread to medical personnel [11,32]. Broad-spectrum antibiotics are often used to cover aerobic and anaerobic bacteria such as *Escherichia* *coli* (E. coli), Bacteroides species, group A streptococcus, and Peptostreptococcus species [33,35]. Early and aggressive drainage and debridement are often critical for patient survival [33,35].

Treatment Considerations for Deployed Service Members 

The susceptibility to infections is higher in operational settings due to communal living and close contact with others [10]. Due to the contagious nature of bacterial infections, proper personal hygiene practices and early lesions evaluations are imperative in military settings [11]. Recurrences are common due to the challenges associated with environmental modifications, particularly in training. Bacterial infections are typically treated with topical antibiotics. Although orally administered, antibiotics serve as a feasible option for a deployment setting [11,19]. Due to limited laboratory access, utilize broad-spectrum antibiotics including linezolid, meropenem, and clindamycin. Table 1 summarizes the diagnostic criteria and management recommendations for an operational setting.

**Table 1:** Bacterial soft tissue infections: diagnostic criteria and management considerations for deployment SSTI: Skin and soft tissue infections Source: [10-35]

Conditions	Diagnostic Criteria	Management Consideration for Deployment
Bacterial folliculitis	Physical exam findings include follicular pustules, inflamed follicular papules, and pruritic rash. To confirm the diagnosis, a skin culture with gram stain can be performed.	Hygiene practices are essential to prevent the spreading and worsening of bacterial folliculitis. Soldiers must clean infected areas with warm water and antibacterial soap. Topical mupirocin can be used for treatment when limited skin is involved. If the infection involves an extensive area of the skin, oral antibiotics are warranted.
Bullous impetigo	Physical exam findings include flaccid and fluid-filled bullae that have a crusty brownish appearance when ruptured. These lesions are often located on the trunk.	Use disinfectant soaps to clean the area. Topical antibiotics such as mupirocin 2% cream can be applied daily for 7-10 days.
Non-bullous impetigo	Physical exam findings include papules, vesicles, pustules, and golden adherent crusts involving the face and extremities.	
Erysipelas	Physical exam findings include sharply demarcated and raised unilateral erythema. Also may present with burning, tenderness pruritus, and fever.	Mild SSTIs are locally confined and can be treated with oral antibiotics such as penicillin VK, cephalexin, and clindamycin.Moderate SSTIs present with systemic symptoms and can be treated with IV antibiotics, penicillin, cefazolin, ceftriaxone, and clindamycin. Severe SSTIs can present with sepsis and may require surgical debridement, culture, and IV antibiotics such as vancomycin, piperacillin/tazobactam, and meropenem.
Cellulitis	Physical exam findings include warmth, erythema, edema, tenderness, and fever.	

Leishmaniasis

Leishmaniasis is a vector-borne, parasitic infection caused by a heterogeneous group of protozoans of the Leishmania genus [36]. Leishmaniasis is divided into three groups: cutaneous leishmaniasis (CL), visceral leishmaniasis (VL), and mucocutaneous leishmaniasis (MCL). CL is typically divided geographically into two forms: Old World CL and New World CL [36]. Old World CL is transmitted by phlebotomine sandflies, which are endemic in the eastern hemisphere. The etiologic agents of Old World CL include *Leishmania tropica, L. major, L. aethiopica, L. infantum*, and *L. donovani*. The New World CL is transmitted by flies of the genus lutzomyia and is endemic in the western hemisphere. The etiologic agents of the New World CL are either in the *L. mexicana* species complex (*L. mexicana*,* L. amazonensis*, and* L. venezuelensis*) or the subgenus Viannia (*L. (V.) braziliensis, L. (V.) guyanensis, L. (V.) panamensis, and L. (V.) peruviana*) [36]. *L. infantum *also causes CL in the New World [36].

Leishmaniasis is endemic in more than 98 countries, particularly in the Mediterranean region, Africa, India, Southwest, and Central Asia, and South and Central America [36,37]. It is estimated that the worldwide incidence of VL is 50,000-90,000 infections per year [36]. Contrastingly, the worldwide incidence of CL is 600,000-1,000,000 infections per year [36]. Understanding the epidemiological data of leishmaniasis is essential to military readiness as it prepares healthcare providers for outbreaks in deployed settings. 

From 2001 to 2016, the incidence of Leishmaniasis among US military personnel (active and reserve) was 7.2 cases per 100,000 person-years. However, due to the increased use of personal protective equipment (PPE) by military personnel, the incidence of leishmaniasis has declined significantly in recent years [38]. Most cases of leishmaniasis in deployed settings were CL (58.1%), with *L. major* being the most common subtype [39,40]. 

During the 2001-2006 surveillance period, 86.6% of total leishmaniasis cases were acquired in the Middle East. Most cases were acquired in Iraq (60.5%), followed by Kuwait (31.8%) and Afghanistan (7.4%) [39]. Other countries with higher incidence rates include Brazil, Iran, Peru, Saudi Arabia, and Syria [41,42].

**Figure 3:**
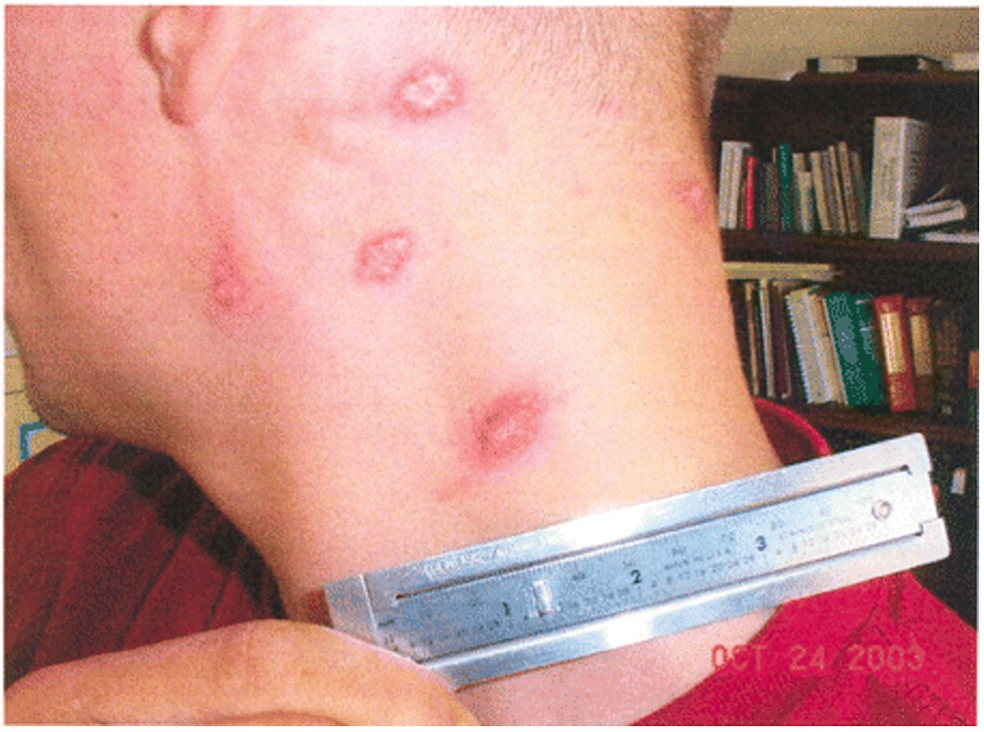
CL Painless, mildly pruritic, indurated lesions the with raised borders and a central scale [52] CL: Cutaneous leishmaniasis

Clinical symptomatology of leishmaniasis is contingent on the pattern of the disease, ranging from self-limiting cutaneous lesions (seen in CL) to end-organ damage (seen in VL) [39,40]. Fortunately, most cases encountered in the US military are related to CL. Clinical symptoms begin with solitary or multiple erythematous macules or papules surrounding the sandfly bite [39,40]. The lesions enlarge and develop central ulceration (Figure 3). The lesions are generally painless with varying degrees of discomfort. In New World CL, the ulcer typically presents with thick, white-yellow fibrinous material [39,40]. Contrastingly, the ulcers seen with Old World CL are often covered with hyperkeratotic eschar. The New World CL can disseminate from the skin to the naso-oropharyngeal mucosa leading to mucosal leishmaniasis [43]. Mucosal leishmaniasis presents as mucosal bleeding and nasal blockage. These mucocutaneous ulcers can impact swallowing and cause difficulty breathing in severe cases [44]. 

The incubation period of CL can range from weeks to months [44]. This long incubation period presents a challenge for healthcare providers to identify infected individuals promptly. In addition, soldiers contract the infection in an endemic area and become reservoir hosts, delaying the diagnosis, and impacting military readiness [44]. Therefore, accurate diagnosis and rapid treatment of infected soldiers are vital to prevent the progression and severity of the disease.

Standard of Treatment

Proper tools for diagnosing leishmaniasis are essential for military physicians. In 2014, the US Food and Drug Administration (FDA) approved CL Detect^TM^ Rapid Test to diagnose CL [45]. The CL Detect^TM^ Rapid Test has 96% sensitivity in the non-endemic population, and this test has sensitivity and specificity of 100% and 84%, respectively, in the endemic region [45]. This detection kit is used for ulcerative skin lesions and should not be used to test human serum. Likewise, the kit is not used to diagnose VL or MCL [45].

CL is difficult to prevent in endemic areas. At this time, no approved prophylactic treatments exist for CL [46]. The most effective preventative method for CL is using PPE to avoid the bite of infected sandflies, such as pyrethrin-treated bed nets, permethrin-impregnated uniforms, and insect repellents [47]. Despite protective measures, CL cases among deployed military personnel continue to be reported [48]. Intravenous sodium stibogluconate (SSG) at a dosage of 20 mg per kg for 20 days is the mainstay treatment for CL [39]. However, SSG can cause adverse effects, including fatigue, arthralgia, myalgia, headaches, and chemical pancreatitis [49].

Additionally, amphotericin B has two formulations used to treat CL: amphotericin B deoxycholate and liposomal amphotericin B [48,49]. Treatment of CL with amphotericin B deoxycholate requires hours of IV infusion daily for 20 to 30 days [48]. The long duration of treatment puts patients at risk of developing irreversible nephrotoxicity, anemia, hypokalemia, fever, and peripheral vein phlebitis. Therefore, amphotericin B deoxycholate is not recommended as a first-line treatment for CL [48]. Liposomal amphotericin B has fewer adverse effects, and it is recommended to treat CL and reduce the risk of developing MCL [49]. However, liposomal amphotericin B has provided lower efficacy in comparison to amphotericin B deoxycholate [50,51]. Additionally, pentamidine has been used for the treatment of CL [50,51]. However, data regarding its efficacy are limited. Pentamidine has numerous side effects and is generally a last resort treatment for *L. (V.) guyanensis *infection acquired in the Guyanas [50,51]. 

Patients infected with CL should be monitored for a minimum of six-12 months. Signs of healing include flattening of the skin lesion, reduction of the size of the lesion (by more than 50%) within four-to-six weeks of treatment, re-epithelization of ulcerative lesions, and no formation of new lesions [51]. Complete healing of skin lesions usually takes three months following treatment. Incomplete healing (or failure to respond to therapy) by the end of the three months warrants additional treatment (with the same or alternative agent). Ulcerative lesions are open wounds, and therefore superimposition of bacterial infection can develop [51]. Continuous wound debridement and cleaning are necessary to prevent complications. Applying a thin layer of petroleum-based ointment to lesions after bathing is recommended [49].

Fungal infections

**Figure 4:**
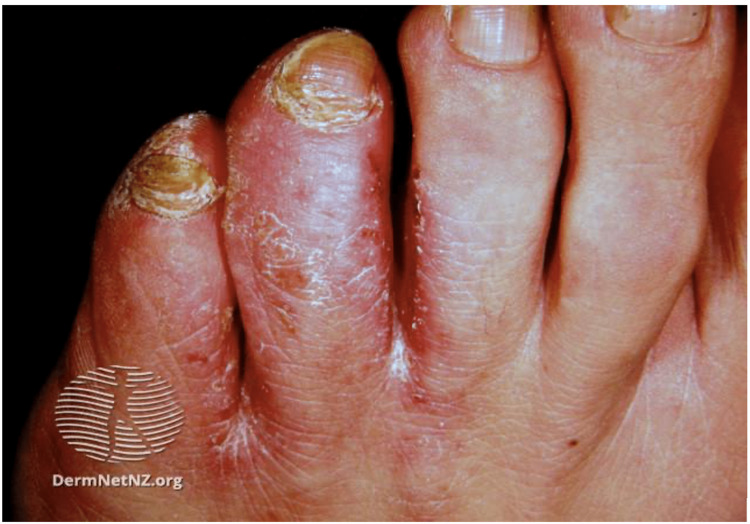
Tinea pedis Scaled and inflamed skin in the interdigital space between the fourth and fifth toes Source: DermNet NZ

Fungal infections of the skin are caused by dermatophytes. The incidence of fungal infections in the US military is 13.5 per 100 military conscripts [53]. While other fungal infections occur in active-duty personnel, tinea pedis, caused by *Trichophyton rubrum*, is the most common [8,16]. Tinea pedis, or athlete’s foot, is a chronic fungal infection that affects the toenails and digital web spaces (Figure 4) [8,16]. Diagnosis is made clinically and is confirmed by potassium hydroxide (KOH) prep [11]. A positive sample shows refractile, long, smooth, undulating, branching, and septate hyphal filaments [54]. 

Clinical presentation of tinea pedis involves erythema, maceration, and painful vesicles between the toes, though the lateral and plantar surfaces can be affected [55]. A striking 10.4% of all military dermatological consultations are due to tinea pedis [2]. Though mortality and morbidity are low, superficial fungal infections can incapacitate soldiers and must be treated promptly [2]. Risk factors for dermatophyte infections include trauma, occlusive footwear, hot and humid climates, and communal living [56]. Improved hygiene, synthetic moisture-wicking socks, and complete foot drying before putting on shoes are essential ways to reduce the risk of tinea pedis [54,56].

Standard of Care 

Patients with chronic tinea pedis benefit from proper foot hygiene. This includes wearing wide shoes, drying between toes after bathing, and changing shoes and socks frequently [54,57]. Patients should not walk barefoot, share clothes, or share shoes to prevent the spread of this condition [54,57]. Additionally, education about adherence to treatment regimens and the risk of reinfection should be emphasized to service members. 

Tinea pedis typically responds well to topical antifungal agents. These medications include butenafine or terbinafine creams once or twice daily for one-to-four weeks [11,54,57]. Oral antifungal treatments are indicated if the disease is extensive, severe, resistant to topical treatments, or if the patient is immunocompromised. In this case, oral terbinafine 250 mg once daily should be used for four weeks [54,57]. Other oral treatments include Itraconazole, Fluconazole, and Griseofulvin. The goal for treatment should be complete symptom resolution [57].

Treatment Considerations for Deployed Service Members

Timely treatment of fungal infections promotes military readiness. Hot and humid climates breed higher rates of fungal infections. As such, deployment locations in the Middle East, East Asia, and other tropical locations have an increased rate of fungal infections [2]. In an operational setting, foot powder composed of 83% talc, 10% starch, and 3% salicylic acid (SA) offers symptomatic relief [57]. Topical miconazole nitrate cream can be added to the treatment. It is crucial for soldiers to limit barefoot walking and likewise wear shower shoes to prevent the spread of infection. It is helpful to ensure feet are completely dry after bathing and before putting on socks and shoes. Additionally, wide shoes improve breathability for feet. Frequent changing of shoes and socks also aids in infection control [54]. 

Treatments suited for an operational setting are similar to civilian-based treatments. However, topical administration can be more difficult for active-duty service members. Oral treatments are still used in severe cases or in patients whose infections have spread to other body areas [58]. Table 2 summarizes the diagnostic criteria and management recommendations for fungal infections in deployed settings.

 

**Table 2:** Fungal and parasitic infections: diagnostic criteria and management considerations for deployment Source:  [36-58] KOH: Potassium hydroxide

Conditions	Organisms	Diagnostic Criteria	Prevention and Management
Leishmaniasis	*L. major, L. infantum, L. donovani, and L. mexicana. *	History and presentation: At least one chronic skin lesion, exposure in an endemic area, and parasite in the specimen.	Management includes teledermatology consultation If needed, refer for treatment with stibogluconate at Walter Reed National Military Medical Center, Washington, D.C., USA.
Dermatophytes	T. capitis, T. corporis, T. cruris, T. pedis, and T. unguium	Physical examination findings include erythematous scaly plaques and pruritic rash. Diagnostic testing (such KOH test) can be performed to confirm the diagnosis.	Prevention includes frequent attire changes and limiting occlusive clothing. Treatment includes topical antifungal application and oral antifungal therapy in severe cases.The following antifungal medications can be found in oral and topical forms: terbinafine, itraconazole, fluconazole, and griseofulvin,

Skin cancer

Skin cancer, the growth of abnormal cells in the epidermis, is the most common type of cancer in the human population [59]. Typical diagnostic techniques for skin cancer include physical exams and biopsy of clinically suspicious lesions [59]. Full-body skin examinations should be done in conjunction with the use of non-invasive optical technologies. Such technologies include optical coherence tomography or dermoscopy, which allow physicians to image the skin and view lesion borders, skin cell organization, and thickened layers [60]. Dermoscopy is a type of physical exam helpful in identifying suspicious pigmented lesions using ABCDE criteria from the American Academy of Dermatology (AAD), whrein A stands for asymmetry (one half of the lesion does not match the other), B for border (irregular, scalloped, poorly defined, notched, or blurred), C for color (uneven pigment can include a shade of brown or black, patches of pink, red, white, or blue). D for diameter (lesions > 6mm warrant prompt evaluation), and E for evolving (rapid change in size, shape, or color) [61].

Biopsies include shave biopsy, punch biopsy, wedge biopsy, or excisional biopsy. While biopsies can provide a definitive diagnosis, it can be difficult to obtain biopsies in military operational settings [1].

Skin Cancer Screening

Screening for precursor lesions helps mitigate the risk of developing skin cancer. Precursor lesions can progress into cancer if left untreated [59]. 

**Figure 5:**
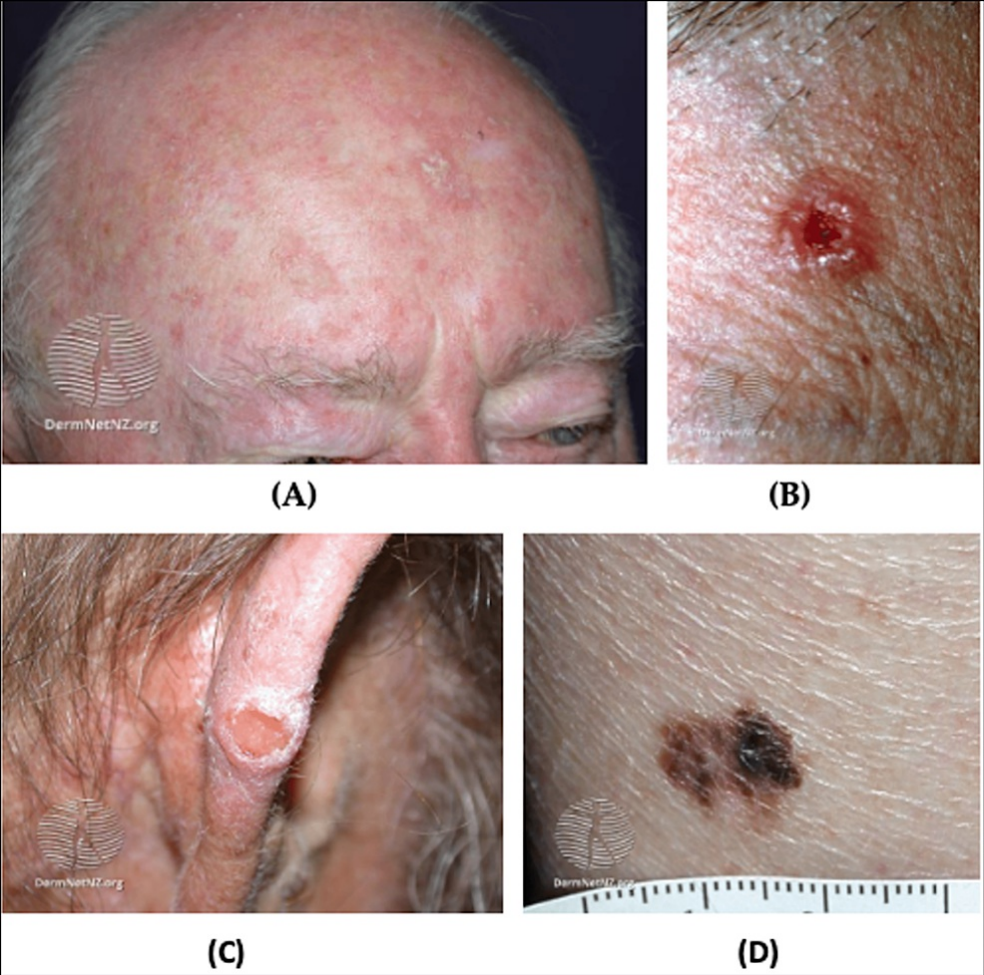
AK and cutaneous cancer (A) Facial AK, multiple small erythematous scaly plaques with hyperkeratosis; (B) BCC, nodulocystic, a solitary ulcerated lesion with an irregular, raised pearly pink border and telangiectasia; (C) Cutaneous SCC; (D) Superficial melanoma, hyperpigmented lesion with an asymmetric and an irregular border Source: DermNet NZ AK: Actinic keratosis; BCC: Basal cell carcinoma; SCC: Squamous cell carcinoma

Actinic keratoses (AKs) are the most common precancerous skin lesions and are described as rough, scaly papules or plaques (Figure 5A). AKs commonly develop on sun-exposed areas including the hands, face, and scalp of Caucasian adults [59,62]. The cause of most AKs is prolonged ultraviolet (UV) exposure. Approximately 5-20% of AK lesions progress to squamous cell carcinoma (SCC) in 10-25 years [62,63]. AKs with a larger diameter, location near the ears, poor differentiation, and perineural invasion of the primary lesion are more likely to progress to SCC [62,63]. It is prudent to note that AKs typically affect the elderly population and are seen less commonly in the younger military population [62,63].

Keratoacanthoma also referred to as pseudotumor, appears very similar to SCC and can be benign or malignant [64]. These tumors are characterized by a rapid growth onset and symmetrical organization of the lesion around a crater with a spontaneous regression [64].

Cutaneous horns (CH) are a less common precursor lesion to SCC. CH are firm plaques or papules, appearing yellow to white, up to several centimeters in length [65]. These precursor lesions commonly occur on the face, neck, and hands [66]. While 60% are benign, CH must be monitored, and potentially removed, as CH can become cancerous [65].

Non-Melanoma Skin Cancers (NMSCs)

NMSC is far more common than melanoma and generally has a better prognosis [67]. The two most common NMSC types are basal cell carcinoma (BCC) and SCC [59]. BCC typically appears on sun-exposed areas and presents as a smooth, pearly papule, though there are various subtypes (Figure 5B). Similarly, SCCs appear on sun-exposed areas but present as red, hard bumps (Figure 5C). BCCs are more common than SCCs and generally do not metastasize [68].

Melanoma

Melanoma, also known as cutaneous malignant melanoma (CM), has a high mortality rate due to rapid metastasis and recurrence rate. In addition, melanoma is resistant to many available treatments [69]. The incidence rate of melanoma in the US military varies by demographic and occupation. For the general population, melanoma comprises about 4% of all cancer diagnoses but 74% of all cancer mortalities [70]. Skin cancer rates also vary by branch deployment in the US military. A surveillance report from the Defense Medical Surveillance System between 2000 and 2011 found that melanoma cancer incidence (per 100,000 persons) was 10.5 in all military branches, 15.5 in the Air Force, and 8.6 in the Army [71,72]. 

Melanoma often presents on the trunk or legs of an affected person but are known to metastasize to other areas and organs in the body [68]. The three most common subtypes of melanoma are superficial spreading melanoma (SSM), nodular melanoma, and lentigo maligna melanoma (LMM) [73]. SSM is associated with low cumulative solar damage and is characterized by horizontal growth [73,74]. SSM is most commonly found on the trunk for men and the lower extremities for women and are usually multicolored. LMM is associated with high cumulative solar damage and typically occurs on the scalp and/or neck of elderly patients [73,75]. LMM lesions are usually large, black, blue, flat, or slightly raised and have ill-defined borders. Nodular melanoma is associated with high or low cumulative solar damage and typically grows vertically [73,76]. These lesions present as pigmented or non-pigmented and may have a flat component (Figure 5D). The growth type and directionality of the tumor are the most significant factors in determining the aggressiveness of the tumor. Vertical invasion of tissue with rapid growth patterns are indicators of aggressive melanoma with a high potential for metastasis. Metastatic melanoma often spreads to lymph nodes, the liver, lung, brain, and bone [77].

Incidence

The incidence rate of skin cancer is high among deployed service members and is greatly affected by deployment geographical location. For example, of the patients who visited a combat support hospital in Iraq, 8% were diagnosed with NMSC over the eight months period that was sampled in the study [4]. Similarly, 67% of the cases of NMSC found in veterans from World War II were from the Pacific front rather than the European front. By nature of their jobs, active-duty service members in the US military have a greater risk of melanoma and nonmelanoma cancers [70]. The increased incidence of skin cancer among deployed service members is multifaceted. Such factors include limited access to sun-blocking materials such as sunscreen creams and UV protective clothing [71].

Risk and Demographic Factors

The most common risk factor for melanoma and NMSC is prolonged UV exposure. In operational settings, access to UV protective barriers and sunscreen is generally limited [4,71]. When combined with prolonged sun exposure, skin cancer rates significantly rise in active-duty service members. Demographic factors also affect the incidence of skin cancer in a population [68]. Low expression of melanin contributes to the increased incidence of skin cancers among the fair-skinned population [68]. Men are more likely to develop skin cancer and have a poorer prognosis than women [68]. Additionally, skin cancer rate increases with age [68]. Veterans tend to be overwhelmingly male, white, and older than the general population, which contributes to the higher rates of skin cancer in veterans [68].

Prevention

Sunscreen usage is the most crucial preventative measure to consider for melanoma and nonmelanoma cancer in a deployed setting [78]. In a survey done on soldiers from Operations Iraqi Freedom in 2015, it was found that less than 30% of soldiers use sunscreen when deployed [78]. This issue stems from inadequate access and insufficient education as well as a greater focus on immediate concerns for safety rather than the long-term effects of sun exposure [71]. Reducing sun exposure for those who are deployed may come at the cost of combat readiness or mission effectiveness. Therefore, efforts to increase the prevention of skin cancer among deployed service members should include adequate access to sunscreen and protective gear. Likewise, it is valuable to provide educational classes to service members on the risks and preventions of skin cancer in the US military [78]. Using sunscreen regularly is a critical behavioral change that can determine whether a person develops skin cancer [79]. An inherent drawback of sunscreen is that it is inconvenient to reapply during a mission, and reapplication is necessary for maximum efficacy. Additionally, sunscreen is less effective after the skin is submerged in water for a prolonged period of time [71]. In these cases, other options such as clothing with UV protection should be prioritized.

Lastly, oral nicatinamide (Vitamin B3) offers a spectrum of photoprotective effects, including enhanced DNA repair, reduction of UV radiation-induced immunosuppression, and regulation of inflammatory cytokines [80]. Furthermore, nicatinamide modulates skin barrier functions and regulates cellular energy levels post-UV exposure. Nicatinamide may be pharmacologically utilized to reduce AKs [80]. In high-risk individuals, reduction of AKs decrease the incidence of nonmelamona skin cancers, such as SCC [80]. Nicatinamide is a nontoxic and cost-effective preventative measure that can be easily administered in an operational setting [80].

Skin self-examination (SSE) has been utilized successfully in civilian settings to increase early diagnosis rates by identifying precursor lesions, which can lead to a more optimistic prognosis [81]. A meta-analysis of SSE diagnostic test accuracy found that SSE methods can detect suspicious lesions with 59% sensitivity and 82% specificity [81]. Additionally, smartphone applications for identifying skin lesions may help increase soldiers’ awareness of precursor lesions. Applications such as iDoc24(®) and FotoFinder Handyscope(®), a mobile phone dermoscopy, have been used to identify suspicious lesions with moderate to substantial interobserver agreement between the teledermoscopists and the in-person dermatologists [82]. Despite this, other studies have not shown significant improvement in using telehealth apps to identify skin cancers when compared to naked-eye SSE [83]. More research must be done before smartphone apps can be deemed a reasonable solution. It is also important to note that these studies were completed using an iPhone 4 and iPhone 5-8. The advancement of smartphone cameras in the past five years may alter these results. [82,83].

Standard of Treatment

Treatment options for CM, NMSC, and precursor lesions include surgical excision, topical treatments, photodynamic therapy, cryotherapy, and radiation therapy. The treatment choice is determined by the recurrence rate, function preservation, patient expectations and wishes, risk factors, and side effects [84].

BCC:Surgical excision is the recommended treatment for BCC. Removal of BCC lesions must include some unaffected skin as BCC lesions often extend beyond the visible tumor [84]. Electrodesiccation and curettage (ED&C) is recommended if the lesion is in a low-risk location, such as on the trunk or extremities, while Mohs micrographic surgery (MMS) is indicated if the lesion is in a particularly high-risk location [84].

If surgery is contraindicated, cryosurgery, topical therapies (imiquimod, 5-FU), photodynamic therapy, or radiation therapy can be used. It is important to inform patients that non-surgical management of BCC is associated with lower cure-rates. Additional treatment options include radiation therapies: superficial radiation therapy, isotope-based brachytherapy, and external electron beam radiation [84].

Photodynamic therapy involves the application of photosensitizing cream (5-aminolevulinic acid (5-ALA) or mannosylarabitol lipid (MAL)) followed by exposure to light irradiation for one to several hours, which leads to tumor cell death [62]. This treatment option is very effective on its own or in conjunction with other topical treatments [68]. Photodynamic therapy provides better cosmetic outcomes than other options. Side effects of photodynamic therapy include photosensitivity for up to 48 hours, erythema, edema, tenderness, crust, and erosions [84].

Melanoma:Melanoma is best treated with surgical excision. The AAD recommends surgical margins between 1-2 cm, but these recommendations vary based on the depth of the lesion [85]. MMS or staged excision are the preferred treatment modalities for melanoma in-situ (MIS) and lentigo melanoma (LM) type on the face, scalp, or ears. MMS has the highest success rate and provides the best cosmetic outcome [67,84]. Sites such as the head, neck, and acral sites are not currently recommended for surgical modalities. Sentinel lymph node biopsies (SNLB) is recommended for melanoma over 0.8 mm thickness, and clinicians should follow guidelines provided by the AAD [85].

Another treatment option for low-grade melanoma or MIS is topical imiquimod 5% cream alone or in conjunction with tazarotene 0.1% [68,85]. It is pertinent to note that the risk of undertreatment is greater with topical treatments, and metastasis is a risk. Radiation therapy has been shown to be effective if surgery is contraindicated [85].

Treatment Considerations for Deployed Service Members 

The most critical way to reduce the incidence of skin cancer in the military is via prevention. Soldiers should be encouraged to wear sunscreen every day and reapply every two hours when feasible [68,71]. Additional UV protection can be provided with clothing and avoidance of sun exposure. Obtaining biopsy results can be slow and limited in an operational setting. In general, deployed service members with lesions suspicious of NMSC should undergo surgical excision with watchful waiting. However, more advanced treatments are required if larger lesions are present. In such cases, service members can be medically discharged back to the US for more comprehensive treatment [68].

Surgical intervention can lead to infection. Due to limited access to dermatological surgeons, treatment with a surgical or superficial ablative method is challenging to perform in an operational setting. If the patient is receiving dermatological care primarily over telemedicine, other treatment options might be available. Such treatments include topical phytochemicals. In early stages of superficial BCCs, imiquimod and liquid nitrogen have been shown to be efficacious in eradicating BCC [68]. Regardless of the treatment method, the whole tumor must be removed or treated to prevent relapse [71]. Table 3 summarizes the diagnostic criteria and management recommendations for skin cancer in deployed settings.

**Table 3:** Skin cancer diagnostic criteria and management recommendations for deployment Source: [59-68] AK: Actinic keratosis; BCC: Basal cell carcinoma; SCC: Squamous cell carcinoma

Conditions		Management Considerations for Deployment	Management Considerations for Deployment
Pre-cancerous lesions	Actinic Keratosis	Physical exam findings include non-erythematous macular lesions with scaling features. A skin biopsy can be performed to confirm the diagnosis.	Preventive measures include the application of sunscreen, avoidance of UV exposure at peak sunlight hours, self-skin examination, and regular screening.
Skin Cancer	BCC	Physical exam findings include a lack of pigment network, arborizing vessels, blue-gray ovoid nests, and ulceration. Dermoscopy examination and skin biopsy can be used for definitive diagnosis.	
	SCC	Physical exam findings include dotted and/or glomerular vessels, white to yellowish surface scales, or red-yellowish background color. Dermoscopy examination and skin biopsy can be used for definitive diagnosis.	
	Melanoma	ABCDE diagnostic criteria: A = Asymmetry (one half of the lesion does not match the other), B = Border (irregular, scalloped, poorly defined, notched, or blurred), C = Color (uneven pigment can include a shade of brown or black, patches of pink, red, white, or blue), D = Diameter (lesions >6mm warrant prompt evaluation), E = Evolving (rapid change in size, shape, or color).	

Urticaria 

Urticaria is defined as transient pruritic wheals with or without angioedema [86]. Urticaria affects about 20% of people in their lifetimes, and the civilian emergency room incidence of urticaria is 2 to 73 per 100,000 patients [87,88]. The pathogenesis is thought to involve mast cell and basophil activation with the release of histamine and other inflammatory mediators [88,89].

**Figure 6:**
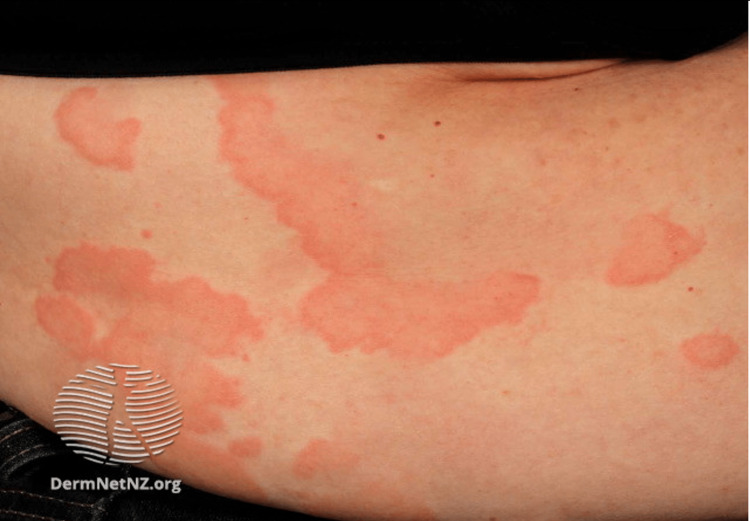
Urticaria Multiple well-defined erythematous, partially confluent lesions with raised serpiginous borders and central clearing Source: DermNet NZ

Urticaria is classified as either acute or chronic. Acute urticaria is more common than chronic urticaria and often associated with upper respiratory infection (40%), drug reaction (9.2%), and reaction to food (0.9%) [87,89]. Chronic urticaria occurs spontaneously or in response to a stimulus, and approximately one-third of cases are autoimmune-related [86,87]. Other triggers for chronic urticaria include chronic infections, shear forces, cold, local pressure, heat, UV or visual light, contact with water, increase in core temperature, or allergen exposure [88]. This subtype primarily affects middle-aged women. The clinical presentation of urticaria involves pruritic, erythematous dermal swelling that blanches when pressure is applied to the affected area. Generalized swelling and burning over the affected area can also be present [87]. This condition can appear on any part of the skin, spread, and coalesce (Figure 6). Urticaria can be pruritic, which can significantly affect patients' quality of life (QOL). Due to the similarity to dermatological symptoms of anaphylaxis, it is critical to rule out this diagnosis. Differentiation is made based on respiratory (wheezing, stridor), cardiovascular (tachycardia, hypotension), and gastrointestinal (diarrhea, vomiting) symptoms present in anaphylaxis that are not present in urticaria [88]. Urticaria can be especially prevalent in military situations as it is difficult for service members to avoid inciting provocations [90]. Responses can develop following sensitization with an allergen. Risk factors for urticaria include stress, drug usage, existing infections, and food sensitivities [87]. Patients should avoid aspirin, ethanol alcohol (EtOH), nonsteroidal anti-inflammatory drugs (NSAIDs), and tight clothing due to their potential to worsen symptoms.

Standard of Treatment

Acute urticaria is often self-limited and resolves on its own. However, if treatment is needed, first-generation H1 antihistamines (diphenhydramine, hydroxyzine, chlorpheniramine, and cyproheptadine) are recommended as fast-acting treatments [88,91]. Potential side effects include sedation, confusion, dizziness, lack of ability to concentrate, and decreased coordination. To avoid these sedative effects, second-generation H1 antagonists (loratadine, desloratadine, fexofenadine, cetirizine, and levocetirizine) can be used [92]. These medications can be titrated up to four times the standard dose to reduce symptoms. If these are not sufficient to control symptoms, H2 antihistamines such as cimetidine, famotidine, and ranitidine are used in treatment [88]. In very severe cases, corticosteroids (prednisone or prednisolone) serve as treatment options [92]. In case of anaphylaxis or severe angioedema that threatens the airway, epinephrine autoinjectors should be used [88]. 

Like acute urticaria, the first line of defense for chronic urticaria is second-generation H1 antihistamines [86]. If symptoms are insufficiently controlled, titration up to four times the standard dose, adding a different second-generation H1 antihistamine, adding an H2 antihistamine, adding a first-generation H1 antihistamine at night, adding a leukotriene receptor antagonist, or any combination of the above should be tried [86,88]. If symptoms are still not controlled, a high-potency antihistamine such as hydroxyzine or doxepin can be employed [91]. Severe cases require a 3-10 day course of oral corticosteroids similar to acute urticaria [88]. Treatments should be stepped back down to the second generation H1 antihistamines before discontinuation, as acute elimination of more intense treatments is not recommended [88].

Treatment Considerations for Deployed Service Members 

Occupational urticaria is well recognized among military service members. The intensity of military training and exposure to various allergenic agents during deployment increase the susceptibility to occupational urticaria. Avoidance of allergens and other triggers can be difficult in deployed settings. Exposure to cold, sun, water, increased body core temperature, and other environmental factors are intrinsic to mission completion. Recommendations should involve avoiding the trigger to the best of one’s ability and the prompt application of the above treatments. Latex is a common cause of urticaria among military service members. Latex can be found in many products, especially gloves. It can cause urticaria by direct contact with the skin or become airborne [90]. 

Service members are repeatedly exposed to allergens, harmful chemicals, and extreme weather. As such, they are likely to develop chronic urticaria, which requires frequent treatment. Dermatological treatments for chronic urticaria mirror the civilian standard of care for chronic urticaria [90]. Additionally, it is beneficial for service members to wear gloves, glove liners, and apply hand cream after work. Table 4 summarizes the diagnostic criteria and management recommendations for urticaria in deployed settings. 

**Table 4:** Dermatitis diagnostic criteria and management recommendations for deployment Source:  [93-119] AD: Atopic dermatitis; UV: Ultraviolet; CD: Contact dermatitis; ACD: Allergic contact dermatitis; TCS: Topical corticosteroids

Conditions		Diagnostic Crtieria	Management Consideration for Deployment
Urticaria		Physical examination includes raised, erythematous plaques with rounded/irregular lesions and central clearing. Allergy testing can be performed to identify the allergen.	Preventive measures include the removal of offending triggers and avoidance of extreme temperatures. Antihistamines medications (preferably second-generation antihistamines) can be used. If anaphylaxis is observed, epinephrine injection must be given immediately.
Eczematous Conditions	AD	The following are required for diagnosis: Pruritus, eczema (acute, subacute, or chronic), typical morphology, and chronic or relapsing course. Additional findings include early age of onset, atopy (personal and/or family history), and xerosis.	Nonpharmacological therapies include avoidance of flares, maintenance of skin hydration, and stress management. Pharmacological treatment is based on the severity of AD. For mild-to-moderate AD, consider topical steroids or topical calcineurin inhibitors. For moderate-to-severe AD, consider increasing the potency of topical agents, phototherapy (UV light), or noncorticosteroid systemic immunomodulatory medications.
	CD	Characteristics of lesions include intensely pruritic erythematous papules, vesicles with serous oozing, and distinct borders that correspond to the site and extent of exposure. A patch test can be performed to confirm the diagnosis.	Avoidance of exposure to allergens is the mainstay of management for ACD. Additionally, symptomatic therapy such as cool compresses, emollients, and wet dressing can be used. TCS can be used for localized dermatitis. If >20% of the body surface is affected, consider systemic steroids).

Other common issues faced when treating dermatological conditions are limited access to medical facilities such as a lack of refrigeration. Medications such as methotrexate and biologic immunomodulatory drugs should remain cold. Furthermore, medications, such as minocycline and isotretinoin, have a wide range of side effects, limiting safe use in a deployed setting [66,93]. 

Contact Dermatitis (CD) and Irritant Contact Dermatitis (ICD)

CD is an inflammatory reaction to irritants or allergens coming into contact with the skin [94]. It is divided into allergic contact dermatitis (ACD) and ICD [95]. The foremost consideration for diagnosis of CD is patient history, including the frequency and duration of recent irritant contacts and ongoing exposures through occupation, hobbies, or medications [96-98]. Any similar reactions in the patient’s history should also be examined. Cases of anaphylaxis are frequently reported for ACD [96]. 

Differentiating between ACD and ICD requires patch testing. This test is intended to produce the elicitation phase of ACD to identify the specific antigen [95,99]. The test involves applying a baseline series of common allergens in diluted forms to the patient’s back using Finn chambers and then removing those patches 48 hours later. The results are then read 48-72 hours post-removal by grading the reactions based on the International Contact Dermatitis Research Group guidelines as negative (-), irritant reaction (IR), equivocal/uncertain (+/-), weak positive (+), strong positive (++), or extreme reaction (+++) [99].

Patch testing should not be completed if the patient has an active rash. Patch testing provides information on other triggering substances that patients should avoid in the future [94]. Patients must be informed that rare cases of anaphylaxis have been seen with patch testing [99].

The clinical presentation of CD can vary. Lesions include vesicles, bullae, papules, scaling, fissures, and skin necrosis. The most common location for CD to develop is the hands [95,96]. The onset of ACD is typically acute, leading to affected areas with ill-defined borders where the reaction extends beyond the area of contact [94]. The affected area is likely to cause pruritus, erythema, edema, bullae, and vesicles. Contrastingly, ICD typically has a more gradual onset, requiring multiple exposures to the triggering agent. The rash on the affected areas usually has well-defined borders [94]. The affected area is likely to be painful, sting, and burn [97]. Patients also present with erythema, desquamation, fissures, and rash [96].

ICD and ACD have different cellular mechanisms for reactions. ACD is a type IV hypersensitivity reaction when T cells respond to an exogenous antigens [100]. This interaction creates an innate immune response in a sensitized patient [96]. The sensitization period is not clinically noticeable and involves reproducing allergen-sensitive T cells, which eventually lead to the clinical presentation during the elicitation phase [99]. The sensitization phase does not occur if the patient has already elicited a reaction to a chemically similar substance [99]. It is also common for light to enhance the reaction to an allergen, a phenomenon referred to as the photo CD [96]. Common allergies that lead to ACD include latex, pesticides, nickel, chromium, fragrances, neomycin, formaldehyde, cobalt, balsam of Peru, and parthenium [96,100]. Many of these allergens are present in clothing, accessories, drugs, insect repellents, and antiperspirants [101]. Additionally, 50-70% of the US population suffers from a poison ivy allergy, making poison ivy the most common cause of ACD [96]. 

ICD can stem from many exposures, including chemical, mechanical, and environmental. ICD occurs when toxins such as chemicals or metal ions induce a T cell response in epithelial cells [96]. This response leads to inflammation from cytokines that cause skin barrier and epidermal disruption. Most chemical contacts have the potential to develop into ICD [96]. Common causes of ICD are skin exposure to liquid that lasts more than two hours, wet work and wearing gloves for two hours or more, and washing hands frequently. Chemical irritants have differing toxic and degenerative properties that are determined based on the concentration, duration of the exposure, and presence of other chemical irritants [94]. ICD can also stem from UV radiation, extreme temperatures, and low humidity [94]. Mechanical mechanisms include friction, occlusion, pressure, and vibration [97]. The various causes of ICD are more detrimental when combined together [96]. For example, the friction from wearing gloves can be exacerbated by sweating on a hot day. Such factors are particularly relevant for active-duty service members due to the nature of their work.

Many common irritants and allergens affect deployed service members. However, certain exposures are more common [8]. Irritant exposures specific to this population include beetles, caterpillars, agave, and N, N-Diethyl-meta-Toluamide (DEET) from insect repellent. Unique allergen exposures include various oils, industrial fluids, vaccines, corticosteroids, preservatives, dyes, and detergents [10]. Additionally, military uniforms are made with formaldehyde resins, dyes, chromate, and other chemicals frequently associated with ACD [98]. Moreover, military camouflage face paint contains castor oil derivative, which is a known trigger for ACD [102]. Metal composites such as zinc and manganese in shrapnel also commonly lead to ACD [98,102].

CD accounts for 15-20% of reported occupational diseases [100]. Among civilians, 136 in over 10,000 individuals are diagnosed with CD [94]. In military personnel, the incidence is 9.6-32% and is the fourth most common cause for dermatology consults in the military [101]. In the military and civilian population, 80% of CD cases are ICD, while 20% are ACD [94]. The incidence of ACD risk is determined by a patient’s predisposition to allergic reactions, while ICD is affected more significantly by occupation [96]. Susceptibility to ICD varies with age, sex, or a history of eczema. Older and female patients with a history of AD are more susceptible to CD. In addition, susceptible sites to ICD include damaged, thin, and dry skin with atopic tendencies [96].

Standard of Treatment

The preeminent consideration for CD includes avoidance of provocative agents [95,103]. If the irritant or allergen is unavoidable, ample treatment options are available. PPE such as gloves with liners and other appropriate clothing can be used to limit exposure to the inciting substance [104,105]. As cool temperatures reduce vesiculation, cool water should be recommended rather than hot for ICD cases related to handwashing [97]. 

Other options for the prevention of ICD include using barrier creams, fabric softener, and high lipid-content emollients [104]. Due to the random nature of allergy triggers, ACD prevention is challenging. However, if the allergen is avoided, repetitive flare-ups of ACD can be prevented [105]. Because 75% of patients with occupational CD develop chronic skin disease, treatment must be thorough and prompt [106]. Treatments vary for ACD and ICD and are dependent on the longevity and severity of symptoms [95-106].

For mild to moderate episodes of ICD or ACD, topical therapies are the first line of defense [106]. Topical therapies include corticosteroid ointments, steroids, lotions, sprays, gels, cool compresses with saline solution, shake lotions such as calamine, colloidal oatmeal baths, and emollients [96,106]. Topical corticosteroids (TCS) should not be used on thin skin to avoid skin atrophy. Emollients are used to repair the skin barrier and increase skin hydration [105]. 

Severe and/or chronic ACD and ICD are treated systemically. Options for systemic treatments include antihistamines, corticosteroids, antibiotics, and other anti-inflammatory or immunological agents. Immunomodulators can be helpful as they do not lead to skin atrophy like TCS [95]. In some cases, radiation therapy or psoralen and ultraviolet A radiation (PUVA) can be used [95].

Following the resolution of lesions, it can take up to 4 months for the epidermis to return to its full barrier function [97]. This should be considered for longer-term treatment and general awareness to prevent recurrent flare-ups after treatment has been successful. Patients should also be told to continue using emollients if they are in an occupation that will likely lead to more flare-ups of CD [97].

Treatment Considerations for Deployed Service Members

Military service members with a history of dermatitis are at increased risk of developing exacerbation of contact and irritant dermatitis during deployment. As such, various prophylactic measures should be taken to avoid and identify causative substances. Personal protective equipment and protective clothing should be encouraged to those at high risk of exacerbation. In soldiers with nickel-induced CD, an iron-on patch or nail polish can be applied to cover the metal tab of jeans [107]. Steroids ointments are preferred among service members over steroid creams. Steroid ointments contain less preservatives and are less likely to cause allergic reaction. It is recommended to soak the affected area with water prior to applying the steroid ointment to enhance its effectiveness [107].

Additionally, service members at higher risk should carry mid- to high-potency topical steroids such as triamcinolone 0.1% (Kenalog, Aristocort) or clobetasol 0.05% (Temovate). Topical steroids have been shown to be effective in the treatment of localized acute ACD [107]. In cases of severe dermatitis with extensive area involvement, systemic steroids should be prescribed. This may require time away from the mission and frequent monitoring of side effects. Five-to-seven days of prednisone, 0.5 to 1 mg per kg daily, is recommended [107]. Dosage should be tapered according to the physician’s discretion after initial improvement on this therapy. The use of antihistamine medications such as diphenhydramine and hydroxyzine is discouraged among service members due to its sedative effects [107]. 

AD

**Figure 7:**
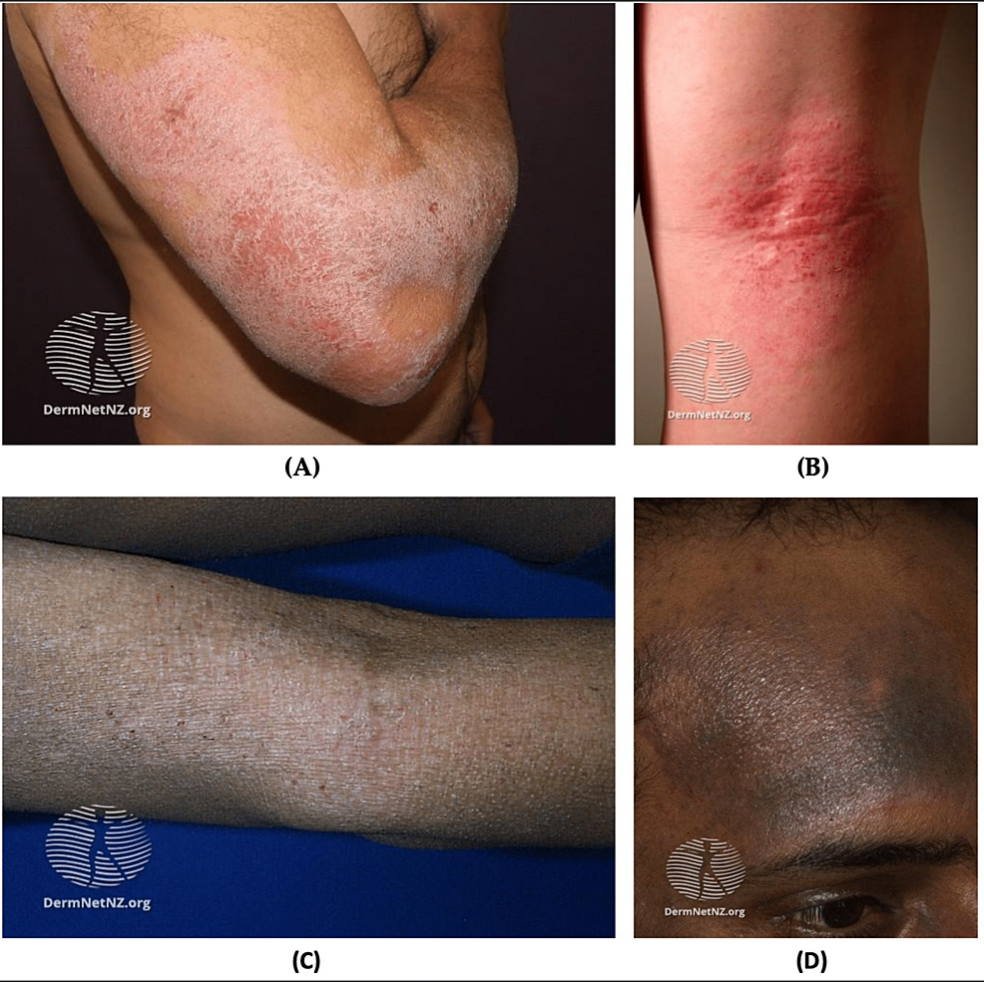
AD (A) Chronic atopic dryness on extensor surface; (B) Acute flexural AD, erythematous macules with excoriations; (C) Lichenification of AD; (D) Hyperpigmentation due to AD in black skin Source: DermNet NZ AD: Atopic dermatitis

AD is chronic relapsing and remitting inflammatory dermatological condition affecting up to 20% of children and up to 7.3% of adults [108,109]. The etiology of AD is multifactorial and involves genetic factors, immunological dysregulation, and exogenous triggers (e.g., dust, mites, heat, dry or humid climate, emotional stress, infections, and skin irritation) [110]. AD presents mostly on flexural and extensor surfaces as scaly, pruritic, and erythematous lesions (Figure 7) [111]. 

AD has been a major health challenge in the military. It comprised approximately 1.7% of all dermatological conditions seen during the Vietnam War, First Gulf War, Peacekeeping in Bosnia and East Timor, and Operation Iraqi Freedom combined [2]. Due to the propensity for recurrent flares, military applicants with a history of AD are often disqualified from joining the military service. US military personnel encounter various emotional and physical stressors during training, deployment, and battlefield [112]. For these reasons, soldiers with a history of AD are more likely to experience recurrent flare-ups of AD. These recurrent flare-ups ultimately compromise soldiers’ health and ability to handle firearms appropriately. As such, these soldiers may require time off to recover, which can jeopardize military mission goals and overall military readiness [112].

AD is diagnosed based on clinical observations. If AD is suspected, immediate treatment should be started. If the patient does not respond to treatment, then the diagnosis of AD should be reassessed [108]. Currently, there is no consensus on specific biomarkers for AD. However, elevated levels of allergen-specific immunoglobulin E (IgE) aid in accurate diagnosis [113]. To increase the accuracy of diagnosis, clinicians should look for various clinical associations and comorbidities, including allergies, asthma, allergic rhinitis, and rhinoconjunctivitis. AD has a consistent association with a family history of atopic diseases and loss of function mutation in the FLG gene. Therefore, clinicians must obtain a thorough history of patients with AD [114].

Patients with AD are at increased risk of viral, bacteria, and fungal infections. Staphylococcus aureus is the most common bacterial superinfection in patients with AD [115]. Patients infected with *S. aureus* are treated with mupirocin 2% cream twice daily for one or two weeks. Oral antibiotics are not recommended for mild skin infection with *S. aureus* but should be reserved for more extensive infection [115].

Additionally, AD patients are at increased risk of co-infection with the herpes simplex virus [116]. AD with concomitant herpes simplex infection is called eczema herpeticum [116]. Patients with this condition should immediately start on acyclovir. The recommended dosage of oral acyclovir for mild cases is 30-60 mg/kg/dl, divided into three daily doses in children and 400 mg three times per day in adults [117]. Patients with severe eczema herpticum or who are immunocompromised should be hospitalized for intravenous acyclovir 5-10 mg/kg every eight hours [118]. Eczema herpticum is a life-threatening condition due to systemic infections and end-organ damage complications. Therefore, it is crucial to start acyclovir promptly after confirming the diagnosis [116].

Fungal or dermatophyte infections are commonly seen in patients with AD [118]. Patients with concomitant fungal infections and AD are treated with topical or oral antifungals in addition to the standard regimen for AD. Treatment for fungal infections is discussed in detail in the fungal infection section [118].

Standard of Treatment

Maintenance therapy of AD depends on the severity of the condition. Very mild AD can be managed with non-pharmacological therapies such as moisturizers, bathing practices, and wet-wrap therapy with or without TCS [114]. It is recommended to limit non-soap cleansers that are neutral to low pH, hypoallergenic, and fragrance-free [114].

For patients with mild-to-moderate AD, consider adding topical pharmacotherapy if non-pharmacological therapies produce an insufficient resolution of symptoms. The preferred treatment is TCS which can be used for adults and pediatrics. The choice of TCS depends on the severity of the condition, the patient’s preference, and age. For mild AD, it is recommended to start with low potency TCS, such as desonide 0.05% and hydrocortisone 2.5% [119]. If low potency TCS is insufficient, escalate to medium- to high-potency TCS such as fluocinolone 0.025%, triamcinolone 0.1%, betamethasone dipropionate 0.05% [119]. Low potency TCSs are applied once or twice per day for two-to-four weeks. Patients with AD are recommended to use emollients either before or after TCS application [119].

Moderate-to-severe AD (with severe functional impairment) is treated aggressively. If AD is refractory to low potency TCS, it is recommended to use the highest potency TCS. However, long-term use of high potency TCS can cause skin atrophy, telangiectasias, folliculitis, and CD [119]. If patients cannot tolerate TCS, topical calcineurin inhibitors can be used as an alternative treatment and maintenance therapy for AD. Additionally, topical calcineurin inhibitors are preferred on the eyelids, neck, and skin folds. Topical calcineurin inhibitors include tacrolimus ointment and pimecrolimus [119]. Tacrolimus comes in 0.1% and 0.03% formulations. The 0.03% can be used for children and adults who cannot tolerate high potency formulation. The 0.1% formulation can be used as initial therapy for adults and those older than 15 years of age [114].

Phototherapy is another treatment option for patients with severe to moderate AD refractory to TCS. Narrowband ultraviolet B (NB-UVB) is the most commonly used phototherapy due to its low-risk profile, efficacy, and availability [120,121]. In addition, phototherapy can be used in combination with other topical treatments.

Management of severe AD can be challenging and require prescribing systemic agents. Most systemic agents are used off-label for the treatment of AD. Such medications include cyclosporine, azathioprine, methotrexate, and mycophenolate mofetil. It is recommended to start with a minimal dosage of these systemic agents [122]. The choice of the systemic agent is highly personalized and depends on many factors that include age, personal preference, response to prior treatments, and immune system status. Systemic steroids are not recommended as a standard of care for AD [122]. However, systemic steroids can be utilized in select cases for short-term bridge therapy to other systemic agents or for severe acute exacerbations [123]. 

Patients with moderate to severe AD to whom TCS and systemic treatments are insufficient and not candidates for phototherapy can be considered for subcutaneous dupilumab [123]. Dupilumab is a human monoclonal antibody that binds to and inhibits the signaling of interleukin (IL) 4. IL-4 is believed to play a major role in atopic diseases [124]. The initial dose is 600 mg for adults, followed by a maintenance dose of 300 mg every other week. Dupilumab has fewer adverse effects than systemic treatments and is a viable option for long-term maintenance therapy [125]. This treatment option has significantly improved the care for service members with severe AD, which would have previously restricted their ability to serve [3].

Patients presenting with acute flares of AD should be treated first with topical pharmacotherapy such as TCS and calcineurin inhibitors [119]. Systemic steroids should be preserved for severe or refractory flares [119]. Additionally, the majority of AD cases present with allergies and other comorbidities such as asthma, conjunctivitis, rhinoconjunctivitis, and eczema. Therefore, it is essential for clinicians to identify these comorbidities and treat them accordingly [110-112]. Furthermore, treatment of these underlying conditions can improve or resolve AD symptoms [126].

Treatment for Deployed Service Members

Treatment of AD in the military remains challenging, particularly in remote deployed settings [3]. While the treatment for AD is relatively straightforward, soldiers with AD face multiple obstacles: length of deployment, lack of refrigeration, lack of access to dermatologists, and weight limits [127]. In addition, soldiers with AD must bring their treatments with them for the length of the deployment. In many circumstances, deployment can last from six months to two years [3]. Thus, soldiers would need to carry a six-month supply of emollients, topical steroids, and systemic medication, which can add up to 20 pounds to their military gear [3].

Additionally, most underdeveloped countries lack electricity and refrigeration, which causes the medication to expire quickly and lose efficacy [127]. These limitations in access to care, medications, and treatment options make AD a difficult condition to treat in the deployed setting [3]. Table 4 summarizes the diagnostic criteria and management recommendations for AD in operational settings. (Table 4) 

Psoriasis 

Psoriasis is a chronic autoimmune inflammatory disease that affects the skin, nails, and joints [128,129]. Psoriasis is diagnosed clinically with body surface area (BSA) and severity of erythema, induration, and scaling [128]. The Psoriasis Area Severity Index (PASI) measures psoriasis in two steps. The first is assessing its severity by assigning scores from 0 (complete lack) to 4 (most severe) for the presence of erythema, induration, and desquamation on each of four regions of the body (head/neck, trunk, upper extremities, and lower extremities) and the second is estimating skin involvement from 0 (no psoriasis) to 6 (90-100%) to estimate BSA in each body region. These two steps are combined using an algorithm to calculate a PASI between 0-72 [130]. PASI scores above 36 are considered very severe and are rare [130].

Psoriasis Subtypes

Psoriasis is classified by age of onset, degree of skin involvement, morphologic pattern, and predominant involvement of specific locations on the body. Early-onset is considered before the age of 40 [123]. Severity is determined on the following scale: <5% BSA (mild), 5-10% BSA (moderate), and >10% BSA (severe). It is estimated that 75% psoriasis cases are considered mild to moderate [130]. Morphologic patterns are used to determine subtypes based on the descriptions below to determine if the psoriasis is plaque, guttate, erythrodermic, pustular, rupioid, or elephantine. Anatomical site is also considered in classification, noting if the lesions occur on the scalp, palmoplantar, genital, nail, or anal areas [123].

Although psoriasis has many subtypes, three key symptoms appear in most variants: skin thickening, erythema, and scaling. Subtypes of psoriasis are divided by types of lesions and locations [131]. Subtypes include plaque, flexural, guttate, pustular, erythrodermic, and nail psoriasis [128]. Patients often present with more than one subtype simultaneously [128]. 

**Figure 8:**
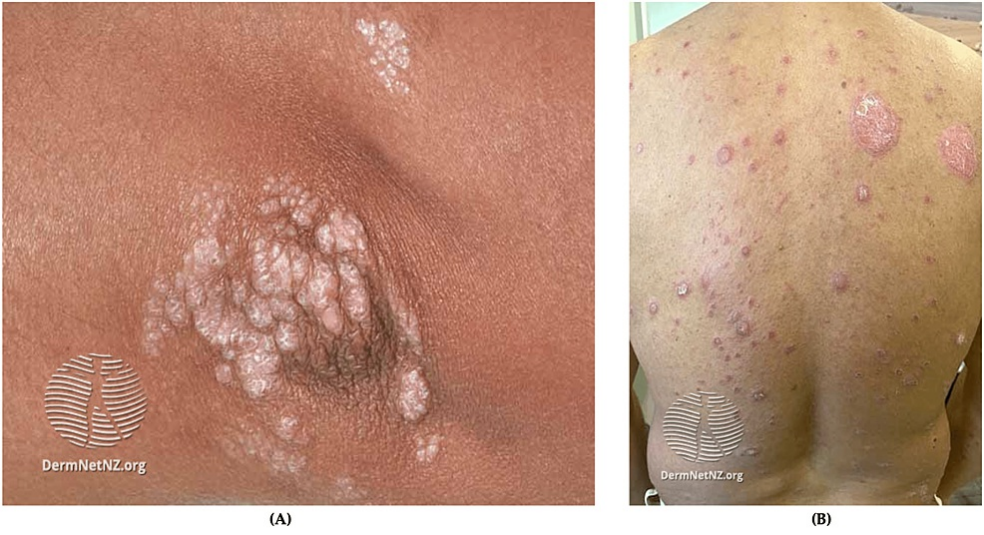
Psoriasis (A) Chronic plaque psoriasis; (B) Plaque psoriasis skin of color Source: DermNet NZ

Plaque psoriasis: The most common subtype is plaque psoriasis or vulgaris, affecting 80-90% of patients with psoriasis [130]. Plaque psoriasis presents erythematous plaques distinguished by silvery or white scale (Figure 8A) [132]. These lesions can range from 1 cm to several cm in size. Plaques can present as a few small lesions or many that cover large portions of the body (Figure 8B) [130]. The lesions are distributed symmetrically and tend to affect knees, elbows, trunk, and scalp. Thickened lesions over palms, soles, and joint lines can lead to fissuring of the skin which can cause debilitating pain [128]. Those affected by plaque psoriasis can experience the Koebner phenomenon (psoriasis at sites of trauma) and the Auspitz sign (disruption of the top layer of lesion leads to pinpoint bleeding) [123,124]. 

Guttate psoriasis:Guttate psoriasis affects about 2% of patients with psoriasis and leads to symmetrical teardrop-shaped salmon-pink papules or plaques with very fine scales [130,131]. These lesions typically appear on the trunk or limbs. Guttate psoriasis is preceded by *S. aureus* infection or upper respiratory infection in 66% of new-onset cases. Guttate psoriasis is likely to later develop into a plaque psoriasis [128,131]. This type of psoriasis is primarily seen in patients younger than 30 as the first manifestation of psoriatic symptoms [133].

Pustular psoriasis: Pustular psoriasis is characterized by sterile pustules with an erythematous base underneath. These lesions can be generalized or limited to either fingers and toes or palms and soles [132]. Local outbreaks can be severely limiting in everyday life, but a generalized outbreak can be life-threatening [128]. Pustular psoriasis is further divided into three subtypes by location: Acrodermatitis Continua of Hallopeau (fingers and toes), Palmoplantar Pustulosis (palms and soles of feet), and generalized (many locations simultaneously). Symptoms include fever, myalgia, nausea, leukocytosis, red and tender skin, and systemic toxicity [130,134]. Patients with generalized pustular psoriasis are very sick and should be hospitalized to be monitored for liver function, hypocalcemia, and hydration [122-124].

Erythrodermic psoriasis: Erythrodermic psoriasis affects 2-3% of patients with psoriasis and is characterized by generalized erythema over the majority of the BSA [131]. Erythrodermic psoriasis is usually caused by poor control of existing psoriasis symptoms but can also result from medication withdrawal, systemic infection, and drug reaction [134]. Associated symptoms include hypothermia, chills, dehydration, fever, malaise, lower leg edema, and congestive heart failure [130]. Rare cases of erythrodermic psoriasis can lead to uncontrolled widespread erythema that can be life-threatening due to hypothermia, infection, acute kidney injury, or cardiac failure complications [128].

Flexural psoriasis: Flexural psoriasis presents as erythema without scaling and typically affects the axillae, abdominal, perineal, intergluteal, submammary, inframammary, and genital areas [128]. These areas tend to have increased moisture, so flexural psoriasis presents with less dryness and scaling than plaque psoriasis [131].

Nail psoriasis: Nail psoriasis is characterized by pitting, onycholysis, or detachment of the proximal portion of the nail from the nail bed [130]. Some patients have oil spots that appear as orange-yellow areas beneath the nail. Other symptoms are thickened, dystrophic, discolored nails, and subungual hyperkeratosis, which occurs when keratinous material collects under the nail plate [134]. It is estimated that 65% of patients with plaque psoriasis have nail psoriasis affecting their fingernails and 30% affecting their toenails [134]. This type of psoriasis is also considered a marker that the patient will likely develop psoriatic arthritis (PsA) in the future [130].

Pathogenesis 

The pathogenesis of psoriasis is poorly understood but known to be caused by genetic and environmental factors, skin barrier disruption, and immune dysfunction [135]. Psoriasis is a largely heritable disorder with a heritability of 60-90%. Heritability increases for those with early-onset psoriasis and those with plaque psoriasis. The genetics of psoriasis is associated with antigen presentation, NF-kappa B signaling, and skin barrier function [136]. Environmental factors associated with psoriasis onset include obesity, smoking, EtOH abuse, and lithium and beta-blocker usage [129,130]. In addition, exposure to air pollutants and excessive sunlight have been shown to trigger psoriasis [129]. Other immune disorders such as Crohn's disease and multiple sclerosis can also lead to the development of psoriasis [130].

Stress is a significant trigger for psoriatic onset or exacerbation in 31-88% of cases [137]. Stress acts on the immune system via activation of the autonomic nervous system (ANS) and subsequent secretion of glucocorticoids which impact the immune system [137]. Additionally, stress can activate dendritic cells and, therefore, modulate the release of neuromodulators and catecholamines, leading to the exacerbation of psoriasis [137]. This is important in a deployed setting because pre-deployment psoriasis flares are common due to psychological and physical stressors [93].

Incidence

In the US, the incidence of psoriasis is 78.9 per 100,000 people [138,139]. Psoriasis is just as common in soldiers as in civilians, making up about 2% of the dermatological consultations in active-duty service members [2]. It has been shown that physical, physiological, and psychological stress exacerbate psoriasis [137]. Soldiers are constantly subjected to stressful situations and as such, those with pre-existing diagnosis of psoriasis are likely to experience recurrent exacerbations. Moreover, the majority of psoriasis treatments can make soldiers ineligible for deployment and lead to evacuation [3]. Severe episodes of psoriasis can cause the evacuation of service members [93]. Between 2008 and 2015, 3,600 US soldiers sought treatment for psoriasis while deployed [93]. It is therefore crucial that psoriasis is managed appropriately for soldiers to return to the operational mission [93].

Psoriasis can affect people of any age or sex without discrimination. It does, however, affect women and those with family history earlier in life [128]. Psoriasis also affects Caucasians at a higher rate (3.6%) than any other race: African Americans (1.9%), Hispanics (1.6%), and others (1.4%) [130]. Patients with obesity, type II diabetes, dyslipidemia, and hypertension are at an increased risk of developing psoriasis. The typical age of onset has a bimodal distribution with a peak from 30-39 and a second peak from 60-69 in men [131]. Women are typically affected 10 years earlier than men [128].

One or more comorbidities are present in 75% of patients with psoriasis [128,130,131,134]. Therefore, patients should be monitored for common comorbid disorders such as cardiovascular conditions, psychiatric disorders, and gastrointestinal diseases. Various risk factors and comorbidities are associated with worsening psoriasis. Such risk factors include, but are not limited to, hypertension, hyperlipidemia, type II diabetes, obesity, metabolic syndrome, myocardial infarction, stroke, and peripheral vascular disease [128,130,131,134]. Up to 60% of patients with psoriasis have concurrent psychiatric disorders such as depression and anxiety [132,135]. These psychiatric conditions contribute largely to the reduced QOL, social stigma, and discrimination associated with psoriasis [132,135]. Commonly comorbid gastrointestinal diseases include irritable bowel syndrome, Crohn's disease, uveitis, and ulcerative colitis [131,134]. 

PsA is another comorbid disorder that affects 30% of people with psoriasis [132]. It occurs more often in those with nail dystrophy and scalp, intergluteal, and perianal psoriasis [128]. PsA is characterized by stiffness, pain, and swelling in joints and can eventually reach the level of joint destruction [132]. Psoriasis typically precedes PsA by about 10 years [132]. The Psoriasis Epidemiology Screening Tool (PEST) is used to screen for PsA [128].

Standard of Treatment

Treatment of psoriasis is generally focused on mitigating acute symptoms [135]. Patients with psoriasis typically have high morbidity and experience many side effects throughout the treatment [134]. Targets include 75-90% improvement on PASI [128]. Topical therapies are the first line of defense to treat psoriasis due to their high efficacy and safety. They are most effective for mild to moderate cases. Corticosteroids treat psoriasis through their anti-inflammatory, antiproliferative, immunosuppressive, and vasoconstrictive effects [131,140]. The potency of the selected steroid varies depending on the area of the body affected, age of the patient, patient preference, and severity of the lesions. Lower potency is typically used for the face, intertriginous areas, and areas with thinner skin susceptible to atrophy, such as forearms. Mid-high potency steroids are recommended for standard treatment in adults, while the highest potency corticosteroids are typically reserved for thick, chronic lesions [140]. Corticosteroids should be applied to the affected area twice daily. Once the lesions are resolved, corticosteroid application should continue at a rate twice per week to reduce the chance of recurrence [131]. Intralesional steroids can also be injected for treatment-resistant lesions or very thick lesions in locations such as the scalp, nails, palms, and soles. In this case, triamcinolone acetonide dosed up to 20mg/mL can be injected every three-to-four weeks, varying by the size of the lesion and affected area [141]. 

Adverse reactions to TCS include skin atrophy, striae, folliculitis, telangiectasia, and purpura [131]. Abruptly halting the use of corticosteroids can cause rebound exacerbation and is therefore not recommended [131]. Due to adverse side effects, it is not recommended that high potency steroids be employed for longer than twice per week for up to four weeks [140]. The AAD recommends switching to a lower potency steroid when the lesion begins to heal [140]. Vitamin D analogs, topical retinoids, and calcineurin inhibitors can be used as maintenance therapy or in combination with corticosteroids to avoid potential recurrence upon corticosteroid discontinuation [125]. Proactive treatment with any previously listed topical treatments is suggested in locations where recurrent lesions are common [140].

Calcineurin inhibitors are also common topical treatments for psoriasis, especially on skin more prone to atrophy, such as on the face and intertriginous areas [135]. Despite not being FDA approved for psoriasis treatment, they are frequently used in place of steroids for prolonged use of more than four weeks. Common side effects of topical calcineurin inhibitors are pruritus and burning, but both can be avoided by applying the treatment to dry skin [124,134]. In this treatment, pimecrolimus or tacrolimus 0.1% is applied twice daily for eight weeks. A combination of tacrolimus and 6% SA can be used with the same instructions [124]. 

Vitamin D analogs are topical treatments often used for mild to moderate plaque psoriasis [124,125]. The two common vitamin D analogs used in the US are Calcipotriene and calcitriol. They are effective when applied topically twice daily for four-to-eight weeks and are often combined with steroids [124,134]. Another common schedule is for TCS to be used twice daily on the weekends while applying topical vitamin D analogs twice daily on weekdays. Side effects include burning, pruritus, edema, peeling, dryness, and erythema, but usually will subside on their own if treatment is continued [140].

Other topical treatments for psoriasis include tazarotene, moisturizers, SA, anthralin, and coal tar [124]. Tazarotene, 0.1% gel, is a topical retinoid used to treat mild to moderate psoriasis over four-to-eight weeks once daily use [124]. It is particularly effective in treating nail psoriasis but can lead to erythema, burning, and pruritus [140]. As such, tazarotene contraindicated during pregnancy [124]. Moisturizers can be used in many forms, including lotion, cream, ointment, and gels, and can be used in conjunction to reduce itching [140]. Moisturizers can be applied many times a day as needed. SA can be used topically in a 6% concentration to reduce scaling and soften plaques [140]. It is also recommended for use to treat scalp psoriasis. SA is often combined with other treatments, but it is effective alone [140]. For example, the combination of SA and TCS can treat moderate to severe psoriasis and palmar-plantar psoriasis [140].

Additionally, anthralin (topical route) can be used to treat mild to moderate psoriasis in eight-to-12 weeks at 0.1% concentration [140]. Concentration can be increased based on lesions’ response to treatment [140]. Adverse effects occur if the skin is exposed for prolonged periods, so contact of two hours or less is recommended [140]. Treatment with a combination of anthralin and excimer laser has been shown to be more effective than anthralin alone [140]. Coal tar is another topical treatment for mild to moderate psoriasis. Treatment involves the application of 1% lotion as needed and often in conjunction with NB-UVB light [125]. 

Systemic treatments are often used for moderate to severe psoriasis due to the inability of topical treatments to maintain skin clearance. Methotrexate is the most commonly used systemic treatment for psoriasis. Dosing ranges from 7.5 mg to 25 mg weekly in a single dose or three doses over 24 hours [142]. It is typically administered orally, and through subcutaneous and intramuscular injection. Injection is preferred at high dosages to prevent gastrointestinal symptoms associated with oral methotrexate [140]. Methotrexate is associated plethora or adverse effects which include fatigue, anorexia, nausea, stomatitis, infection, reactivation of tuberculosis, hepatitis, lymphoma, pneumonitis, myelosuppression, epidermal necrolysis, and hepatotoxicity [142]. Some of these side effects can be minimized by altering the dose, frequency, and route, while others can be mitigated with folic acid in conjunction with treatment. It is necessary to monitor labs regularly and watch for these complications. 

Another systemic treatment for moderate to severe psoriasis is apremilast [124]. Oral dosing is 30 mg twice daily after beginning the patient at 10 mg and titrating up daily by 10 mg every day for the first five days [124]. Common adverse effects include diarrhea, nausea, upper respiratory tract infection, nasopharyngitis, tension headache, and headache. This treatment is less effective but has much less severe side effects than methotrexate [142]. It is, therefore, appropriate for patients with contraindications to methotrexate or who prefer to avoid injections.

Other systemic treatments include cyclosporine and acitretin. Cyclosporine is an immunosuppressant used to treat severe psoriasis and erythroderma. For moderate disease, the dosage is 2.5 to 3.0 mg/kg/day twice a day for four weeks before increasing the dose by 0.5 mg/kg/day until sores are under control [142]. The other option is effective for severe disease and entails a 5 mg/kg/day starting dose but is titrated off once control has been retained [142]. It is common for patients to relapse after this treatment is discontinued, so it should be replaced with a different treatment [124]. The most common adverse effects of cyclosporine use are nephrotoxicity and hypertension, so patients should be closely monitored while using this medication [128,130].

Immunocompromised patients with severe psoriasis can pose a challenge to treat. In these patients, Acitretin, a biologic agent with no immunosuppressive properties is a viable treatment option [124,131]. The typical dose of acitretin is 10 to 50 mg daily. This medication can take between three and six months to produce a full response [142]. Adverse effects include mucocutaneous symptoms such as xerosis, epistaxis, cheilitis, itching, burning skin, hair loss, retinoid dermatitis, hyperlipidemia, pancreatitis, and toxic hepatitis [128,142]. Due to its high toxicity and severe adverse effects, patients on acitretin must undergo regular laboratory monitoring for organ damage once per month for the first three months followed by three months intervals throughout the treatment [124]. 

Phototherapy is an effective treatment for psoriasis, and it is often combined with other treatment options [131,142]. Phototherapy is considered for psoriasis cases refractory to topical treatment and to whom systemic treatments are contraindicated [124]. NB-UVB refers to wavelengths between 311 and 313 nm and is the most common phototherapy treatment for plaque psoriasis [131,142]. Treatment recommendations are two-to-three times per week for an active flare, while maintenance dosing is typically once per week. It is suggested that patients apply a thin layer of emollient before treatment to reduce erythema related to NB-UVB use [143]. 

PUVA is another type of phototherapy that involves photosensitizing agents coupled with UVA light [128,143]. Psoralens can be given orally, applied topically as a cream, or mixed into bath water. Topical PUVA is typically used for palmoplantar psoriasis and is given as 0.1% methoxsalen (8-MOP) with emollient applied 20 minutes before UVA exposure or as 1 mL 1% 8-MOP solution added to 2 L of water for hands and feet to be soaked in 30 minutes prior to UVA exposure [143] This treatment is more effective than NB-UVB but carries the risk of phototoxicity. Bath PUVA is administered as 0.5 to 1 mg/L of 8-MOP in water and is less effective than oral PUVA. However, it carries fewer side effects than oral PUVA and is still used to treat psoriasis. Oral PUVA is typically given in a dosage of 0.4 to 0.6 mg/kg of body weight. Though it is widely effective for treating severe psoriasis in adults, PUVA carries greater risk of adverse effects compared to other types of phototherapy [143]. These effects include phototoxicity, nausea, pruritus, photo-onycholysis, melanonychia, SCC, and nonmelanoma skin cancer in fair-skinned individuals [143].

Biological agents are the definitive treatments used for moderate to severe psoriasis. The most common biological agents used are tumor necrosis factor-alpha (TNF-ɑ) inhibitors, which can be used alone or in conjunction with topical, systemic, and phototherapy treatments [121,132]. The choice of biologic agent is affected by severity, comorbidities, phenotype, lifestyle considerations, and patient preference [128]. Biological agents include etanercept, infliximab, adalimumab, and certolizumab. Etanercept is used to treat plaque psoriasis and is dosed as 50 mg given subcutaneously twice weekly for 12 weeks and then weekly as a maintenance dose. It has been shown as effective in scalp and nail psoriasis [121,132]. Infliximab is used intravenously at 5 mg/kg at weeks 0, 2, 6, and every eight weeks thereafter to treat palmoplantar and nail psoriasis. Adalimumab is dosed as 80 mg subcutaneously for the first dose and then 40 mg the following week and biweekly thereafter to treat plaque and palmoplantar psoriasis [121,132]. Certolizumab is used to treat plaque psoriasis and is administered in two 200 mg subcutaneous injections biweekly [121,132]. Adverse effects of TNF-ɑ inhibitors include hepatotoxicity, lupus erythematosus, congestive heart failure (CHF) exacerbation or onset, cytopenia, and in rare cases, multiple sclerosis. Overall, biological agents are very effective, but its use is complicated by the need for frequent subcutaneous or intravenous administration and potentially dangerous side effects [121,132].

Treatment Consideration for Deployed Settings

Psoriasis in deployed settings can be challenging to treat. Well-controlled psoriasis is not an immediate disqualifier in the military [3,93]. Moreover, maintenance therapy must be continued during deployment to avoid disqualification. However, the majority of available treatment options are automatically barred from use during deployment according to the US Central Command (CENTCOM) standards [93]. Table 5 lists disqualifying psoriasis medications. These medications have serious side effects that compromise mission readiness. Systemic corticosteroids can cause psoriasis flare upon taper [93]. Additionally, methotrexate can cause multiorgan toxicity and requires frequent monitoring that may be unavailable during deployment. Infliximab, anti-TNFɑ, can increase susceptibility for various infections and reactivation of latent tuberculosis [93]. Furthermore, systemic therapies and TNF-ɑ blockers require refrigeration which cannot be guaranteed in a deployed setting [93].

**Table 5:** CENTCOM list of disqualifying medication for deployment Source: [93]

Medication Group	List of Medications
Antimetabolites	Methotrexate
Immunomodulators	Systemic steroids
Immunosuppressants	Abatacept, adalimumab, anakinra, etanercept, infliximab, and leflunomide

Apremilast is a small-molecule biologic therapy approved by the US FDA for use in plaque psoriasis and PsA [93,144]. Additionally, it is approved by the military and does not disqualify soldiers from deployment. While apremilast efficacy based on PASI score (28-30%) is less than methotrexate (36%), adalimumab (70-79%), and etanercept (47-49%), it remains a viable option for service members [93]. The cost of apremilast is high and may cost the Department of Defense $17,808 annually per individual [93]. As such it is mainly reserved for perideployment and deployment treatment. The initial dosage of apremilast is 10 mg once in the morning then increases by 10 mg daily over six days. Maintenance therapy is 30 mg twice daily [93].

Warts

Warts Subtypes

Warts are benign tumors of squamous epithelial cells caused by the human papilloma virus (HPV). Warts can be categorized into anogenital and non-anogenital warts, the latter of which can be subdivided into common warts, plantar warts, flat warts, and filiform warts [145]. 

Anogenital warts (condyloma acuminata): Anogenital warts are caused by HPV types 6 and 11 [146]. Anogenital warts commonly affect the glans of the penis in males, the vulva and cervix in females, and the anus, perineum, and rarely the urethra [146]. Clinically, anogenital warts appear as white papules that coalesce to form exophytic cauliflower-like lesions. These lesions are often painless and nonpruritic [146]. 

**Figure 9:**
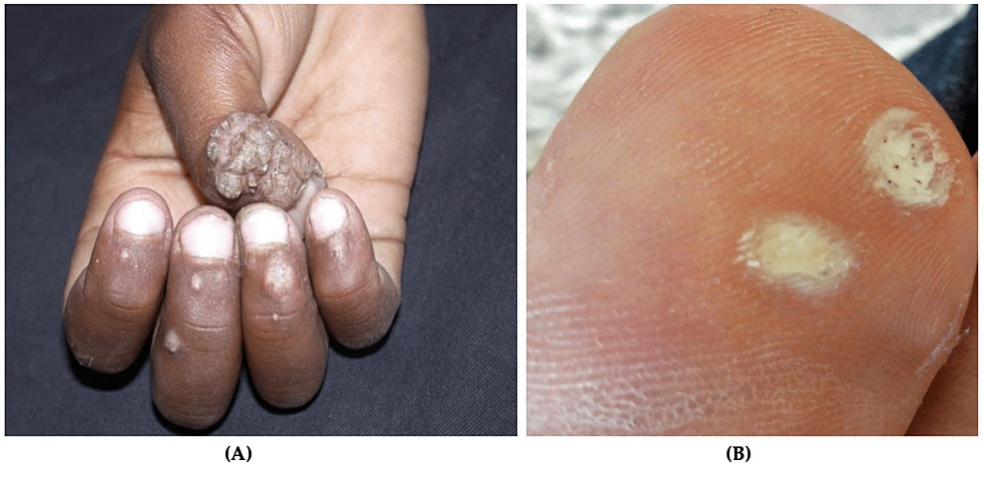
Warts (A) Common warts, multiple white plaques, and papules are visible on the distal end of the fingers. A larger hypertrophic plaque with a verrucous and scaly surface can be seen on the distal end of the left thumb; (B) Two round, well-defined, flat lesions with a rough keratotic surface are visible on the plantar surface of the great toe Source: Figure 9A. Source: Atlas of Paediatric HIV Infection by Regina E. Oladokun, Rannakoe J. Lehloenya, Carol Hlela et.al., Openbooks, licensed under CC BY-ND 4.0; Figure 9B. Verruca vulgaris on the first toe by Mndno, Wikimedia Commons, licensed under CC BY-SA 3.0

Common warts (verruca vulgaris): Common warts are caused by low-risk HPV types 2 and 4. The lesions appear as flesh-colored plaques or papules, usually firm with rough and scaly surfaces (Figure 9A) [145].

Plantar warts (verruca plantaris): Plantar warts are of two types-myrmecia warts caused by HPV 1 and mosaic warts caused by HPV 2 [145]. Plantar warts usually grow inward due to continuous pressure from walking [145]. The mosaic type is more common and presents as superficial, raised, hyperkeratotic small warts growing in close vicinity in one area [147]. Mosaic warts are generally painless and notorious for longevity and treatment resistance [147]. Myrmecia warts appear as a rough, horny surface projecting slightly above the skin surrounded by a collar resembling an anthill. Myrmecia warts commonly occur on the soles, toes, and under nails (Figure 9B) [148].

Flat warts (verruca plana): Flat warts are mainly caused by HPV types 3 and 10 [145]. Compared to common warts, flat warts are smoother, smaller, and have more irregular borders. These lesions appear as multiple small, flat patches or plaques localized to the face, hands, and shins [145]. Flat warts are rare and are mainly seen in immunocompromised patients, young women, and children [149]. 

Filiform warts (verruca filiformis): Filiform warts are caused by HPV 3. They occur most commonly on orifices, face, and beard areas and are typically solitary, though multiple warts may be seen [150]. These appear as soft, raised hyperkeratotic lesions that may be flesh-colored, gray, or brown.

Evaluation 

Evaluating plantar warts is prudent to assess military readiness and preparedness for deployment. Warts can be extremely painful and thus hinder soldiers’ mobility and performance during training and combat [151]. It is prudent for military physicians to obtain a thorough history regarding the duration of symptoms, quality of pain, personal hygiene practices, prior treatments, and occupation. Diagnosis is usually made clinically and without the requirement of special diagnostic tools, though dermoscopy may be helpful in lesion diagnosis [152]. A detailed visual inspection of warts usually yields an accurate diagnosis. Depending on the type and anatomic location, warts can be painful or nonpainful. Many patients will complain of pain with direct compression during ambulation [151]. Treatment-refractory lesions should be evaluated histologically to rule-out amelanotic melanoma [151].

Standard of Treatment

In most cases, warts are self-limited and will resolve spontaneously. Currently, no treatment is available for the serotypes of HPV that cause warts [153]. Instead, treatment is directed at problematic lesions. Treatment modalities for warts are numerous, and the choice of treatment depends on the patient's preference, pain scale, anatomic location, and availability of resources [141,154-164]. Multiple rounds of treatment may be needed (Table 6).

**Table 6:** Conventional treatment options for common and plantar warts Source:  [141,154-164] 5-FU: 5-Fluorouracil, MCA: Monochloroacetic acid; SA: Salicylic acid

Warts: Treatment Options		
Destructive Methods		Non-Destructive Methods
Physical Methods	Chemical Methods	Antimitotic agents: 5-FU and Bleomycin; antiviral agents: Cidofovir; immunotherapy: Interferon, and Imiquimod.
Cryotherapy, curettage/surgery, laser/phototherapy, and duct tape.	Combination of acids including SA, formic acid, and MCA. Cantharidin, silver nitrate, and retinoids may also be utilized for treatment.	

Destructive modalities:Cryotherapy is considered the standard of care for plantar warts. It can be employed separately or concurrently with SA [154]. Liquid nitrogen is sprayed directly on the wart or indirectly using a cotton applicator. Direct application can be painful and may cause blistering. Cryotherapy has 50-70% effectiveness in clearing the infection [141].

Electrosurgery and curettage and surgical excision are other modalities used to remove warts. Excision is associated with a high risk of scarring [155]. As such, it is not recommended as a first-line treatment for plantar warts.

Carbon dioxide or pulse dye laser (PDL) are other physical methods to treat plantar warts. Compared to the carbon dioxide laser, the PDL is more direct, tissue-selective, and causes less thermal tissue damage to the surrounding area [156-158]. Phototherapy requires special equipment. It is mainly preserved to treat facial warts and other areas where cosmetic outcome is most important [159].

Chemical procedures:Topical keratolytic agents are available to treat warts. These often combine SA with lactic acid, such as Occlusal®, Verrugon® complete, and Salatac® [165]. Mediplast SA pads are also available. These keratolytic topical treatments require daily application for several weeks. Currently, SA is considered the most effective treatment for warts [166].

Another chemical treatment option for plantar and common warts is monochloroacetic acid (MCA). It is a powerful irritant agent that dermatologists and podiatrists have employed in the treatment of warts for decades [141,160]. MCA has been shown to be more effective for treating plantar warts in office settings than cryotherapy combined with SA [136]. Patients reported less treatment pain with MCA compared to cryotherapy [136]. While MCA is considered safe, it should not be applied to more than 0.3% of the BSA. Systemic toxicity has been observed when 80% solution of MCA is applied to more than 5% of the body area [161]. 

Formic acid is a safe, simple, cheap, and painless topical treatment option for plantar and common warts [167]. Multiple studies have demonstrated that 85% formic acid (EndWarts® PEN) is a safe and effective treatment for common warts with minimal side effects and favorable compliance [167]. While adverse effects of formic acid are limited when applied correctly, prolonged exposure can result in chemical irritation and severe burns [167]. 

Other chemical treatments include cantharidin, which has been shown to be very effective in the treatment of plantar warts with the combination of SA and podophyllotoxin [143]. Also, silver nitrate is an effective treatment, but may lead to local inflammation and grayish skin discoloration [168]. Tretinoin (vitamin A derivative) 0.05% cream has shown effectiveness for the treatment of facial warts [169].

Antimitotic and antiviral treatment: Antimitotic agents prevent the proliferation of infected keratinocytes by inhibiting DNA synthesis directly or inducing apoptosis through cell cycle disruption [170]. 5-fluorouracil (5-FU) is a DNA analog that inhibits DNA replication and halts cellular proliferation. A weekly injection of 5-FU for four weeks has been shown to be effective in clearing plantar warts [170]. 

Bleomycin is a cytotoxic glycopeptide antibiotic. Intralesional injection of bleomycin has been shown to be effective in treating palmoplantar and periungual warts [162,163]. Potential side effects include scarring, change in pigmentation, nail damage, and Raynaud's phenomenon [162,163].

Additionally, cidofovir, a potent nucleoside analogue antiviral agent has been shown to be effective in the treatment of recalcitrant warts. It can be given in 1 to 3% topical formulation and intralesional injection [171]. The intralesional formulation is shown effective 15 mg/mL once monthly [172]. Both topical and intralesional cidofovir formulations are preserved to resistant cases of warts and should not be used as first-line therapy.

Immunotherapy: Immunotherapy can be used for refractory warts that are resistant to other treatments [173]. The use of these treatments depends on the availability, patient's preference, immune status, price, anatomic location of the lesions, and potential adverse effects [173].

HPV-infected tissues release interferon-alpha, which stimulates the immune system to fight the infection. However, intralesional injections of interferon have not been shown to be effective in the treatment of warts [170]. Imiquimod is another topical medication that induces the production of interferon-alpha, though its effectiveness has not been demonstrated [164].

Treatment Considerations for Deployed Service Members

Verrucous lesions remain common among active-duty service members. Among verrucous lesions, plantar warts can interfere with the proper mobility necessary to execute missions and thus warrant further discussion. Rigorous training drills and associated boot attire invoke increased humidity, disrupting conditions necessary for ideal foot hygiene. Increased moisture in military boots disrupts skin integrity [174]. This damage leaves the skin vulnerable to infections and ultimately compromises wound healing. The injured epithelium provides a portal of entry to the HPV, manifesting as warts [174]. Furthermore, plantar warts can elicit an incapacitating pain out of proportion to their size [175]. Such pain may hinder soldiers from performing critical tasks.

Warts are highly infectious [145]. Therefore, it is prudent to promptly diagnose and treat these verrucous lesions. Treatment of warts can be especially difficult in remote settings. Cryotherapy may not be feasible because liquid nitrogen requires specialized storage containers. Other treatment modalities such as antimitotic agents or immunotherapies are likely unavailable. SA, however, is a great option for treating warts in a deployed setting. It is inexpensive, widely available, and has no storage restrictions [176].

Additionally, duct tape offers an inexpensive, viable, and practical treatment option for deployed settings [177]. An RCT conducted at Madigan Army Medical Center found that duct tape application provided a higher efficacy than cryotherapy in the treatment of common warts [177]. In the treatment of plantar warts, duct tape should be changed every three to five days. The wart can be rubbed with a pumice stone or emery board when the duct tape is removed [177]. Once the wart has been exposed to air for 10-to-12 hours, another piece of duct tape can be applied. Prevention is the best cure for verrucous lesions. To prevent further transmission, the AAD recommends the avoidance of self-induced excoriation and direct physical contact with warts. Avoidance of moisture is essential for prevention [176]. While this can be challenging for service members, it is recommended to change socks at least once per day and wash feet regularly with soap and water [176].

Acne

**Figure 10:**
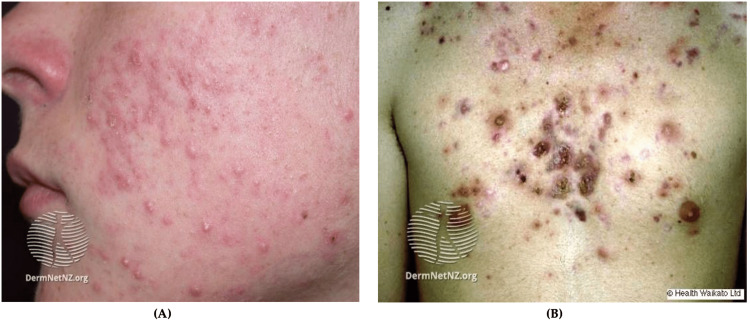
Acne (A) Acne vulgaris; (B) Nodulocystic acne Source: DermNet NZ

Acne vulgaris is an inflammatory condition that affects 85% of people between the ages of 12 and 25 [177,178]. A typical presentation of acne involves comedones, papules, and pustules (Figure 10A). Comedones can be classified into two types: closed (whiteheads) and open (blackheads) [178,179]. In severe cases, patients present with nodules and cysts (Figure 10B). Other symptoms include scars, erythema, and hyperpigmentation [180]. Acne also has profound psychological effects, including low self-esteem, anxiety, and depression due to negative perceptions by others [181,182]. The typical age of onset for acne is puberty but it can frequently present in adulthood [178,179].

Acne is thought to be caused by inflammation, hyperkeratinization, and increased sebum production. Sebum encourages the growth of cutibacterium acnes, the bacterium believed to cause the inflammatory lesions in severe acne [179]. These mechanisms are influenced by genetic factors, age, diet, grooming habits, and other personal behaviors [181,183]. Certain skin types are more prone to acne than others. For example, oily or combination skin can induce acne eruption [184]. Additionally, hormonal dysregulation may alter the production of sebaceous glands and cellular structure around hair follicles [185]. Women with polycystic ovarian syndrome (PCOS) have excess testosterone production, which contributes to acne formation through induction of sebaceous glands and altering cellular structure [186]. In conjunction with other genetic factors and personal behaviors, obesity correlates with acne presentation [180,182]. It is thought that stress, lack of sleep, diet, and cosmetic usage lead to increased rates of acne, but more research is needed to extrapolate definitive causation [180]. Moreover, dairy consumption such as cheese, milk, and yogurt contribute to the development of acne [187].

Acne diagnosis involves clinical evaluation, family history, and related symptoms. Currently, no universal system exists to identify the severity of acne [183]. Acne classification is often completed using the following characteristics: number and type of lesions, disease severity, anatomical sites, scarring, and QOL [183].

Standard of Treatment 

The standard of care for acne treatment includes topical therapies, systemic antibiotics, and hormones [183]. The medication used depends on the severity of the disease, symptoms, and gender. Mixed or primarily inflammatory acne is often treated with topical retinoids alone or with topical retinoids and oral antimicrobials [183]. Topical dapsone 5% gel is used for inflammatory acne, specifically adult females [183]. Post-inflammatory dyspigmentation is treated with azelaic acid topically. Topical antibiotics like clindamycin must be used as maintenance therapy and are required following oral antibiotics; however, it is not recommended to use topical antibiotics alone due to bacterial resistance [183].

Tetracyclines (tetracycline, doxycycline, minocycline) are the recommended systemic antibiotics [183]. Refractory acne in patients with contraindications to tetracyclines may be treated with TMP-SMX and TMP [183]. Oral antibiotics treatment duration should not exceed 3 months due to concerns for antibiotic resistance and should never be used alone. Side effects of systemic antibiotic use include irritable bowel syndrome, pharyngitis, and infections [183].

Contraceptives alone, or in conjunction with other therapies, can treat acne in women. Inflammatory acne is frequently treated with oral contraceptives containing estrogen. It is important to note that this modality can take a significant amount of time to show improvement [183].

The first line of treatment for mild acne is benzoyl peroxide (BP) alone or in conjunction with topical antibiotics, retinoids, or both [183]. Topical retinoids are also prescribed as solo therapy. It is not recommended to use oral antibiotics in patients with mild acne [183]. Moderate cases are commonly treated with combinations of BP and antibiotics, retinoid and BP, or retinoid, BP, and antibiotics [183,188]. Oral or topical antibiotics are also used, though oral antibiotics are typically reserved for cases that are resistant to topical treatments. Severe acne is generally treated using oral antibiotics with BP, retinoids, or both [183]. Another option for treatment-resistant acne is oral isotretinoin. While isotretinoin is being used, it is necessary to monitor liver function, serum cholesterol, triglycerides, and symptoms of depression [183]. It is also imperative that women taking isotretinoin be counseled on contraceptive methods due to teratogenicity. It is typically recommended to use combination treatment to treat multiple potential pathologies [183,188].

Treatment Considerations for Deployed Service Members

In the armed forces, severe acne often prevents young men and women from enlisting [3]. It is also common for soldiers to avoid seeking treatment for acne due to concerns about limited consultations available and the feeling that acne is not a severe enough issue to warrant treatment [3]. Occlusive garments combined with mild acne often lead to nodulocystic acne on the soldiers' chests and backs [2].

Isotretinoin is a widely available treatment for severe acne, but its use in the military renders service members non-deployable [188]. Frequent lab testing is required, and severe photosensitivity is a known adverse effect of this medication which could be difficult to tolerate for service members in sunny climates [188].

Though effective in the treatment of acne, doxycycline requires the grounding of service members from deployment to monitoring for adverse effects [3]. Like isotretinoin, doxycycline can cause photosensitivity [188]. Doxycycline also raises the concern of antibiotic resistance. PDL is an alternative therapy for acne without the significant adverse effects and operational constraints of isotretinoin and doxycycline [188]. In one case report, PDL resolved inflammatory acne in a 24-year-old active-duty male servicemember [188]. The patient had no reported adverse effects from the treatment. As such, PDL should be more widely available in deployed settings to offer treatment for service members suffering from severe acne [188]. 

Keloids and hypertrophic scarring

Keloids and hypertrophic scars are most seen in Black, Hispanic, and Asian patients and equally affect men and women [189]. The pathogenesis of keloids and hypertrophic scars is not completely understood, though they are thought to involve fibroblast dysfunction and genetic predisposition [190].

**Figure 11:**
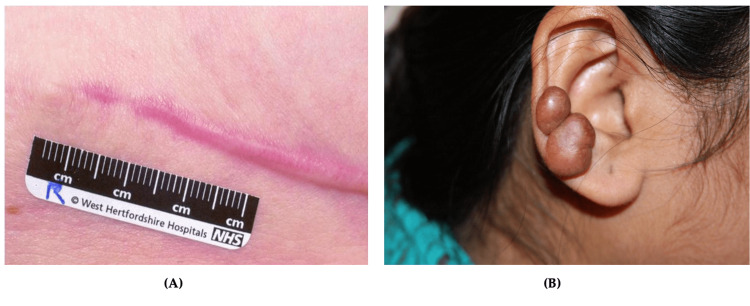
Keloid scars and hypertrophic scars (A) A linear, erythematous raised scar is visible. These features are characteristic of a hypertrophic scar, which results from abnormal wound repair (i.e., overgrowth of fibroblastic tissues during skin healing); (B) Two well-defined round keloids with a red, smooth surface in the right pinna Source: Figure 11A: Cutaneous Scarring: A Clinical Review by Richard Baker, Fulvio Urso-Baiarda, Claire Linge, Adriaan Grobbelaar, Dermatology Research and Practice Journal, licensed under CC BY 3.0; Figure 11B: Ear Keloid-Auricular Keloid by Htirgan, Wikimedia Commons, licensed under CC BY-SA 3.0.

Anatomical sites of keloids most commonly involve the chest, shoulders, upper back, neck, and earlobes [191]. Keloids can develop within one day and up to 24 years following dermal trauma [191]. Keloids can present as either minor keloids or major keloids (Figure 11). Minor keloids are focally raised, pruritic, and extend beyond the site of the trauma. Alternatively, major keloids are large (>0.5 cm), painful, pruritic, and extend beyond the site of the trauma (Figure 11B) [192]. Both minor and major keloids do not regress spontaneously and may progress in size over the years [192].

Hypertrophic scars are categorized as either linear or widespread [193]. Linear hypertrophic scars often develop after surgical or traumatic scars. Contrastingly, widespread hypertrophic scar often develops after a burn incident [193]. Linear hypertrophic scars are characterized by red, raised, and pruritic lesions confined to the scar's border (Figure 11A). Such lesions develop an elevated, slightly rope-like appearance. Widespread hypertrophic scars are red, raised, pruritic, and remain within the border of the burn injury [194]. Diagnosis of keloids and hypertrophic scars is made clinically based upon the patient's history and physical exam [190]. Skin biopsy is unnecessary but helps exclude other conditions that mimic keloids or hypertrophic scars [190].

Standard of Treatment 

Keloids are challenging to treat and often require more than one treatment to achieve satisfying results. The AAD recommends the following treatments for keloids: intralesional corticosteroids, intralesional 5-FU surgical removal, laser treatment, silicon sheet and gels, cryotherapy, pressure therapy, radiation treatments, or ligature [195]. 

Intralesional triamcinolone at concentrations of 10 to 40 mg/mL is the most common treatment for keloids and hypertrophic scars [196]. Scars that do not respond to intralesional triamcinolone can be treated with intralesional 5-FU monotherapy or in combination with intralesional corticosteroids [196]. The concentration for intralesional fluorouracil that is often used is 50 mg/mL every four weeks for three treatments. Intralesional 5-FU can cause injection site pain and hyperpigmentation [197].

Silicone gel sheets are another preventive and therapeutic option for keloids and hypertrophic scars [198]. The mechanism by which silicone gel sheets work is unknown but is thought to provide hydration, reduce mast cell activity, and generate static electricity [198]. Pressure therapy can be used for patients with earlobe keloids. This therapy works by occluding small blood vessels in hypertrophic tissues, thereby reducing oxygen supply which leads to atrophy of these tissues [199,200]. Pressure earrings for earlobe keloids are available commercially. Other devices using magnets with or without silicone are also available [198].

Cryotherapy is a significant treatment option to consider for patients who do not respond well to intralesional steroids or intralesional fluorouracil [201]. Additionally, intralesional cryotherapy was developed to specifically target keloid and hypertrophic scars without affecting the surrounding area. For traditional cryotherapy, a 10-30 second freeze-thaw cycle is used and can be repeated up to three times per treatment session. For intralesional cryotherapy, the freezing time is generally longer and is dependent on the keloid size [202].

If conservative treatments fail to produce successful results, surgical excision of keloid and hypertrophic scars should be considered [203]. However, recurrence of keloids after excision is very common. Therefore, it is recommended that intralesional corticosteroids, intralesional fluorouracil, cryotherapy, or radiation therapy be implemented during and after the surgical excision [203].

Treatment Considerations for Deployed Service Members 

Due to the nature of their job, military personnel often sustain major traumatic wounds and are therefore at high risk of developing keloids. Some keloids may be large and cause functional impairment and disfigurement [19]. Therefore, dermatologists on-site or other clinicians must be aware of patients with these conditions and promptly provide treatment. Postponing treatment can result in irreversible keloids [19]. Keloids are potential disqualifiers for deployment in the military if it interferes with the proper use of military apparel and equipment [204]. Table 7 summarizes the similarities and differences between keloid scars and hypertrophic scars.

**Table 7:** Clinical comparison of keloid scars versus hypertrophic scars Source: [189,190]

	Keloid Scars	Hypertrophic Scars
Definition	Skin lesions are caused by fibroblast proliferation and collagen production as an excessive tissue response to typically small skin injuries.	A cutaneous condition characterized by high fibroblast proliferation and collagen that leads to a raised scar that does not grow beyond the boundaries of the original lesion.
Etiology	Imbalance in wound healing.	Imbalance in wound healing.
Clinical Features^1^	Brownish-red scar tissue with varying consistency localized mainly on the earlobes, face, and upper extremities. Pruritis may be present in some patients.	A raised scar that does not grow beyond the boundaries of the original lesion.
Diagnosis	Diagnosis is based on the clinical appearance of the lesion and history of trauma or surgery.	Diagnosis is based on the clinical appearance of the lesion and history of trauma or surgery.
Prevention	Avoidance of repetitive skin trauma and occlusive clothing.	
Treatment	Intralesional steroids, intralesional fluorouracil, cryotherapy, or pressure garments.	
Prognosis	Does not regress spontaneously. High likelihood of recurrence after resection.	Regresses spontaneously.

It is understandably challenging to maintain regular check-ups and treatment schedules during deployment. Treatment options are limited during deployment, especially in remote settings [19]. In such settings, treatment goals should include pain relief, scar volume reduction, and improved functionality [19]. Patients and clinicians should work together to maintain a regular check-up schedule and monitor the progression of the scar.

Traction alopecia

Traction alopecia is a form of hair loss resulting from prolonged and repetitive traction on the hair [203]. Traction alopecia most commonly occurs in African American females and is thought to be associated with the frequent use of hairstyles such as braids or hair waves [205]. The prevalence of alopecia in the military is higher than that in the civilian population. From 2010 to 2019, it was estimated that 2.40% of 498,219 active-duty servicewomen (ADW) were diagnosed with alopecia over 10 years [206]. The pathogenesis of traction alopecia has not been elucidated, but prolonged traction of the hair is thought to cause perifollicular inflammation, resulting in reduced hair density or thinning of hair and eventually hair loss [207,208].

Active-duty women have very stringent grooming standards to meet their occupational demands and maintain a professional appearance [19]. Servicewomen must wear their hair in tight ponytails, buns, or braids [19]. Combined with chemicals and exposure to heat, these hairstyles exacerbate traction alopecia [19].

**Figure 12:**
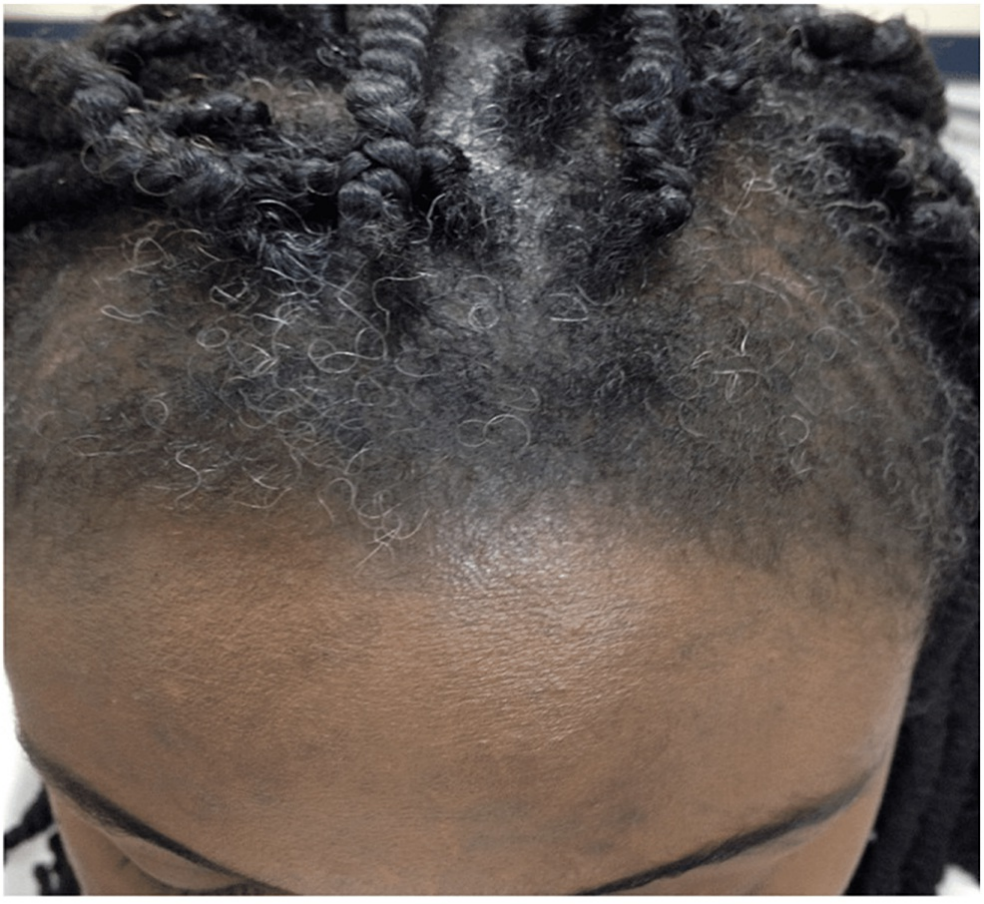
Traction alopecia Marginal alopecia in a patient with braids Source: Traction Alopecia: The Root of the Problem by C. Billero and M. Miteva, licensed under CC BY-NC 3.0.

Clinical findings of traction alopecia include inflammatory papules, pustules, thinning of hair, and alopecic patches [205]. Traction alopecia most frequently involves the frontal hairline and temporal scalp above the ears, but other areas of the scalp can be affected. Traction alopecia is characterized by transient hair loss in most cases. However, persistent tension on the scalp can progress to marginal alopecia which involves frontal and temporal hair loss (Figure 12) [209]. Non-marginal alopecia involves areas other than the frontal and temporal scalp. It is commonly seen in women who wear their hair in a tight bun which can cause hair loss in the occipital scalp [209]. Patients often present with collections of scale surrounding the hair shaft, which is a sign of ongoing traction alopecia [209]. 

Diagnosis of traction alopecia can be made based on clinical evaluation and patient history. A history of hair loss associated with traction hairstyles is essential for diagnosis, which can be confirmed by biopsy [208]. Management of traction alopecia focuses on early detection of the condition and preventative measures. Early detection is associated with better clinical outcomes and potential resolution of the condition [208]. Additionally, patients are recommended to discontinue using traction hairstyles that exerts tension on the hair [208,209].

Standard of Treatment

In addition to the preventative measures for traction alopecia, adjunctive interventions can be utilized for treatment, such as topical minoxidil, local corticosteroids, oral antibiotics, hair transplantation, and cosmetic camouflage [210]. Minoxidil is prescribed for patients with early-stage traction alopecia with no signs of active inflammation. Patients are instructed to apply minoxidil 5% solution or foam daily to the scalp [210].

Local corticosteroids can be used in patients with traction alopecia and concurrent inflammation [208]. Medium to high potency corticosteroids are usually preferred to reduce inflammation through intralesional injection [211-213]. TCS therapy is generally applied twice daily to the entire affected scalp. Alternatively, intralesional triamcinolone acetonide may be used. Once the inflammation resolves, the treatment should be discontinued [208]. 

Oral antibiotics such as tetracyclines have anti-inflammatory properties which can be used to treat inflammatory traction alopecia [212]. Oral antibiotics are preferred over local corticosteroids due to fewer side effects. The typical adult dosage for adults includes tetracycline 500 mg twice daily, doxycycline 50 to 100 mg twice daily, or minocycline 50 to 100 mg twice daily [212].

Considerations for Military Grooming Standards

Grooming guidelines have been an integral part of the US military professional standards [214]. Military hair guidelines shape the daily lives of service members and likewise can impact the quality of hair shafts and overall scalp health. Historically, military service members in the US military have been required to adhere to strict hair-grooming criteria with limited hairstyle options [214]. Previous military hair grooming standards for females were limited to two hairstyle options: a tucked-in braid or a bun and long ponytails. Untucked braids were not permitted [214]. 

Previous grooming criteria did not account for ethnic and racial differences of hair differences. Military grooming standards describe various approved hairstyles. However, these conditions can cause an array of hair conditions. Such hairstyle criteria subject hair follicles to continuous strain and can lead to the eventual development of traction alopecia. Traction alopecia disproportionately affects servicewomen as they must comply with strict hairstyle standards [19]. Furthermore, these strict military guidelines can lead to extracranial headaches, trichorrhexis nodosa, hair shaft dysfunction, and hair breakage.

To support inclusivity and racial diversity, the Department of Defense enacted unprecedented hair-grooming policies in 2020 and these updated guidelines were officially implemented in 2021 [215-217]. As of 2021, the US Navy, Army, and Airforce allow female service members to wear their hair in a full ponytail or braid(s) [215-217]. These more inclusive standards also support allow female service members with various types of hair shafts and density. These new guidelines allow service members to have more flexibility with styling their hair while maintaining a professional look [214]. 

The updated military grooming guidelines, with regard to hair length, vary among the Army, Air Force, and Navy Branches [215-217]. The US Air Force and Army have authorized longer braids and ponytails with varying length requirements [215-217]. The US Air Force requires airwomen to wear their ponytails or braids from below the crown of the head extending to the top of each sleeve inseam at the underarm [216]. During the same year, the US Air Force authorized the wear of ponytail bulk up to 12 inches, which extended from the previous mandate of 4 inches in 2020 [216]. Contrastingly, the US Army grooming criteria require that ponytails and braids start below the crown and do not extend past the bottom of the shoulder blades, rather than the top of each sleeve inseam at the underarm (US Air Force) [215-217]. According to Navy grooming criteria, hair length may touch, but not fall below, a horizontal line that is level with the lower edge of the back of the collar [217].

The US Navy grooming guidelines specify that service members are free to choose the type of hairstyle in the Navy as long it promotes a professional military appearance [217]. Lopsided and extremely asymmetrical hairstyles are not authorized. Buns and ponytails are authorized in the Navy. All hairstyles will be evaluated by its appearance when headgear is worn to ensure comfort and practicality. Hair bulk (minus the bun) as measured from the scalp will not exceed 2 inches. The bulk of the bun will not exceed 3 inches when measured from the scalp and the diameter of the bun will not exceed or extend beyond the width of the back of the head [217].

Looking ahead, the US Department of Defense should continue to modify its grooming standards in ways that support inclusivity and racial diversity. The Department of Defense continues to update the grooming guidelines to avoid hair traction and prevent chronic hair loss and hair disease. Likewise, other important measures should be taken to avoid preventable hair disease, including physician awareness and patient education. Physician awareness and patient education are crucial in the early discovery of hair disease. Clinicians should educate patients on the treatments available in deployed settings and provide enough minoxidil for the duration of their service [19]. Likewise, early intervention may improve the prognosis and reduce the severity of the disease. Patient education is a cornerstone in preventable hair loss as service members can proactively mitigate hairstyles that are compliant with their military branch grooming guidelines. To reduce hair damage, patients are recommended to avoid tension-based hairstyles as much as possible and avoid hair products that cause hair damage. 

The updated grooming standards promote a variety of benefits to service members and overall mission readiness [19]. Benefits include less hair condition, improved cutaneous body image (CBI), improved morale, and improved mission effectiveness [218]. With regard to hair conditions, the updated hair-grooming regulations are expected to decrease the prevalence of female service members impacted by hair disorders. Likewise, positive impact on morale and likewise improves self-esteem and mission readiness among female service members [218]. Hair loss conditions, such as traction alopecia, can have devastating psychological impacts on female service members [218]. Avoiding the aforementioned preventable hair conditions will improve combat effectiveness and decrease medical evacuations, thereby supporting operational budgets. These administrative changes promote the prevention of avoidable hair loss and underscore the value of its contributions to combat readiness. Given the long-term detriment to the morale of service members and overall mission readiness, military hair standards should be further evaluated to reduce the incidence of traction alopecia and other preventable conditions associated with grooming standards. Such changes will positively impact service members, reduce avoidable hair loss and improve mission readiness among female service members.

Hidradenitis suppurativa (HS)

**Figure 13:**
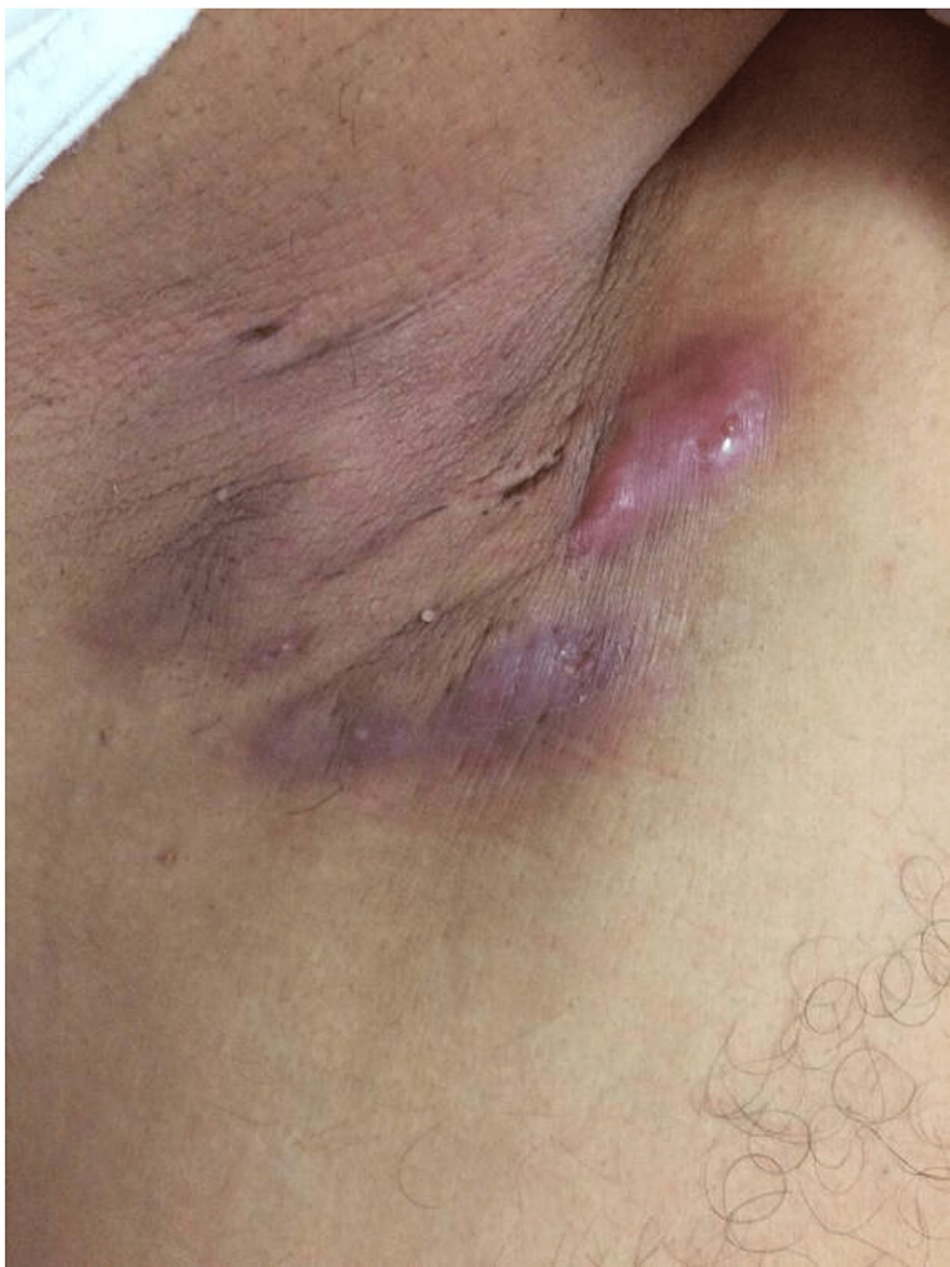
HS Multiple erythematous lumps in the axilla Source: Fluorine-18 Fluorodeoxyglucose PET/CT Images of Hidradenitis Suppurativa Mimicking Metastasis in a Patient with Small Cell Lung Carcinoma by O. Ekmekcioglu, K. Sonmezoglu, Cureus, licensed under CC BY 4.0. HS: Hidradenitis suppurativa

HS is a debilitating chronic inflammatory occlusive dermatological condition that affects hair follicles and apocrine glands. HS presents mainly, but not exclusively, in folded skin areas such as the axilla, groin, inner thigh, and perineal area (Figure 13). The disease severity can range from mildly painful papules and fistulas to the formation of deep abscesses, draining sinuses, and co-infections with scarring [219, 220]. The pain, odor, and disfigurement caused by HS can reduce the QOL of active military personnel due to physical, emotional, and psychological consequences. 

The Hurley staging system classifies the severity of HS into three distinct groups based on the presence of lesions, scarring, and sinus tracts. In Stage I, an abscess develops, but no sinus tract formation or cicatrization is present. In Stage II, there are recurrent abscesses with sinus tract formation and cicatrization. Stage III consists of diffuse abscess formation with multiple interconnected sinus tracts and widespread cicatrization [219,221,222].

HS can greatly impact the physical, psychological, and emotional health of military personnel, thereby reducing their performance and potentially endangering them in active combat settings [223]. As such, clinicians should provide comprehensive assessments and adequate treatments for these patients [223]. HS most commonly presents with abscesses that often rupture and release pus. Thus, patients should be educated on appropriate wound care to minimize skin trauma. It is recommended that patients use simple white petrolatum to prevent dressings from sticking to wounds. Topical antiseptic washes such as chlorhexidine and benzoyl peroxide are recommended to clean wounds [220]. 

Patients with HS commonly experience severe pain due to inflammatory nodules and abscess formation [224]. Even mild cases of HS can present with pain that causes emotional and psychological distress [224]. The pain can be severe enough to induce sleep disturbance and limit physical function. Some patients report depression resulting from HS [224]. Dermatologists have discovered that when HS is effectively treated, symptoms of depression subside [224]. Pain associated with HS can be effectively treated with NSAIDs, but chronic pain may require a multidisciplinary approach [224].

Lifestyle modification is another essential aspect of the prevention of the recurrence of HS flares. Likewise, smoking and obesity have been associated with HS [220]. It has been shown that the prevalence of obesity and smoking is greater than 75% among patients with HS [220]. Smoking has been associated with more severe symptoms, prolonged duration, and failure of treatment response, though evidence regarding smoking cessation and improvement of HS symptoms are inconclusive [220]. Though data regarding weight loss and the corresponding effect on HS is inconclusive, some reports have demonstrated improvement or resolution of disease with substantial weight loss [220]. Additionally, there appears to be an increased risk of developing PCOS among women with HS. Thus, clinicians should screen for PCOS in women with HS who present physical signs of androgen excess and menstrual irregularities [219].

Standard of Treatment

**Figure 14:**
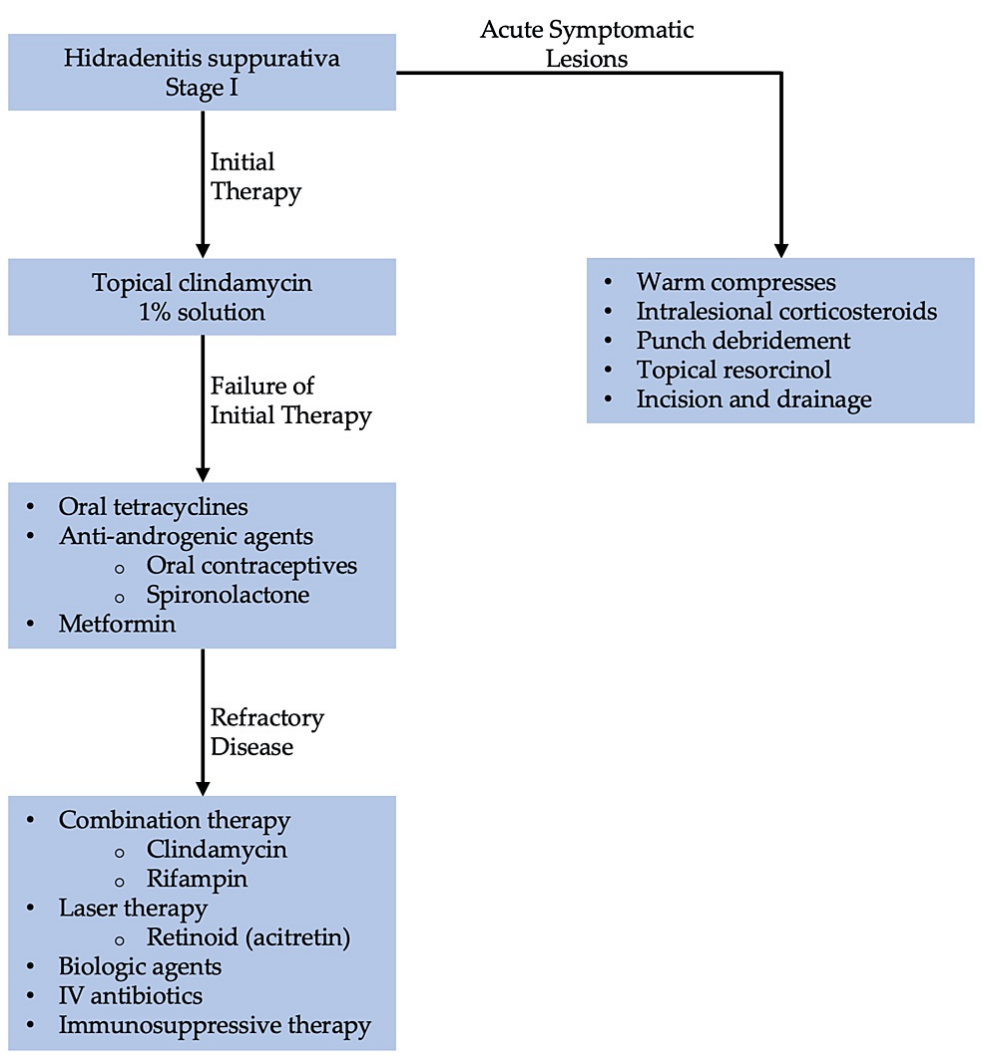
Treatment algorithm for HS Stage I The information in this figure was adapted from Ingram 2022 [224] HS: Hidradenitis suppurativa

The treatment for HS is multifactorial, and it is mainly dependent on the Hurley staging system [225]. For Stage I, which includes inflammatory lesions without sinus tract formation, the goal of the treatment is to prevent the progression of the disease and improve acute, symptomatic lesions (Figure 14) [225]. The initial therapy includes the use of topical clindamycin but can be escalated to oral clindamycin, oral tetracycline, antiandrogen agents, and metformin to resolve flare-ups [224]. It is recommended to apply topical clindamycin twice per day in skin areas prone to recurrent flares. Topical clindamycin is well-tolerated, but some patients may experience slight burning after application. If topical clindamycin is ineffective, oral tetracyclines are the next treatment of choice. The duration of the treatment with tetracycline varies from weeks to months or until satisfactory control of symptoms is achieved. Once oral tetracyclines are discontinued, patients are often prescribed daily application of topical clindamycin for maintenance therapy [224]. 

Anti-androgen therapies may be used if topical clindamycin or oral tetracycline fails to produce sufficient improvement [220]. It is postulated that elevated androgen levels contribute to the development of HS [224]. However, evidence regarding the use of hormonal therapies is limited. Oral contraceptives, spironolactone, and finasteride have been used but efficacy data are limited. Metformin also appears to be beneficial in the treatment of HS [220,224]. 

**Figure 15:**
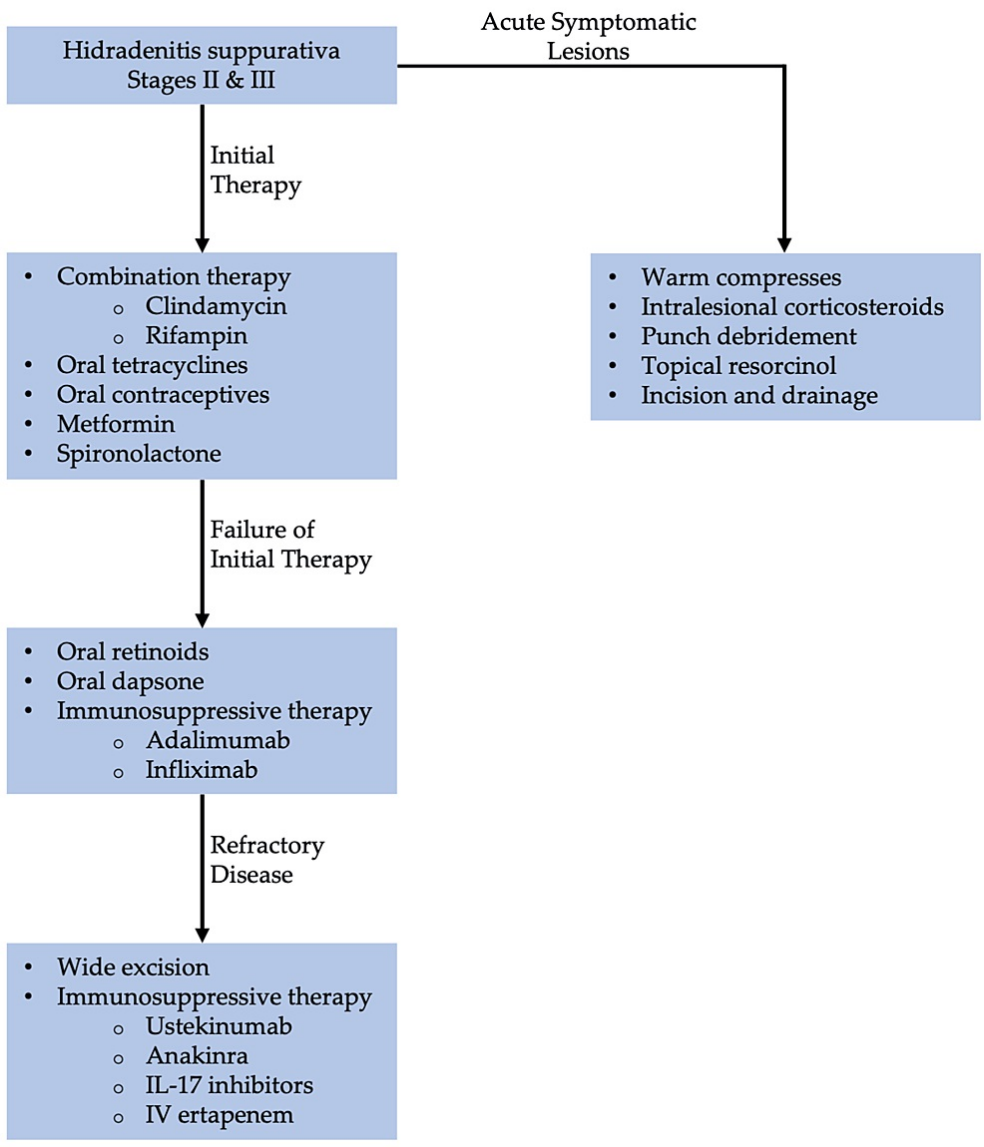
Treatment algorithm for HS Stages II and III The information in this figure was adapted from Ingram 2022 [224] HS: Hidradenitis suppurativa

Patients with HS Stage II or III require more aggressive treatment (Figure 14) [224]. The initial therapy for these patients includes tetracycline, a combination of clindamycin and rifampin, antiandrogens, or metformin. Oral antibiotics such as tetracycline are the initial treatment of choice [224]. It is prescribed similarly to patients who present with mild disease. If oral tetracycline fails to show improvement, a combination of clindamycin and rifampin is recommended as an alternative treatment [224]. Metformin, oral contraceptives, and spironolactone are typically used as adjunctive therapies. If the initial therapy for HS Stage II or III fails, other medications such as oral retinoid, oral dapsone, adalimumab, or infliximab may be considered (Figure 15) [224]. Other medical therapies for these patients include immunosuppressive medications such as ustekinumab, anakinra, and IL-17 inhibitors such as secukinumab [224]. Severe or refractory disease may require intravenous ertapenem or surgical excision of the lesions [224].

Treatment Considerations for Deployed Service Members 

HS is a debilitating dermatological condition that can have detrimental effects on military personnel [223]. Severe cases of HS may restrict military personnel from participating in training obligations, in operational missions, and completing assigned duties [223]. This hinders both the service member and overall military readiness. As such, early detection of HS is important as it reduces disease progression and recurrence. Early detection is essential, and treatment with clindamycin should be initiated as soon as possible [223]. Additionally, patient education and support are essential components of management [223].

Military personnel should be advised to minimize exposure to stressful situations to avoid flare-ups [223]. This is a challenging task in the military, especially in austere environments or combat settings. Unsanitary environments and occlusive uniforms contribute to disease progression and decrease the chance of successful treatment. Wound care with dressing changes should be performed daily to minimize progression of the disease [223]. Clinicians, especially dermatologists, should be present on-site or via video call to provide regular assessments and guidance [223].

Emerging cutaneous infections: monkeypox virus

Monkeypox is a zoonotic, double-stranded DNA virus that belongs to the orthopoxvirus family [226]. The first sporadic monkeypox virus outbreaks were reported in Africa in 1959 in the Democratic Republic of the Congo and Nigeria [227]. The spread was thought to originate from contact with wildlife reservoirs [227]. Monkeypox is a self-limited disease with symptoms lasting two-to-four weeks. Cases of the monkeypox virus have been rare and only limited to central and west Africa tropical rainforest areas. However, as of August 2022, 30,189 monkeypox cases have been reported worldwide, and 8,934 cases have been reported in the US [226]. States with the highest reported cases include New York (1,960), California (1,310), Florida (936), Texas (702), and Illinois (672). Additionally, the US military has been experiencing a concerning increase in cases of monkeypox infection among its service members. Within four weeks, the number of cases rose from four to 40 total cases in all US military branches [228]. 

Transmission of the Monkeypox virus occurs through various routes, including respiratory droplets, direct skin contact with visible lesions, and contaminated fomites [229]. Additionally, vertical transmission from infected pregnant women to fetuses has been reported, which can cause fetal demise [230,231]. Moreover, men who have sex with men, immunocompromised individuals, and people diagnosed with human immunodeficiency virus are at increased risk of infection [232,233].

**Figure 16:**
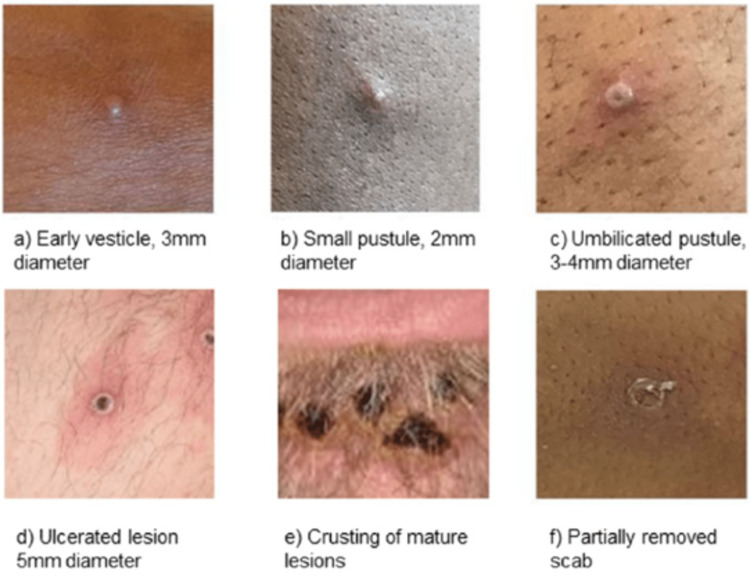
Cutaneous manifestation of individual monkeypox lesions Source: gov.uk; licensed under Open Government License v3.0

Monkeypox infection classically presents with fever as the initial symptom, followed by the development of a rash. Other common symptoms preceding the rash include lethargy, myalgia, headache, and lymphadenopathy. A case series of 528 infections from 16 countries between April 27 and June 24, 2022 revealed that 95% of the persons presented with a rash. The most common anatomical sites of the rash include the anogenital area (73%); the trunk, arms, or legs (55%); the face (25%); and the palms and soles (10%) [233]. The monkeypox rash has a broad spectrum of presentations such as macular, pustular, vesicle, and crusted (Figure 16) [233]. Additionally, mucocutaneous eruptions of Monkeypox may present in multiple phases simultaneously [233]. Monkeypox infection is confirmed via polymerase chain reaction test. Appropriate samples should be collected from visible skin lesions for accurate results [234]. 

At the time of this writing, there is no definitive treatment available for the monkeypox virus. The illness is usually self-limited and resolves spontaneously without complications [230]. Infection prevention education should be widely available to the public to prevent the spread of the infection. Current preventative measures include avoidance of skin-to-skin direct contact when indicated [226]. Respiratory isolation is essential for suspected and confirmed cases. Due to the self-limited nature of the infection and the available measures to prevent the spread, risk and benefit analysis should be conducted prior to administering the smallpox vaccine, currently theorized as a preventative measure. Adverse reactions to the smallpox vaccine can range from mild to fatal [235]. As such, individualized recommendations should be given prior to administering the vaccine. Prevention for this condition is an ongoing discussion and will continue to evolve as more data are collected. 

Teledermatology

Dermatology is a visually dependent specialty and is particularly suited for telemedicine [236]. The major goals of teledermatology in the military focus on practicality, accessibility to specialists, the accuracy of diagnoses, limiting time away from the mission, and minimizing costs associated with medical evacuation and transportation of military personnel to military training facilities [237]. Teledermatology provides a practical platform that allows existing dermatologists to extend themselves and help more patients than in a typical setting [237]. The US military has been increasing the usage of teledermatology to mitigate the shortage of dermatologists. As such, teledermatology is now a well-established platform in military hospitals, making up 40% of telemedicine consultations [5].

Telemedicine provides healthcare services remotely via telecommunications technology [238]. Often, the patient and provider need not be available simultaneously as still images can often be used in lieu of video. This is especially true for dermatology, which relies heavily on visual information for diagnosis [239]. In addition, these methods allow dermatologists to consult, triage, diagnose, and create care plans for active-duty service members stationed across the globe without the costs or dangers of traveling [240].

Dermatological conditions are ubiquitous among military warfighters and contribute to decreased combat effectiveness [237]. Telemedicine is an excellent fit for providing dermatology care to stationed service members. Since the establishment of telemedicine in 2004, teledermatology facilitated over 400 consultations annually, the highest of any specialty in the military [241]. A study by Gregory et al. found that 92 dermatology-specific ICD-9 codes accounted for 10% of all medical diagnoses in deployed service members [241]. Dermatologists play an essential role in timely medical care in deployed settings [242]. As such, teledermatology helps mitigate the demand for specialized care by allowing remote dermatologists to provide instant consultations and thereby improve lesion diagnosis and prognosis [237]. Additionally, teledermatology lessens the burden on primary care physicians (PCPs) to accurately diagnose complex dermatological conditions that may fall outside their scope of practice [237]. A study done in Ireland of 500 dermatological cases revealed that dermatologists were 87% accurate when diagnosing skin cancer in comparison to 22% accuracy among PCPs [243]. Therefore, it is prudent for board-certified dermatologists to provide direct care for service members with dermatological concerns.

Additionally, teledermatology has made consultations and assessments more efficient [237]. Service members utilizing teledermatology can see a specialist within 12-24 hours compared to four-to-eight weeks for in-person appointments [5,225]. Moreover, the Department of Defense's utilization of teledermatology has made dermatology cost-effective. It has prevented unnecessary medical evacuations, facilitated urgent consults, and reduced physicians' cost of travel [237,244]. Teledermatology saved the US military approximately 30.4 million US dollars of unnecessary dermatology-related medical evacuations and travel costs during the Iraq War from 2005 to 2009 [242]. Likewise, teledermatology reduces the time away from the mission thereby increasing military functional readiness [244].

Teledermatology can serve as an avenue for quality control [185]. One study done on the efficacy of store-and-forward (S&F) systems found that teledermatology consultations allowed for more experienced and more specifically trained clinicians to interpret clinical and laboratory findings [240]. The US Army's teledermatology consult services allow multiple dermatologists to view images and clinical history to make comments and provide any additional insights [241]. These collaborative recommendations are then adopted by the PCP on-site who can provide care to service members [242]. 

The two main delivery methods of telemedicine utilized by the US Military Health System (MHS) include asynchronous S&F and synchronous live-interactive (LI). S&F utilizes email or other Internet-based programs to send still photographs of lesions for focused visualization [238]. In this method, images and medical records are accessed asynchronously, allowing the physician to access and respond to these queries at any time [243]. This method requires less sophisticated technology such as a personal computer, existing communication lines, image viewing software, and a digital camera [245,246]. Other benefits of this method include prompt communication and diagnosis, lower cost, similar outcomes to standard care, the ability to educate PCPs remotely, less technological sophistication, and lower cost of equipment [237]. Drawbacks to this method include a lack of immediate interaction between the patient and provider and an inability to ask questions in real-time [245]. 

The LI method utilizes live synchronous video connection for the specialist to communicate with the patient or PCP in different locations simultaneously. As a result, physicians can gather pertinent information from the patient much like an in-person consultations and ask pertinent questions about medical history [237]. This synchronous method also permits the dermatologist to request different camera angles. Likewise, the patient can reveal other areas of skin to determine disease context [238].

**Figure 17:**
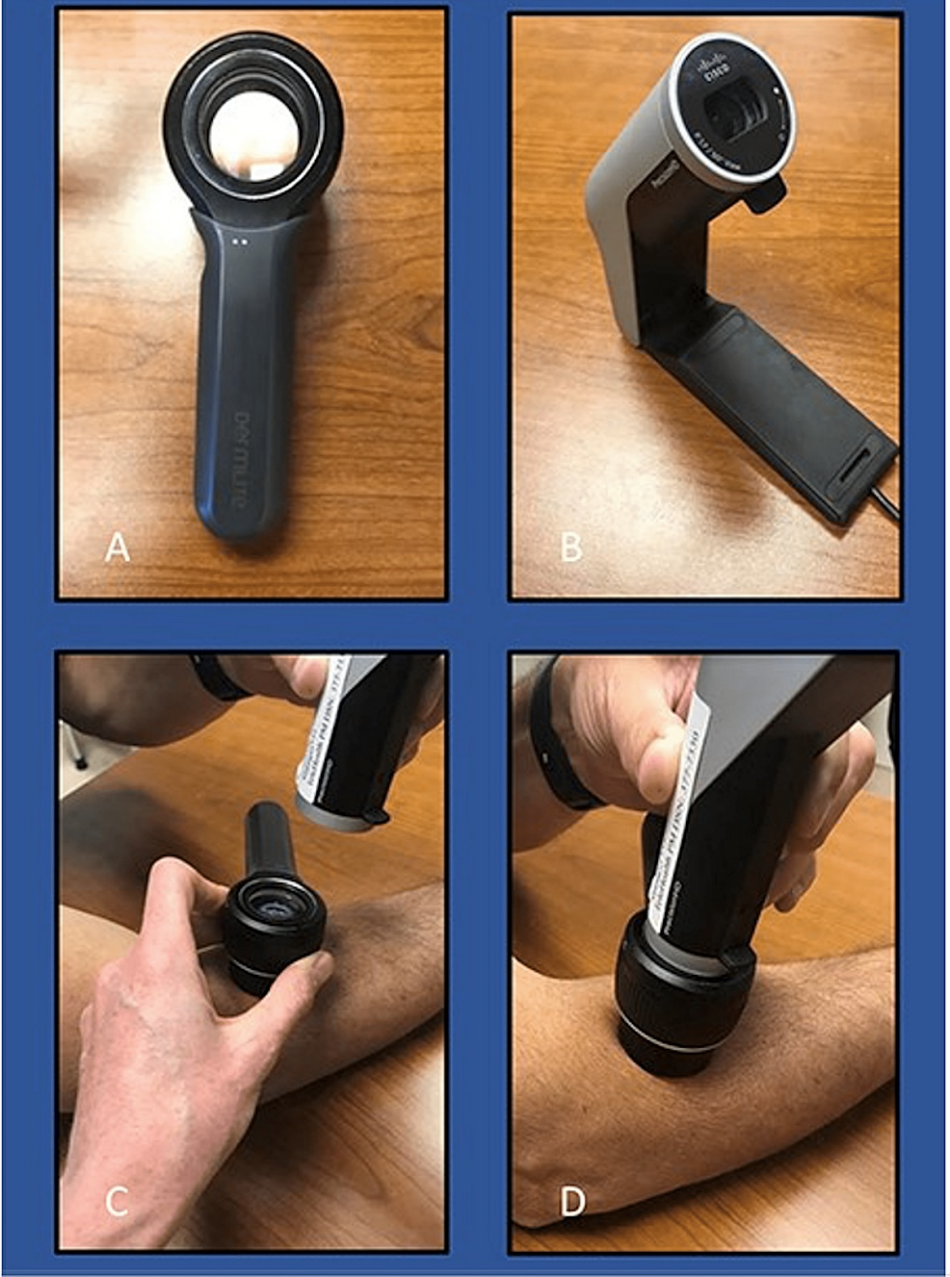
Basic configuration for teledermoscopy (A) Dermatoscope; (B) Cisco Jabber Webcam; (C) Sanitize dermatoscope and skin lesion with alcohol; (D) Gentle contact between webcam and dermatoscope

Teledermoscopy is a valuable diagnostic tool that can be implemented into either asynchronous S&F and synchronous LI formats [244]. To date, only one military study has evaluated the diagnostic efficacy of teledermoscopy in a military training facility (MTF) [244]. This novel study compared the utilization of teledermoscopy in a synchronous LI teledermatology consultation and dermoscopy in a traditional in-person consultation [244]. The study was conducted on two patients at a remote US naval primary care clinic. Each patient presented with two lesions (benign and malignant) that were examined by the two providers [244]. The teledermoscopy session utilized a repurposed webcam (prevalent at most MTFs) and a 10-times magnifying glass with transillumination properties. This research study revealed accurate diagnoses between the in-person dermatologist and the remote dermatologist. Although initially blinded to the diagnoses, both providers were in 100% concordance in the lesion designation as either benign or malignant [244]. Figure 17 depicts the basic configuration utilized in the study’s teledermoscopy protocol. This innovative research study underscores the comparable diagnostic accuracy of teledermoscopy and illustrates the potential for cost-savings in the US MHS [244].

A systematic review found that the accuracy of diagnosis increases up to 15% with the use of teledermoscopy for SCC and BCC [246]. In addition, teledermoscopy allows the provider to discriminate between malignant and benign pigmented lesions. This added diagnostic benefit can help in the assessment of medically necessary mission evacuations. Benefits of synchronous teledermoscopy include the opportunity to interview the patient in real-time. Such interactions may lead to improved efficiency and increased patient satisfaction [243,244,246].

History 

Telemedicine was utilized in the US long before being streamlined enough for military use. Prior to the development of television, radios and telephones were used by doctors to send medical advice or monitor their patients from afar [237,243]. In the early to mid-1900s, television became popular and was eventually used by providers to send visual medical information across the globe. The creation of the Internet was significant in the development of more modern mechanisms for telemedicine, allowing high-resolution images to be sent from one location to another instantly [243].

For over 25 years, telemedicine has been a fundamental aspect of healthcare delivery for the US Department of Defense [5]. Telemedicine was first utilized in the MHS in 1992 in Somalia [5]. At this time, only radio, telephone, fax, and early video conferencing were available, so the efficacy of telemedicine consults was limited [6]. In 1993, video teleconferencing was used by the US military for the first time to provide healthcare to soldiers in the South Pacific [5]. In 1998, the Telemedicine and Advances Technology Research Center (TATRC) was created, which significantly contributed to the development of new technology that would further facilitate telemedicine. It was also the location where many personnel were trained to use the telemedicine equipment [5].

In 2004, a program within the US military was developed using Army Knowledge Online (AKO), which utilizes an S&F system via email to share images of a patient’s skin lesions along with clinical history with military dermatologists to review [6,237]. This system is available to all active duty, reserves, and National Guard soldiers [5]. The dermatologists can then diagnose and create treatment plans on their own time without the PCP standing by. This does not require patients to make and attend appointments and instead is more flexible for both the patient and the provider. Between 2004-2012, AKO provided access to teledermatology 10,817 times. AKO was in existence until 2017 and contributed largely to the growth of telemedicine worldwide [6].

Other systems were also developed for telemedicine delivery, including the Pacific Asynchronous TeleHealth (PATH) [6]. PATH provided visibility of specialist caseload in the electronic medical record. This caseload visibility improved the efficiency of care with faster communication between the specialist and the PCP or the patient [6]. PATH was centered in Hawaii and served islands of the Pacific. A similar platform to PATH, Health Experts Online Portal (HELP) served US Navy soldiers and PCPs worldwide [6]. PATH and HELP platforms were merged into Global Teleconsultation Portal (GTP). Enacted in 2021, GTP is an asynchronous virtual medical center (VMC) that delivers provider-to-provider teleconsultation and patient support [247-249].

GTP operates as a worldwide-accessible, Health Insurance Portability and Accountability Act (HIPAA)-compliant, secure, web-based system for military healthcare personnel and providers [247-249]. GTP has been widely used to provide access to medical specialists, such as board-certified military dermatologists [247-249] GTP can operate on a web browser with limited Internet bandwidth, affording the unique opportunity to be utilized at all military treatment levels [247-249].

Efficacy

Teledermatology has comparable diagnostic accuracy to in-person dermatological care, with the latter being 11% more accurate [243]. However, this discrepancy has been reduced significantly with recent technological advancements in the field of teledermatology. Moreover, teledermatology is an efficient platform that allows dermatologists to see more patients in less time [245]. A systematic review of S&F found that interobserver agreement (arriving at the same diagnosis or differential diagnoses) between in-person and teledermatology was high [246]. Both S&F and LI modalities have been shown to reduce or remove the need for most in-person visits. Patient satisfaction with teledermatology consults was also comparable to in-person visits [246].

A study from 2005 found that the majority of referring physicians (92%), dermatologists (75%), and patients (82%) were found to be satisfied with the teledermatology care received. In addition, 41.5% of patients preferred teledermatology compared to 36.5% that preferred in-person care, and 22% that did not have a preference [245]. Despite limitations, teledermatology is a viable and effective option that can be utilized during deployment when in-person care is not an option.

Teledermatology is a practical and efficient tool that requires continuous refinement. Remote care providers may require additional biopsy equipment and microscopes to send specimens abroad. Biopsies are essential in diagnosing malignant lesions. Likewise, teledermoscopy has been gaining significant interest among dermatologists in recent years. With technological advancements in webcam video quality and phone cameras, dermatologists can have sufficient visual of skin lesions to make a definitive diagnosis. Teledermoscopy can also increase a physician’s ability to remotely triage patients with pigmented skin lesions. Teledermoscopy can provide great utility in determining if a lesion is benign or malignant [245]. Drawbacks to using teledermatology (compared to in-person visits) include technical issues, insufficient training, increased physician workload, or skepticism about technological efficacy [237,240]. Remote healthcare also removes the ability of a provider to palpate the location of the lesion, which may aid in lesion diagnosis [237]. However, solutions can be developed in response to these obstacles. As such, teledermatology plays a necessary and valuable role in the US MHS.

Future directions

Teledermatology is suited to supplement in-person care, educate PCPs, and allow for timely triage. Likewise, teledermatology can help prevent service members from being medically evacuated or help determine when prompt evacuations are medically-necessary [250]. Autonomous care, using machine learning and mathematical modeling, is another potential solution to treating soldiers in remote locations [6]. These systems for autonomous care are currently being developed by the US Army's TATRC and Medical Research and Material Command (MRMC) [6]. Autonomous healthcare can also reduce the risk of interrupted communications [6]. Furthermore, this system could also help triage to determine if evacuation is needed or provide treatment to prevent the need for evacuation. As autonomous systems undergo testing, it has been found that artificial intelligence (AI) has been found to have a 98% correct diagnosis rate for psoriasis and 84% predictive value for AD [6].

Discussion

Operational dermatology serves paramount importance during military missions and deployment. Overlooked skin dermatoses can severely impact the military mission and combat effectiveness [8]. Despite the high prevalence of cutaneous conditions among service members, the shortage of military dermatologists in all branches continues to be an obstacle to timely and effective treatment [8]. When serving operational tours, military dermatologists are often deployed as general medical officers or field surgeons [251]. This underutilization of consulting dermatologists in the field can lead to the misdiagnosis of skin conditions by non-dermatologists [251].

Historical events highlight the utility of operational dermatology in a combat setting. The American Expeditionary Force (AEF) during World War I employed two special dermatology consultants [252]. Additionally, the AEF implemented two field hospitals that operated as specialized dermatology hospitals. The specialized focus on occupational skin conditions enabled soldiers to return to the fighting force after a short recovery period and in full capacity to fulfill the mission at hand. However, the AEF had no consultant dermatologists or dermatology hospitals during World War II. As such, preventable skin diseases caused serious disabilities among soldiers during combat, which negatively affected mission outcomes [252]. Such skin disabilities could have been prevented if military warfighters had received timely evaluation and treatment by dermatologists [252]. These historical pitfalls underscore the critical need for specialized dermatology care in the military [252].

With rapidly advancing technology improvements, teledermatology has been shown to be an efficient and cost-effective alternative to in-field dermatologists [237]. Teledermoscopy has gained significant momentum in teledermatology practice. Given its practicality, convenience, and accessibility, teledermoscopy can improve operational readiness and decrease the need for unnecessary medical evacuations [244,246]. Teledermoscopy enables trained medical corpsmen to send still and live images of skin conditions and histological findings to military dermatologists in the US. When provided accurate medical history and physical exam findings, remote dermatologists can offer patients timely treatment and effective management plans. As such, teledermatology reduces the number of medical evacuations related to skin diseases in the military [237].

Teledermatology effectively provides timely consults. A retrospective study by Hwang et al. assessed the speed at which Department of Defense teledermatology consults from 2004 to 2012 provided diagnosis and treatment to soldiers in combat zones [237]. They found that military dermatologists in the US responded to teledermatology consults in Afghanistan in less than six hours from the request time. These data highlight the effectiveness in teledermatology as a modality to diagnose and treat a higher volume of patients compared to in-person consultations [237]. Delivery of prompt, specialized dermatological care for deployed personnel has a significant potential to improve continuity of quality care in military medicine [237]. If widely implemented, teledermatology will greatly improve combat readiness and decrease medical evacuations.

Furthermore, the implementation of mental health consultation may improve the quality of care and military readiness among service members with occupational dermatoses [253]. Certain dermatoses can cause mild to severe cosmetic disfigurements. Such cutaneous disfigurements induce or exacerbate depression, anxiety, and impair the ability to manage stress. Disfiguring dermatoses can negatively impact self-esteem and perception of self [253]. The term "CBI" has been coined to describe the perception of individuals with regard to cutaneous conditions [251]. Research has shown that patients with positive CBI have a better QOL and outcomes compared to patients with negative CBI [253,254]. Likewise, skin conditions with prominent cosmetic disfigurement (i.e., HS, rosacea, psoriasis, and eczema) have low interpersonal sensitivity (IS). IS refers to feelings of inferiority and social exclusion associated with CBI dissatisfaction [252]. Increased IS due to CBI dissatisfaction has been associated with increased suicidal ideation and intentional self-injury. From a multifactorial standpoint, deployed personnel (especially combat veterans) are among the highest at-risk populations to experience suicidal ideations, attempts, and completions [255]. As such, it is imperative for dermatologists to recognize signs of CBI dissatisfaction and provide multidisciplinary dermatological treatment that includes mental healthcare professionals [253].

Dermatologists provide vital medical care in military operations and emergency settings. Since the onset of the COVID-19 pandemic due to Severe Acute Respiratory Syndrome Coronavirus 2 (SARS-CoV-2), dermatologists have been recruited to identify newly emergent cutaneous conditions [256]. Their specialized training led to the creation of a new classification of skin lesions based on underlying etiologies associated with the COVID-19 pandemic (i.e., viral causes, pneumonia exacerbation, or drug eruptions) [257]. These contributions underscore the adaptability and utility of dermatologists in global pandemics. 

With the ongoing SARS-CoV-2 pandemic and the recent emergence of the mutated monkeypox virus, novel and infectious cutaneous conditions continue to emerge [256-259]. Monkeypox virus belongs to the Orthopoxvirus genus in the family Poxviridae [257]. Clinically, Monkeypox virus infection presents as a rash evolving from macules to papules. The maculopapular rash transforms into vesicles and forms pustular lesions which ultimately scab and crust. The monkeypox virus rash classically emerges on the face and spreads to the extremities. Other rash presentations have been reported, including spread from the groin to other parts of the body [259]. The smallpox vaccine may theoretically provide protection against the monkeypox virus, but more safety data are needed to confirm the efficacy of the proposed immunity. The first confirmed case of monkeypox in an active-duty US service member was confirmed on June 10, 2022, at Stuttgart Army Health Clinic in Germany. As of July 2022, four cases of Monkeypox have been identified across the US Department of Defense. The Navy and Marine Corps Public Health Center (NMCPHC) created a specialized reporting system to ensure situational awareness and Department of Defense-wide collation and tracking of the disease. According to NMCPHC officials, the risk of contracting a monkeypox infection among military service members remains low at this time [259,260]. As the global landscape continues to evolve with novel infections and increased threats of bioterrorism, the critical need for military dermatologists to diagnose and treat operational skin lesions continues to rise.

## Conclusions

Cutaneous conditions among military service members have significant impacts on operational effectiveness and mission evacuations. Deployed personnel are particularly prone to situational factors that trigger or exacerbate dermatoses, such as austere environmental conditions and profound psychosocial stressors. A multidisciplinary approach that incorporates preventative care, patient education, mental health support, teledermoscopy utilization, and specialized dermatologic care will reduce the incidence and prevalence of cutaneous conditions among military personnel while preserving operational costs. 

The shortage of military dermatologists remains a significant barrier in the delivery of prompt dermatologic care. A long-term solution to this obstacle in specialized care includes a widespread expansion of graduate medical education training to increase the total number of military-trained dermatologists. A readily available solution to the limited quantity of military dermatologists includes increased expansion of teledermatology and new integration of teledermoscopy into the US MHS. Teledermatology empowers military dermatologists with the opportunity to provide timely consultations. As military personnel continue to deploy worldwide, occupational skin conditions will inevitably persist, underscoring the duty to provide prompt dermatological care via telemedicine.

As the global landscape continues to shift in a pandemic era with novel infections and cutaneous conditions, the demand for military dermatologists continues to rise exponentially. Through advanced clinical discernment, innovative treatments, and specialized diagnostic modalities, military dermatologists play an irreplaceable role in the care of military service members. As the ongoing need for dermatological care increases at faster rates than provider availability, military dermatologists and teledermatology remain a precious resource in the US MHS. 

## References

[1] Arnold JG, Michener MD (2008). Evaluation of dermatologic conditions by primary care providers in deployed military settings. Mil Med.

[2] Gelman AB, Norton SA, Valdes-Rodriguez R, Yosipovitch G (2015). A review of skin conditions in modern warfare and peacekeeping operations. Mil Med.

[3] Burke KR, Larrymore DC, Cho S (2019). Treatment consideration for US military members with skin disease. Cutis.

[4] Riemenschneider K, Liu J, Powers JG (2018). Skin cancer in the military: a systematic review of melanoma and nonmelanoma skin cancer incidence, prevention, and screening among active duty and veteran personnel. J Am Acad Dermatol.

[5] Rosenbaum BE, Campion CH, Cohen JM, Latkowski JA (2017). The Department of Defense: pioneers of early teledermatology. Dermatol Online J.

[6] Schafrank LA, Falkner RC, Lam TK, Meyerle JH (2021). Teledermatology in military settings. Curr Derm Rep.

[7] Lim HW, Collins SA, Resneck JS Jr (2017). The burden of skin disease in the United States. J Am Acad Dermatol.

[8] Chong WS (2013). Dermatology in the military field: what physicians should know?. World J Clin Cases.

[9] Desai B, McKoy K, Kovarik C (2010). Overview of international teledermatology. Pan Afr Med J.

[10] Ibrahim F, Khan T, Pujalte GG (2015). Bacterial skin infections. Prim Care.

[11] Lamb L, Morgan M (2013). Skin and soft tissue infections in the military. J R Army Med Corps.

[12] Stahlman S, Williams VF, Oh GT, Tribble DR, Millar EV (2021). Skin and soft tissue infections, active component, U.S. Armed Forces, January 2016-September 2020. MSMR.

[13] Ellis MW, Schlett CD, Millar EV (2014). Hygiene strategies to prevent methicillin-resistant Staphylococcus aureus skin and soft tissue infections: a cluster-randomized controlled trial among high-risk military trainees. Clin Infect Dis.

[14] Bläckberg A, Trell K, Rasmussen M (2015). Erysipelas, a large retrospective study of aetiology and clinical presentation. BMC Infect Dis.

[15] Michael Y, Shaukat NM (2022). Erysipelas. https://www.ncbi.nlm.nih.gov/books/NBK532247/.

[16] Clebak KT, Malone MA (2018). Skin infections. Prim Care.

[17] Durdu M, Ilkit M (2013). First step in the differential diagnosis of folliculitis: cytology. Crit Rev Microbiol.

[18] Laureano AC, Schwartz RA, Cohen PJ (2014). Facial bacterial infections: folliculitis. Clin Dermatol.

[19] Weiss AN, Arballo OM, Miletta NR, Wohltmann WE (2018). Military grooming standards and their impact on skin diseases of the head and neck. Cutis.

[20] Cole C, Gazewood JD (2007). Diagnosis and treatment of impetigo. Am Fam Physician.

[21] Bowen AC, Mahé A, Hay RJ, Andrews RM, Steer AC, Tong SY, Carapetis JR (2015). The global epidemiology of impetigo: a systematic review of the population prevalence of impetigo and pyoderma. PLoS One.

[22] Bangert S, Levy M, Hebert AA (2012). Bacterial resistance and impetigo treatment trends: a review. Pediatr Dermatol.

[23] Hartman-Adams H, Banvard C, Juckett G (2014). Impetigo: diagnosis and treatment. Am Fam Physician.

[24] Nardi NM, Schaefer TJ, Espil MO (2022). Impetigo (Nursing). https://www.ncbi.nlm.nih.gov/books/NBK568809/.

[25] Ren Z, Silverberg JI (2021). Burden, risk factors, and infectious complications of cellulitis and erysipelas in US adults and children in the emergency department setting. J Am Acad Dermatol.

[26] Sullivan T, de Barra E (2018). Diagnosis and management of cellulitis. Clin Med J.

[27] Raff AB, Kroshinsky D (2016). Cellulitis: a review. JAMA.

[28] Schmitz GR, Gottlieb M (2021). Managing a cutaneous abscess in the emergency department. Ann Emerg Med.

[29] Fritz SA, Shapiro DJ, Hersh AL (2020). National trends in incidence of purulent skin and soft tissue infections in patients presenting to ambulatory and emergency department settings, 2000-2015. Clin Infect Dis.

[30] Mohamedahmed AY, Zaman S, Stonelake S, Ahmad AN, Datta U, Hajibandeh S, Hajibandeh S (2021). Incision and drainage of cutaneous abscess with or without cavity packing: a systematic review, meta-analysis, and trial sequential analysis of randomised controlled trials. Langenbecks Arch Surg.

[31] Wang JM, Lim HK (2014). Necrotizing fasciitis: eight-year experience and literature review. Braz J Infect Dis.

[32] Goh T, Goh LG, Ang CH, Wong CH (2014). Early diagnosis of necrotizing fasciitis. Br J Surg.

[33] Shimizu T, Tokuda Y (2010). Necrotizing fasciitis. Intern Med.

[34] American Academy of Dermatology Association.: Impetigo (2022). Impetigo: diagnosis and treatment. https://www.aad.org/public/diseases/a-z/impetigo-treatment.

[35] Misiakos EP, Bagias G, Patapis P, Sotiropoulos D, Kanavidis P, Machairas A (2014). Current concepts in the management of necrotizing fasciitis. Front Surg.

[36] (2022). Epidemiology & risk factors. https://www.cdc.gov/parasites/leishmaniasis/epi.html.

[37] (2022). Leishmaniasis. https://www.who.int/health-topics/leishmaniasis.

[38] Rowland T, Davidson SA, Kobylinski K, Menses C, Rowton E (2015). Efficacy of permethrin treated bed nets against leishmania major infected sand flies. US Army Med Dep J.

[39] Stahlman S, Williams VF, Taubman SB (2017). Incident diagnoses of leishmaniasis, active and reserve components, U.S. Armed Forces, 2001-2016. MSMR.

[40] Herwaldt BL (1999). Leishmaniasis. Lancet.

[41] de Vries HJ, Reedijk SH, Schallig HD (2015). Cutaneous leishmaniasis: recent developments in diagnosis and management. Am J Clin Dermatol.

[42] Shirian S, Oryan A, Hatam GR, Daneshbod Y (2013). Three Leishmania/L. species - L. infantum, L. major, L. tropica - as causative agents of mucosal leishmaniasis in Iran. Pathog Glob Health.

[43] Lawn SD, Whetham J, Chiodini PL, Kanagalingam J, Watson J, Behrens RH, Lockwood DN (2004). New world mucosal and cutaneous leishmaniasis: an emerging health problem among British travellers. QJM.

[44] Beiter KJ, Wentlent ZJ, Hamouda AR, Thomas BN (2019). Nonconventional opponents: a review of malaria and leishmaniasis among United States Armed Forces. PeerJ.

[45] (2022). Leishmania Rapid Diagnostic Device Receives FDA clearance. https://www.army.mil/article/138447/leishmania_rapid_diagnostic_device_receives_fda_clearance.

[46] Ghorbani M, Farhoudi R (2018). Leishmaniasis in humans: drug or vaccine therapy?. Drug Des Devel Ther.

[47] Orsborne J, DeRaedt Banks S, Hendy A (2016). Personal Protection of Permethrin-Treated Clothing against Aedes aegypti, the Vector of Dengue and Zika Virus, in the Laboratory. PLoS One.

[48] González AM, Solís-Soto MT, Radon K (2017). Leishmaniasis: who uses personal protection among military personnel in colombia?. Ann Glob Health.

[49] Aronson N, Herwaldt BL, Libman M (2016). Diagnosis and treatment of leishmaniasis: Clinical practice guidelines by the infectious diseases society of America (IDSA) and the American Society of Tropical Medicine and Hygiene (ASTMH). Clin Infect Dis.

[50] Yardley V, Croft SL (2000). A comparison of the activities of three amphotericin B lipid formulations against experimental visceral and cutaneous leishmaniasis. Int J Antimicrob Agents.

[51] Wortmann GW, Fraser SL, Aronson NE, Davis C, Miller RS, Jackson JD, Oster CN (1998). Failure of amphotericin B lipid complex in the treatment of cutaneous leishmaniasis. Clin Infect Dis.

[52] Mackowiak PA (2004). A US Soldier Who Returned from Iraq with Nonhealing Sores. Clinical Infectious Diseases.

[53] Gan WH (2022). Dermatological presentations in military conscripts. https://military-medicine.com/article/3174-dermatological-presentations-in-military-conscripts.html.

[54] Sahoo AK, Mahajan R (2016). Management of tinea corporis, tinea cruris, and tinea pedis: a comprehensive review. Indian Dermatol Online J.

[55] Kovitwanichkanont T, Chong AH (2019). Superficial fungal infections. Aust J Gen Pract.

[56] Ilkit M, Durdu M (2015). Tinea pedis: the etiology and global epidemiology of a common fungal infection. Crit Rev Microbiol.

[57] Ely JW, Rosenfeld S, Seabury Stone M (2014). Diagnosis and management of tinea infections. Am Fam Physician.

[58] Ongsri P, Bunyaratavej S, Leeyaphan C, Pattanaprichakul P, Ongmahutmongkol P, Ariyatanasuporn N, Kulthanan K (2018). Efficacy of antifungal cream versus powder in the treatment of fungal foot skin infection and unpleasant foot odor at medial department of thai naval rating school. Southeast Asian J Trop Med Public Health.

[59] Khamparia A, Singh PK, Rani P, Samanta D, Khanna A, Bhushan B (2021). An internet of health things-driven deep learning framework for detection and classification of skin cancer using transfer learning. Transactions on Emerging Telecommunications Technologies.

[60] Apalla Z, Nashan D, Weller RB, Castellsagué X (2017). Skin cancer: epidemiology, disease burden, pathophysiology, diagnosis, and therapeutic approaches. Dermatol Ther (Heidelb).

[61] AAD AAD (2022). What to look for: ABCDEs of melanoma. https://www.aad.org/public/diseases/skin-cancer/find/at-risk/abcdes.

[62] Bonerandi JJ, Beauvillain C, Caquant L (2011). Guidelines for the diagnosis and treatment of cutaneous squamous cell carcinoma and precursor lesions. J Eur Acad Dermatol Venereol.

[63] Dodds A, Chia A, Shumack S (2014). Actinic keratosis: rationale and management. Dermatol Ther (Heidelb).

[64] Kwiek B, Schwartz RA (2016). Keratoacanthoma (KA): an update and review. J Am Acad Dermatol.

[65] Segatto MM, Botton EU (2018). Precursor lesions of skin cancer. Dermatology in Public Health Environments: A Comprehensive Textbook.

[66] Phulari RG, Rathore R, Talegaon TP, Shah A (2018). Cutaneous horn: a mask to underlying malignancy. J Oral Maxillofac Pathol.

[67] Welch ML, Anderson LL, Grabski WJ (1999). Evaluation and management of nonmelanoma skin cancer: the military perspective. Dermatologic Clinics.

[68] Simões MC, Sousa JJ, Pais AA (2015). Skin cancer and new treatment perspectives: a review. Cancer Lett.

[69] Sheha MA, Mabrouk MS, Sharawy A (2012). Automatic detection of melanoma skin cancer using texture analysis. Int J Comput Appl.

[70] Durmishi A, Fida M, Hoxha S, Naqo X, Bardhi B, Xhelili M, Vasili E (2020). Are military personnel at a more risk for skin cancers?. Dermatol Ther.

[71] Gall R, Bongiorno M, Handfield K (2021). Skin cancer in the US military. Cutis.

[72] Wilkison BD, Wong EB (2017). Skin cancer in military pilots: a special population with special risk factors. Cutis.

[73] Oba J, Woodman SE (2021). The genetic and epigenetic basis of distinct melanoma types. J Dermatol.

[74] Egger ME, Stepp LO, Callender GG (2013). Outcomes and prognostic factors in superficial spreading melanoma. Am J Surg.

[75] Kasprzak JM, Xu YG (2015). Diagnosis and management of lentigo maligna: a review. Drugs Context.

[76] Argenziano G, Longo C, Cameron A (2011). Blue-black rule: a simple dermoscopic clue to recognize pigmented nodular melanoma. Br J Dermatol.

[77] Zbytek B, Carlson JA, Granese J, Ross J, Mihm MC Jr, Slominski A (2008). Current concepts of metastasis in melanoma. Expert Rev Dermatol.

[78] Powers JG, Patel NA, Powers EM, Mayer JE, Stricklin GP, Geller AC (2015). Skin cancer risk factors and preventative behaviors among United States military veterans deployed to Iraq and Afghanistan. J Invest Dermatol.

[79] Xu S, Kwa M, Agarwal A, Rademaker A, Kundu RV (2016). Sunscreen product performance and other determinants of consumer preferences. JAMA Dermatol.

[80] Damian DL (2017). Nicotinamide for skin cancer chemoprevention. Australas J Dermatol.

[81] Jiyad Z, Plasmeijer EI, Keegan S, Samarasinghe V, Green AC, Akhras V (2022). Defining the validity of skin self-examination as a screening test for the detection of suspicious pigmented lesions: a meta-analysis of diagnostic test accuracy. Dermatology.

[82] Börve A, Terstappen K, Sandberg C, Paoli J (2013). Mobile teledermoscopy-there's an app for that!. Dermatol Pract Concept.

[83] Janda M, Horsham C, Vagenas D (2020). Accuracy of mobile digital teledermoscopy for skin self-examinations in adults at high risk of skin cancer: an open-label, randomised controlled trial. Lancet: Digit Health.

[84] Kim JY, Kozlow JH, Mittal B, Moyer J, Olenecki T, Rodgers P (2018). Guidelines of care for the management of cutaneous squamous cell carcinoma. J Am Acad Dermatol.

[85] Swetter SM, Tsao H, Bichakjian CK (2019). Guidelines of care for the management of primary cutaneous melanoma. J Am Acad Dermatol.

[86] Zuberbier T, Aberer W, Asero R (2018). The EAACI/GA²LEN/EDF/WAO guideline for the definition, classification, diagnosis and management of urticaria. Allergy.

[87] Antia C, Baquerizo K, Korman A, Bernstein JA, Alikhan A (2018). Urticaria: a comprehensive review—epidemiology, diagnosis, and work-up. J Am Acad Dermatol.

[88] Schaefer P (2017). Acute and chronic urticaria: evaluation and treatment. Am Fam Physician.

[89] Wedi B, Wieczorek D, Raap U, Kapp A (2014). Urticaria. J German Soc Dermatol.

[90] (2020). Kanerva’s Occupational Dermatology.

[91] Saini SS, Kaplan AP (2018). Chronic spontaneous urticaria: the devil's itch. J Allergy Clin Immunol Pract.

[92] Kulthanan K, Tuchinda P, Chularojanamontri L (2016). Clinical practice guideline for diagnosis and management of urticaria. Asian Pac J Allergy Immunol.

[93] Rosenberg A, Meyerle J (2017). The use of apremilast to treat psoriasis during deployment. Mil Med.

[94] Cashman MW, Reutemann PA, Ehrlich A (2012). Contact dermatitis in the United States: epidemiology, economic impact, and workplace prevention. Dermatol Clin.

[95] Cohen DE, Heidary N (2004). Treatment of irritant and allergic contact dermatitis. Dermatol Ther.

[96] Litchman G, Nair PA, Atwater AR, Bhutta BS (2022). Contact dermatitis. https://www.ncbi.nlm.nih.gov/books/NBK459230/.

[97] Slodownik D, Lee A, Nixon R (2008). Irritant contact dermatitis: a review. Australas J Dermatol.

[98] Dever TT, Walters M, Jacob S (2011). Contact dermatitis in military personnel. Dermatitis.

[99] Johansen JD, Aalto-Korte K, Agner T (2015). European Society of Contact Dermatitis guideline for diagnostic patch testing: recommendations on best practice. Contact Dermatitis.

[100] NIOSH - CDC (2022). National occupational research agenda. Allergic and irritant dermatitis. https://www.cdc.gov/niosh/docs/96-115/diseas.html.

[101] Trattner A, Lazarov A, Ingber A (2020). Military personnel. Kanerva’s Occupational Dermatology.

[102] Goon AT, Ng PP, Ng SK (1999). Allergic contact dermatitis from military camouflage. Contact Dermatitis.

[103] Ludmann P (2022). Eczema types: contact dermatitis diagnosis and treatment. https://www.aad.org/public/diseases/eczema/types/contact-dermatitis/treatment.

[104] Drake LA, Dorner W, Goltz RW (1995). Guidelines of care for contact dermatitis. J Am Acad Dermatol.

[105] Saary J, Qureshi R, Palda V, DeKoven J, Pratt M, Skotnicki-Grant S, Holness L (2005). A systematic review of contact dermatitis treatment and prevention. J Am Acad Dermatol.

[106] (2022). NIOSH alert on work-related latex allergy recommends steps to reduce exposures. https://www.cdc.gov/niosh/updates/latexpr.html.

[107] Usatine RP, Riojas M (2010). Diagnosis and management of contact dermatitis. Am Fam Physician.

[108] Frazier W, Bhardwaj N (2020). Atopic dermatitis: diagnosis and treatment. Am Fam Physician.

[109] Chiesa Fuxench ZC, Block JK, Boguniewicz M (2019). Atopic dermatitis in America study: a cross-sectional study examining the prevalence and disease burden of atopic dermatitis in the US adult population. J Invest Dermatol.

[110] Boguniewicz M, Leung DY (2011). Atopic dermatitis: a disease of altered skin barrier and immune dysregulation. Immunol Rev.

[111] Strom MA, Silverberg JI (2016). Utilization of preventive health care in adults and children with eczema. Am J Prev Med.

[112] Committee on the Assessment of Resiliency and Prevention Programs for Mental and Behavioral Health in Service Members and Their Families;, Board on the Health of Select Populations, Institute of Medicine (2014). Understanding psychological health in the military. Preventing Psychological Disorders in Service Members and Their Families: An Assessment of Programs.

[113] Leung DY (1993). Role of IgE in atopic dermatitis. Curr Opin Immunol.

[114] AAD AAD (2022). Atopic dermatitis clinical guideline. https://www.aad.org/member/clinical-quality/guidelines/atopic-dermatitis.

[115] Alexander H, Paller AS, Traidl-Hoffmann C (2020). The role of bacterial skin infections in atopic dermatitis: expert statement and review from the International Eczema Council Skin Infection Group. Br J Dermatol.

[116] Liaw FY, Huang CF, Hsueh JT, Chiang CP (2012). Eczema herpeticum: a medical emergency. Can Fam Physician.

[117] Xiao A, Tsuchiya A (2022). Eczema Herpeticum. https://www.ncbi.nlm.nih.gov/books/NBK560781/.

[118] Faergemann J (2002). Atopic dermatitis and fungi. Clin Microbiol Rev.

[119] Eichenfield LF, Tom WL, Berger TG (2014). Guidelines of care for the management of atopic dermatitis: section 2. management and treatment of atopic dermatitis with topical therapies. J Am Acad Dermatol.

[120] El Hachem M, Di Mauro G, Rotunno R (2020). Pruritus in pediatric patients with atopic dermatitis: a multidisciplinary approach - summary document from an Italian expert group. Ital J Pediatr.

[121] Garritsen FM, Brouwer MW, Limpens J, Spuls PI (2014). Photo(chemo)therapy in the management of atopic dermatitis: an updated systematic review with implications for practice and research. Br J Dermatol.

[122] Sidbury R, Davis DM, Cohen DE (2014). Guidelines of care for the management of atopic dermatitis: section 3. Management and treatment with phototherapy and systemic agents. J Am Acad Dermatol.

[123] Drucker AM, Eyerich K, de Bruin-Weller MS (2018). Use of systemic corticosteroids for atopic dermatitis: International Eczema Council consensus statement. Br J Dermatol.

[124] Johnson BB, Franco AI, Beck LA, Prezzano JC (2019). Treatment-resistant atopic dermatitis: challenges and solutions. Clin Cosmet Investig Dermatol.

[125] Narla S, Silverberg JI, Simpson EL (2022). Management of inadequate response and adverse effects to dupilumab in atopic dermatitis. J Am Acad Dermatol.

[126] Correale CE, Walker C, Murphy L, Craig TJ (1999). Atopic dermatitis: a review of diagnosis and treatment. Am Fam Physician.

[127] Jeter J, Bowen C (2019). Atopic Dermatitis and Implications for Military Service. Mil Med.

[128] Raharja A, Mahil SK, Barker JN (2021). Psoriasis: a brief overview. Clin Med (Lond).

[129] Kamiya K, Kishimoto M, Sugai J, Komine M, Ohtsuki M (2019). Risk factors for the development of psoriasis. Int J Mol Sci.

[130] Alan Menter MD (2016). Psoriasis and psoriatic arthritis overview. Am J Manag Care.

[131] Armstrong AW, Read C (2020). Pathophysiology, Clinical Presentation, and Treatment of Psoriasis: A Review. JAMA.

[132] Kim WB, Jerome D, Yeung J (2017). Diagnosis and management of psoriasis. Can Fam Physician.

[133] Nahary L, Tamarkin A, Kayam N (2008). An investigation of antistreptococcal antibody responses in guttate psoriasis. Arch Dermatol Res.

[134] Raychaudhuri SK, Maverakis E, Raychaudhuri SP (2014). Diagnosis and classification of psoriasis. Autoimmun Rev.

[135] Michalek IM, Loring B, John SM (2017). A systematic review of worldwide epidemiology of psoriasis. J Eur Acad Dermatol Venereol.

[136] Dand N, Mahil SK, Capon F, Smith CH, Simpson MA, Barker JN (2020). Psoriasis and genetics. Acta Derm Venereol.

[137] Rousset L, Halioua B (2018). Stress and psoriasis. Int J Dermatol.

[138] Ogawa E, Sato Y, Minagawa A, Okuyama R (2018). Pathogenesis of psoriasis and development of treatment. J Dermatol.

[139] Icen M, Crowson CS, McEvoy MT, Dann FJ, Gabriel SE, Maradit Kremers H (2009). Trends in incidence of adult-onset psoriasis over three decades: a population-based study. J Am Acad Dermatol.

[140] AAD AAD (2022). Psoriasis clinical guideline. https://www.aad.org/member/clinical-quality/guidelines/psoriasis.

[141] Bruggink SC, Gussekloo J, Berger MY (2010). Cryotherapy with liquid nitrogen versus topical salicylic acid application for cutaneous warts in primary care: randomized controlled trial. CMAJ.

[142] Menter A, Gelfand JM, Connor C (2020). Joint American Academy of Dermatology-National Psoriasis Foundation guidelines of care for the management of psoriasis with systemic nonbiologic therapies. J Am Acad Dermatol.

[143] Elmets CA, Lim HW, Stoff B (2019). Joint American Academy of Dermatology-National Psoriasis Foundation guidelines of care for the management and treatment of psoriasis with phototherapy. J Am Acad Dermatol.

[144] Hathaway NE, Lyford WH (2021). Apremilast uses and relevance to the military. Cutis.

[145] Al Aboud AM, Nigam PK (2022). Wart. https://www.ncbi.nlm.nih.gov/books/NBK431047/.

[146] Leslie SW, Sajjad H, Kumar S (2022). Genital warts. https://www.ncbi.nlm.nih.gov/books/NBK441884/.

[147] Montgomery AH, Montgomery RM (1948). Mosaic type of plantar wart, its characteristics and treatment. Arch Derm Syphilol.

[148] Laurent R, Kienzler JL, Croissant O, Orth G (1982). Two anatomoclinical types of warts with plantar localization: specific cytopathogenic effects of papillomavirus. Type I (HPV-1) and type 2 (HPV-2). Arch Dermatol Res.

[149] Leiding JW, Holland SM (2012). Warts and all: human papillomavirus in primary immunodeficiencies. J Allergy Clin Immunol.

[150] Institute for Quality and Efficiency in Health (2019). Warts: overview. Care.

[151] Sondermann W, Zimmer L, Schadendorf D, Roesch A, Klode J, Dissemond J (2016). Initial misdiagnosis of melanoma located on the foot is associated with poorer prognosis. Medicine (Baltimore).

[152] Plasencia JM (2000). Cutaneous warts: diagnosis and treatment. Prim Care: Clin Office Pract.

[153] (2022). Human papillomavirus (HPV) treatment and care. https://www.cdc.gov/std/hpv/treatment.htm.

[154] van Brederode RL, Engel ED (2001). Combined cryotherapy/70% salicylic acid treatment for plantar verrucae. J Foot Ankle Surg.

[155] Institute for Quality and Efficiency in Health Care (2019). What are the treatment options for warts?. Health Care.

[156] Veitch D, Kravvas G, Al-Niaimi F (2017). Pulsed Dye Laser Therapy in the Treatment of Warts: A Review of the Literature. Dermatol Surg.

[157] Oni G, Mahaffey PJ (2011). Treatment of recalcitrant warts with the carbon dioxide laser using an excision technique. J Cosmet Laser Ther.

[158] Boroujeni NH, Handjani F (2018). Cryotherapy versus CO2 laser in the treatment of plantar warts: a randomized controlled trial. Dermatol Pract Concept.

[159] Maranda EL, Lim VM, Nguyen AH, Nouri K (2016). Laser and light therapy for facial warts: a systematic review. J Eur Acad Dermatol Venereol.

[160] Benton EC (1997). Therapy of cutaneous warts. Clin Dermatol.

[161] (2022). Monochloroacetic acid. https://inchem.org/documents/pims/chemical/pim352.htm.

[162] Sterling JC, Handfield-Jones S, Hudson PM (2001). Guidelines for the management of cutaneous warts. Br J Dermatol.

[163] Vanhooteghem O, Richert B, de la Brassinne M (2001). Raynaud phenomenon after treatment of verruca vulgaris of the sole with intralesional injection of bleomycin. Pediatr Dermatol.

[164] Ahn CS, Huang WW (2014). Imiquimod in the treatment of cutaneous warts: an evidence-based review. Am J Clin Dermatol.

[165] Khattar JA, Musharrafieh UM, Tamim H, Hamadeh GN (2007). Topical zinc oxide vs. salicylic acid-lactic acid combination in the treatment of warts. Int J Dermatol.

[166] Hekmatjah J, Farshchian M, Grant-Kels JM, Mehregan D (2021). The status of treatment for plantar warts in 2021: No definitive advancements in decades for a common dermatology disease. Clin Dermatol.

[167] Schianchi R, Brena M, Veraldi S (2018). Treatment of common recalcitrant warts with topical formic acid. Int J Dermatol.

[168] Ebrahimi S, Dabiri N, Jamshidnejad E, Sarkari B (2007). Efficacy of 10% silver nitrate solution in the treatment of common warts: a placebo-controlled, randomized, clinical trial. Int J Dermatol.

[169] al Aboosi M (1994). Treatment of plane warts by tretinoin-induced irritant reaction. Int J Dermatol.

[170] Kwok CS, Gibbs S, Bennett C, Holland R, Abbott R (2012). Topical treatments for cutaneous warts. Cochrane Database Syst Rev.

[171] Field S, Irvine AD, Kirby B (2009). The treatment of viral warts with topical cidofovir 1%: our experience of seven paediatric patients. Br J Dermatol.

[172] Broganelli P, Chiaretta A, Fragnelli B, Bernengo MG (2012). Intralesional cidofovir for the treatment of multiple and recalcitrant cutaneous viral warts. Dermatol Ther.

[173] Thappa DM, Chiramel MJ (2016). Evolving role of immunotherapy in the treatment of refractory warts. Indian Dermatol Online J.

[174] Witchey DJ, Witchey NB, Roth-Kauffman MM, Kauffman MK (2018). Plantar warts: epidemiology, pathophysiology, and clinical management. J Am Osteopath Assoc.

[175] Lipke MM (2006). An armamentarium of wart treatments. Clin Med Res.

[176] (2022). Warts: tips for managing. https://www.aad.org/public/diseases/a-z/warts-self-care.

[177] Focht DR 3rd, Spicer C, Fairchok MP (2002). The efficacy of duct tape vs cryotherapy in the treatment of verruca vulgaris (the common wart). Arch Pediatr Adolesc Med.

[178] Purdy S, de Berker D (2011). Acne vulgaris. BMJ Clin Evid.

[179] Williams HC, Dellavalle RP, Garner S (2012). Acne vulgaris. Lancet.

[180] Heng AH, Chew FT (2020). Systematic review of the epidemiology of acne vulgaris. Sci Rep.

[181] Bhate K, Williams HC (2013). Epidemiology of acne vulgaris. Br J Dermatol.

[182] Zaenglein AL (2022). Acne vulgaris. N Engl J Med.

[183] AAD AAD (2022). Acne clinical guideline. https://www.aad.org/member/clinical-quality/guidelines/acne.

[184] Endly DC, Miller RA (2017). Oily skin: a review of treatment options. J Clin Aesthet Dermatol.

[185] Briganti S, Flori E, Mastrofrancesco A, Ottaviani M (2020). Acne as an altered dermato-endocrine response problem. Exp Dermatol.

[186] Chuan SS, Chang RJ (2010). Polycystic ovary syndrome and acne. Skin Therapy Lett.

[187] Juhl CR, Bergholdt HK, Miller IM, Jemec GB, Kanters JK, Ellervik C (2018). Dairy intake and acne vulgaris: a systematic review and meta-analysis of 78,529 children, adolescents, and young adults. Nutrients.

[188] Brahe C, Peters K (2020). Fighting acne for the fighting forces. Cutis.

[189] Juckett G, Hartman-Adams H (2009). Management of keloids and hypertrophic scars. Am Fam Physician.

[190] Berman B, Maderal A, Raphael B (2017). Keloids and hypertrophic scars: pathophysiology, classification, and treatment. Dermatol Surg.

[191] Butzelaar L, Niessen FB, Talhout W, Schooneman DP, Ulrich MM, Beelen RH, Mink van der Molen AB (2017). Different properties of skin of different body sites: the root of keloid formation?. Wound Repair Regen.

[192] Mahdavian Delavary B, van der Veer WM, Ferreira JA, Niessen FB (2012). Formation of hypertrophic scars: evolution and susceptibility. J Plast Surg Hand Surg.

[193] Mokos ZB, Jović A, Grgurević L, Dumić-Čule I, Kostović K, Čeović R, Marinović B (2017). Current therapeutic approach to hypertrophic scars. Front Med (Lausanne).

[194] Tripathi S, Soni K, Agrawal P, Gour V, Mondal R, Soni V (2020). Hypertrophic scars and keloids: a review and current treatment modalities. Biomedical Dermatology.

[195] (2022). Keloids: diagnosis and treatment. https://www.aad.org/public/diseases/a-z/keloids-treatment.

[196] Morelli Coppola M, Salzillo R, Segreto F, Persichetti P (2018). Triamcinolone acetonide intralesional injection for the treatment of keloid scars: patient selection and perspectives. Clin Cosmet Investig Dermatol.

[197] Shah VV, Aldahan AS, Mlacker S, Alsaidan M, Samarkandy S, Nouri K (2016). 5-Fluorouracil in the treatment of keloids and hypertrophic scars: a comprehensive review of the literature. Dermatol Ther (Heidelb).

[198] Westra I, Pham H, Niessen FB (2016). Topical silicone sheet application in the treatment of hypertrophic scars and keloids. J Clin Aesthet Dermatol.

[199] Rathee M, Kundu R, Tamrakar A (2014). Custom made pressure appliance for presurgical sustained compression of auricular keloid. Ann Med Health Sci Res.

[200] Ai JW, Liu JT, Pei SD, Liu Y, Li DS, Lin HM, Pei B (2017). The effectiveness of pressure therapy (15-25 mmHg) for hypertrophic burn scars: a systematic review and meta-analysis. Sci Rep.

[201] Zouboulis CC, Blume U, Büttner P, Orfanos CE (1993). Outcomes of cryosurgery in keloids and hypertrophic scars: a prospective consecutive trial of case series. Arch Dermatol.

[202] O'Boyle CP, Shayan-Arani H, Hamada MW (2017). Intralesional cryotherapy for hypertrophic scars and keloids: a review. Scars Burn Heal.

[203] Gauglitz GG (2013). Management of keloids and hypertrophic scars: current and emerging options. Clin Cosmet Investig Dermatol.

[204] (2022). Medical standards for military service: appointment, enlistment, or induction. https://www.esd.whs.mil/Portals/54/Documents/DD/issuances/dodi/613003_vol1.PDF?ver=7fhqacc0jGX_R9_1iexudA%3D%3D.

[205] Billero V, Miteva M (2018). Traction alopecia: the root of the problem. Clin Cosmet Investig Dermatol.

[206] Korona-Bailey J, Banaag A, Nguyen DR, Pasieka H, Koehlmoos TP (2021). Free the bun: prevalence of alopecia among active duty service women, fiscal years 2010-2019. Mil Med.

[207] Fox GN, Stausmire JM, Mehregan DR (2007). Traction folliculitis: an underreported entity. Cutis.

[208] Khumalo NP, Jessop S, Gumedze F, Ehrlich R (2008). Determinants of marginal traction alopecia in African girls and women. J Am Acad Dermatol.

[209] Goldberg LJ (2009). Cicatricial marginal alopecia: is it all traction?. Br J Dermatol.

[210] Beach RA (2018). Case series of oral minoxidil for androgenetic and traction alopecia: Tolerability & the five C's of oral therapy. Dermatol Ther.

[211] Samrao A, Price VH, Zedek D, Mirmirani P (2011). The "Fringe Sign" - a useful clinical finding in traction alopecia of the marginal hair line. Dermatol Online J.

[212] Callender VD, McMichael AJ, Cohen GF (2004). Medical and surgical therapies for alopecias in black women. Dermatol Ther.

[213] Fu JM, Price VH (2009). Approach to hair loss in women of color. Semin Cutan Med Surg.

[214] May Franklin JM, Wohltmann WE, Wong EB (2021). From buns to braids and ponytails: entering a new era of female military hair-grooming standards. Cutis.

[215] (2021). United States army appearance and grooming modifications. https://armyreup.s3.amazonaws.com/site/wp-content/uploads/2021/02/26142503/ALARACT_015_2021_NEW_GROOMING_STANDARDS.pdf.

[216] (2022). Dress and personal appearance of United States air force and United States space force personnel. https://static.e-publishing.af.mil/production/1/af_a1/publication/dafi36-2903/dafi36-2903.pdf.

[217] (2022). Chapter two: grooming standards—2201 - personal appearance. https://www.mynavyhr.navy.mil/References/US-Navy-Uniforms/Uniform-Regulations/Chapter-2/2201-Personal-Appearance/.

[218] Keeling M, Williamson H, Williams VS, Kiff J, Evans S, Murphy D, Harcourt D (2022). Body image and psychosocial well-being among UK military personnel and veterans who sustained appearance-altering conflict injuries. Mil Psychol.

[219] Alikhan A, Sayed C, Alavi A (2019). North American clinical management guidelines for hidradenitis suppurativa: a publication from the United States and Canadian Hidradenitis Suppurativa Foundations: part I: diagnosis, evaluation, and the use of complementary and procedural management. J Am Acad Dermatol.

[220] Alavi A, Anooshirvani N, Kim WB, Coutts P, Sibbald RG (2015). Quality-of-life impairment in patients with hidradenitis suppurativa: a Canadian study. Am J Clin Dermatol.

[221] Dufour DN, Emtestam L, Jemec GB (2014). Hidradenitis suppurativa: a common and burdensome, yet under-recognised, inflammatory skin disease. Postgrad Med J.

[222] Godiwalla RY, Storie EB, Winn AE (2020). Hidradenitis suppurativa in the military. Cutis.

[223] (2022). Hidradenitis suppurativa: management. https://www.uptodate.com/contents/hidradenitis-suppurativa-management.

[224] (2022). Hidradenitis suppurativa: diagnosis and treatment. https://www.aad.org/public/diseases/a-z/hidradenitis-suppurativa-treatment.

[225] Wieczorek M, Walecka I (2018). Hidradenitis suppurativa - known and unknown disease. Reumatologia.

[226] CDC CDC (2022). Case definitions† for use in the 2022 mpox response. https://www.cdc.gov/poxvirus/monkeypox/clinicians/case-definition.html.

[227] (2022). Human monkeypox -- Kasai oriental, Zaire, 1996-1997. MMWR Morb Mortal Wkly Rep.

[228] Kime P (2022). Number of monkeypox cases in the military climbs tenfold in less than 4 weeks. https://www.military.com/daily-news/2022/08/05/number-of-monkeypox-cases-military-climbs-tenfold-less-4-weeks.html.

[229] (2022). CDC monkeypox response: transmission. https://www.cdc.gov/media/releases/2022/0509-monkeypox-transmission.html.

[230] (2022). Mpox (monkeypox): background information. https://www.gov.uk/guidance/monkeypox.

[231] Mbala PK, Huggins JW, Riu-Rovira T (2017). Maternal and fetal outcomes among pregnant women with human monkeypox infection in the Democratic Republic of Congo. J Infect Dis.

[232] Iñigo Martínez J, Gil Montalbán E, Jiménez Bueno S (2022). Monkeypox outbreak predominantly affecting men who have sex with men, Madrid, Spain, 26 April to 16 June 2022. Euro Surveill.

[233] Thornhill JP, Barkati S, Walmsley S (2022). Monkeypox virus infection in humans across 16 countries - April-June 2022. N Engl J Med.

[234] (2022). Monkeypox. https://www.who.int/news-room/fact-sheets/detail/monkeypox.

[235] Cono J, Casey C, Bell D (2022). Smallpox vaccination and adverse reactions: guidance for clinicians. https://www.cdc.gov/mmwr/preview/mmwrhtml/rr5204a1.htm.

[236] Pala P, Bergler-Czop BS, Gwiżdż JM (2020). Teledermatology: idea, benefits and risks of modern age - a systematic review based on melanoma. Postepy Dermatol Alergol.

[237] Hwang J, Kakimoto C (2018). Teledermatology in the US military: a historic foundation for current and future applications. Cutis.

[238] Trettel A, Eissing L, Augustin M (2018). Telemedicine in dermatology: findings and experiences worldwide - a systematic literature review. J Eur Acad Dermatol Venereol.

[239] Elsner P, Bauer A, Diepgen TL (2018). Position paper: telemedicine in occupational dermatology - current status and perspectives. J Dtsch Dermatol Ges.

[240] Stronge AJ, Nichols T, Rogers WA, Fisk AD (2008). Systematic human factors evaluation of a teledermatology system within the U.S. military. Telemedicine and e-Health.

[241] Gregory JF, Taylor EA, Liu YE, Love TV, Raiciulescu S, Meyerle JH (2019). The burden of skin disease on deployed servicemembers. Mil Med.

[242] Henning JS, Wohltmann W, Hivnor C (2010). Teledermatology from a combat zone. Arch Dermatol.

[243] Coates SJ, Kvedar J, Granstein RD (2015). Teledermatology: from historical perspective to emerging techniques of the modern era: part I: History, rationale, and current practice. J Am Acad Dermatol.

[244] Day WG, Shrivastava V, Roman JW (2020). Synchronous teledermoscopy in military treatment facilities. Mil Med.

[245] Whited JD (2006). Teledermatology research review. Int J Dermatol.

[246] Warshaw EM, Hillman YJ, Greer NL, Hagel EM, MacDonald R, Rutks IR, Wilt TJ (2011). Teledermatology for diagnosis and management of skin conditions: a systematic review. J Am Acad Dermatol.

[247] Reynolds N (2022). Defense health agency (DHA) update on virtual health (VH) for defense health board. https://www.health.mil/Reference-Center/Meeting-References/2022/03/30/Defense-Health-Agency-Update-on-Virtual-Health.

[248] (2022). PATH & HELP to transition to GTP. https://path.tamc.amedd.army.mil/path/pdf/GTP_Transition2.pdf.

[249] (2022). GTP training 101. https://help.nmcp.med.navy.mil/path/help/help.htm.

[250] Giavina-Bianchi M, Santos AP, Cordioli E (2020). Teledermatology reduces dermatology referrals and improves access to specialists. EClinicalMedicine.

[251] Durso TA, Iddins BO, Miletta NR (2019). Combat dermatology: the role of the deployed army dermatologist. Cutis.

[252] Schissel DJ, Wilde JL (2004). Operational dermatology. Mil Med.

[253] Brahe C (2022). Cutaneous body image: how the mental health benefits of treating dermatologic disease support military readiness in service members. Cutis.

[254] Hinkley SB, Holub SC, Menter A (2020). The validity of cutaneous body image as a construct and as a mediator of the relationship between cutaneous disease and mental health. Dermatol Ther (Heidelb).

[255] Pruitt LD, Smolenski DJ, Bush NE, Tucker J, Issa F, Hoyt TV, Reger MA (2019). Suicide in the military: understanding rates and risk factors across the United States' armed forces. Mil Med.

[256] Pendlebury GA, Oro P, Haynes W, Merideth D, Bartling S, Bongiorno MA (2022). The impact of COVID-19 pandemic on dermatological conditions: a novel, comprehensive review. Dermatopathology (Basel).

[257] Freeman EE, Chamberlin GC, McMahon DE (2021). Dermatology COVID-19 registries: updates and future directions. Dermatol Clin.

[258] (2022). Monkeypox resource center. https://www.aad.org/member/clinical-quality/clinical-care/monkeypox.

[259] Kime P: As Monkeypox Outbreak Grows Nationally (2022). As monkeypox outbreak grows nationally, cases remain rare in US military. https://www.military.com/daily-news/2022/07/11/monkeypox-outbreak-grows-nationally-cases-remain-rare-us-military.html.

[260] Navy Medicine (2022). Mpox. https://www.med.navy.mil/Navy-Marine-Corps-Public-Health-Center/Preventive-Medicine/Program-and-Policy-Support/Monkeypox/.

